# Feral populations of *Brassica oleracea* along Atlantic coasts in western Europe

**DOI:** 10.1002/ece3.6821

**Published:** 2020-09-24

**Authors:** Elizabeth A. Mittell, Christina A. Cobbold, Umer Zeeshan Ijaz, Elizabeth A. Kilbride, Karen A. Moore, Barbara K. Mable

**Affiliations:** ^1^ Institute of Biodiversity, Animal Health and Comparative Medicine University of Glasgow Glasgow UK; ^2^ School of Biology University of St Andrews St Andrews UK; ^3^ School of Mathematics and Statistics University of Glasgow Glasgow UK; ^4^ The Boyd Orr Centre for Population and Ecosystem Health University of Glasgow Glasgow UK; ^5^ School of Engineering University of Glasgow Glasgow UK; ^6^ Exeter Sequencing Service University of Exeter Exeter UK

**Keywords:** *Brassica oleracea*, crop wild relatives, domestication, environment–genotype associations, feral populations, isolation by distance

## Abstract

There has been growing emphasis on the role that crop wild relatives might play in supporting highly selected agriculturally valuable species in the face of climate change. In species that were domesticated many thousands of years ago, distinguishing wild populations from escaped feral forms can be challenging, but reintroducing variation from either source could supplement current cultivated forms. For economically important cabbages (Brassicaceae: *Brassica oleracea*), “wild” populations occur throughout Europe but little is known about their genetic variation or potential as resources for breeding more resilient crop varieties. The main aim of this study was to characterize the population structure of geographically isolated wild cabbage populations along the coasts of the UK and Spain, including the Atlantic range edges. Double‐digest restriction‐site‐associated DNA sequencing was used to sample individual cabbage genomes, assess the similarity of plants from 20 populations, and explore environment–genotype associations across varying climatic conditions. Interestingly, there were no indications of isolation by distance; several geographically close populations were genetically more distinct from each other than to distant populations. Furthermore, several distant populations shared genetic ancestry, which could indicate that they were established by escapees of similar source cultivars. However, there were signals of local adaptation to different environments, including a possible relationship between genetic diversity and soil pH. Overall, these results highlight wild cabbages in the Atlantic region as an important genetic resource worthy of further research into their relationship with existing crop varieties.

## Graphical abstract

We investigated the population structure of cabbage (*Brassica oleracea*) populations found around the coasts of the UK and the north coast of Spain, including many populations at the edges of the *B. oleracea* range that have not been seen in the literature before. This study system is particularly interesting because existing evidence suggests that domesticated plants escaped from gardens and fields, and established these wild populations relatively recently across a large environmental gradient. We suggest that these wild cabbage populations hold agriculturally useful genetic resources for an economically important plant genus.

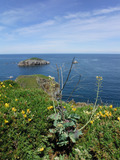

## 1 INTRODUCTION

Domestication was an important transition within human societies, which allowed the rise of civilizations (Diamond, 2002). While vital for human success, there have been evolutionary consequences for the domesticated organisms. In crop plants, the selection of "domestication traits" has led to many desired changes in physiological, morphological, and life‐history traits compared with their wild relatives (Milla et al., 2015; Purugganan & Fuller, 2009). However, traits that are correlated with those selected for (directly or indirectly) can also influence phenotypes via pleiotropic effects (Conner, 2002) and linkage disequilibrium (Falconer & Mackay, 1996). These genetic constraints and narrow population bottlenecks can have unintended genetic consequences for crop plants, particularly elite lines that are the result of intense artificial selection, for example, reduced genetic diversity, increased genetic drift, and increased deleterious allele frequencies (Rauf et al., 2010; von Wettberg et al., 2018). It is also likely that crop lines are constrained to some extent by the environment within which they were originally domesticated. Therefore, to continue to utilize crop plants successfully, it is important to understand both the genetic consequences of domestication, and where it occurred.

A classic example of domestication can be found in the commercially valuable species, *Brassica oleracea* (recognized by Darwin, 1859; Walley et al., 2012). This single species contains a huge amount of morphological diversity in cultivated varieties that has been around since at least the 1^st^ Century (e.g., kale, kohlrabi, broccoli, Brussels sprouts, and cauliflower; Maggioni et al., 2018); the same morphological extremes are not found in wild populations. The origin of domesticated *B. oleracea* crops and the "wild" or "feral" status of populations, found throughout the UK and along the Atlantic coasts of north‐western Europe (Raybould et al., 1999), has been debated in the literature (Allender et al., 2007; Gómez‐Campo & Prakash, 1999; Maggioni, 2015; Mitchell, 1976). Initially, it was thought that different cultivars were independently domesticated from wild populations on European Atlantic coasts (e.g., Spanish cabbage varieties were domesticated from local wild Spanish populations; Gómez‐Campo & Prakash, 1999) and that early domesticates were introduced to and diversified within the Mediterranean region around 3,000–4,000 years ago (Allender et al., 2007). Information was limited when this hypothesis was favored (Allender et al., 2007; Gómez‐Campo & Prakash, 1999), although there was already conflicting evidence (Mitchell, 1976). For example, Mitchell (1976) found that the locations of ancient human settlements and modern *B. oleracea* populations coincided along UK coasts, providing a potential source of escapees from domestic settings (agriculture or gardens) that could have established feral populations. This alternative hypothesis that *B. oleracea* originated elsewhere and escaped into the wild in the Atlantic region has been supported by recent linguistic and historical research (Maggioni, 2015; Maggioni et al., 2018). Maggioni (2015) suggested that the most plausible hypothesis is that *B. oleracea* was domesticated in the Mediterranean region, before being moved across Europe by people, where escaped plants established now naturalized populations. However, the genetic status of *B. oleracea* in the Atlantic region is still an open question (*B. oleracea* is classified as a native species in the UK and an alien species in Spain; Euro + Med PlantBase, 2020).

The ease with which cultivated and wild *B. oleracea* plants can introgress is an issue for interpreting variation within the *B. oleracea* species complex, as past hybridization can obscure phylogeographic signals (Allender et al., 2007). However, for crop breeding purposes a close genetic relationship between wild populations and domesticated cultivars may be seen as an advantage; higher genetic similarity could make it easier to introgress adaptive traits from the wild into cultivated varieties (Hoisington et al., 1999). An alternative view is that if these populations are feral, they would have experienced the same domestication bottleneck as many cultivars (von Wettberg et al., 2018), and therefore, they may not be the important reservoirs of genetic diversity that crop wild relatives are typically assumed to be. Compared to domestication, feralization is underinvestigated; however, modern genomic data are allowing its occurrence to be identified and consequences better understood (see examples in Henriksen et al., 2018). Despite the agricultural importance of *B. oleracea*, there has not yet been a comprehensive genetic analysis of wild populations in the Atlantic region that would allow assessment of their utility as sources of variation for cultivation.

Escaped plants can be thought of as "invasive" species, which are defined as those that became established after introduction outside of the biogeographic region within which they evolved (Prentis et al., 2008). However, it is not always clear where these "native" regions are located, as is the case of *B. oleracea*, or why certain species are successful where others are not. Furthermore, wild populations of *B. oleracea* do not have the characteristics that are thought to be important for successful establishment in novel locations (i.e., "invasive traits"; Funk et al., 2016). For example, wild *B. oleracea* are as follows: perennials rather than annuals, woody rather than herbaceous, relatively slow‐ rather than fast‐growing, and predominantly outcrossing rather than selfing. Self‐fertilization in plants is inhibited by polymorphic self‐incompatibility (SI) recognition systems where haplotype blocks encode distinct proteins for pollen‐pistil recognition (Charlesworth et al., 2005). A strong SI system exists in *B. oleracea* (a single‐locus system with over 60 alleles; Raybould et al., 1999), making them predominantly self‐incompatible (Kitashiba & Nasrallah, 2014; Walley et al., 2012; Yousef et al., 2018). Development of self‐compatible lines can aid in propagation of cultivated forms (e.g., Xiao et al., 2019), but reduce adaptive potential to changing environmental conditions. Therefore, even if the “wild” populations include escaped forms, retention of a wide range of self‐incompatibility alleles could be used to enhance the potential of breeding strategies designed to maintain heterosis.

Currently, too little is known about levels of genetic variation and population structure in wild *B. oleracea* populations to fully assess the potential for use of plants from different regions to supplement crop diversity. Population structure and within‐population genetic diversity are impacted by gene flow, which occurs via pollen and seeds in plants (Scheepens et al., 2012; Slatkin, 1987). The main pollinators of *B. oleracea* are bees that fly short distances between plants (average 2 m; Raybould et al., 1999). Seed dispersal was previously thought to be limited to approximately 4 m (Watson‐Jones et al., 2006). However, Wichmann et al. (2009) found that wind can spread seeds up to 250 m, and that rare‐long distance dispersal events of up to 10 km could occur if seeds became attached to people's shoes. Therefore, although gene flow may be limited between geographically close populations leading to high genetic structuring in some instances, in other cases, such as where plants grow close (0–4 m) to well‐used coastal paths, gene flow might be greater than expected. Genetic diversity estimates have been made in some *B. oleracea* populations within the Atlantic region (e.g., Table 1), but the northern edge (Scotland) has not been investigated. A correlation between genetic distance and geographic distance in wild *B. oleracea* populations was found in some studies (Raybould et al., 1999; Sánchez‐Yélamo, 2014) but not others (Christensen et al., 2011; Watson‐Jones et al., 2006). Interestingly, Watson‐Jones et al. (2006) also considered some environmental variables and found that higher soil pH was associated with lower genetic diversity in English and Welsh populations. The inconsistency in previous studies could be due to the varying spatial scales and molecular markers used. However, overall, these results highlight the uncertainty in the status and genetic contents of wild *B. oleracea* populations in the Atlantic region, as well as the potential effect of environment on the plant genetics. Filling these knowledge gaps could provide important insights into these crop wild relatives for agricultural use.

**Table 1:** Estimates of genetic diversity within wild *Brassica oleracea* populations from previous studies using different molecular markers. *H*_E_ is expected heterozygosity estimated using Nei's gene diversity (Nei, 1973)

Study	Molecular marker	*H*_E_	Populations
Lanner‐Herrera et al. (1996)	Isozymes	0.10–0.56	France, Spain, UK
Lázaro and Aguinagalde (1998)	Isozymes	0.26–0.30	France, Spain, UK
Raybould et al. (1999)	Isozymes	0.40 (0.18–0.41)^a^	UK
Raybould et al. (1999)	Microsatellites	0.36 (0.21–0.33)^a^	UK
Watson‐Jones et al. (2006)	AFLPs	0.19–0.33	UK
Christensen et al. (2011)	AFLPs	0.23, 0.20	Spain, UK
Maggioni et al. (pers. comm. 2019)	AFLPs	0.25	France

^a^ Pooled population *H*_E_ with the range of estimates from individual populations shown in brackets.

*Brassica oleracea* is a good model for investigating the genetic resources available (e.g., the extent of genetic diversity and local adaptation) in a potentially feral crop wild relative because it is diploid and a reference genome is available (Liu et al., 2014). Therefore, compared to other crop species (e.g., polyploids) genetic analyses are simpler. For many questions, whole‐genome sequencing is unnecessary (Rockman, 2012) and reduced‐representation methods, such as double‐digest restriction‐associated DNA sequencing (ddRADseq), are sufficient to: assess genetic diversity within and between populations (Andrews et al., 2016); determine population genetic structuring (Gao et al., 2017); and investigate potential associations between genotypes and environmental variables (Forester et al., 2018). Therefore, ddRADseq is an appropriate method for considering the genetic resources in, and local adaptation of, *B. oleracea* populations across their Atlantic range.

Overall, current knowledge on genetic variation of *B. oleracea* in wild populations is patchy in geographic coverage and based on outdated molecular genetic techniques (Table 1). Therefore, this study combined modern genetic techniques and the reference genome available for this species to increase the power to detect differences among populations across a broad geographic range. The following questions were addressed: (a) How much genetic variation exists among wild populations of *B. oleracea* in the UK and Spain; (b) how are populations structured in the Atlantic region and how much differentiation exists between isolated populations; and (c) are there signals of local adaptation to the environment? The results provide insights into the utility of *B. oleracea* as a crop wild relative genetic resource for agriculture, as well as shed light on the most likely region of *B. oleracea* domestication.

## 2 MATERIALS AND METHODS

**Figure 1:**
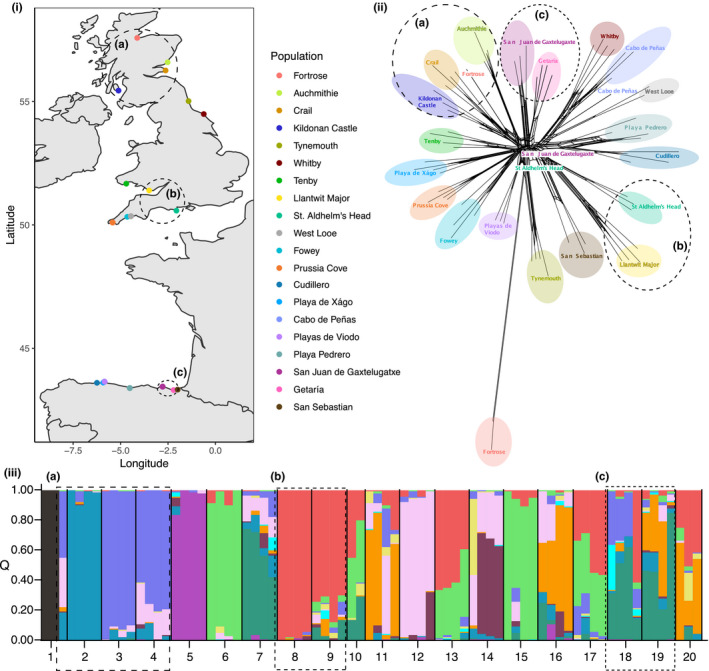
Population structuring of wild populations of *Brassica oleracea*. (i) Location of the populations considered here. (ii) Clustering of samples from RAxML (v8.2; GTRCAT model and 1,000 maximum‐likelihood bootstrap replicates), visualized in SplitsTree4. (iii) STRUCTURE plot illustrating shared genetic ancestry for *K* = 12, ordered by population: 1—Fortrose; 2—Auchmithie; 3—Crail; 4—Kildonan Castle; 5—Tynemouth; 6—Whitby; 7—Tenby; 8—Llantwit Major; 9—St. Aldhelm's Head; 10—West Looe; 11—Fowey; 12—Prussia Cova; 13—Cudillero; 14—Playa de Xágo; 15—Cabo de Peñas; 16—Playas de Viodo; 17—Playa Pedrero; 18—San Juan de Gaztelugatxe; 19—Getaría; 20—San Sebastian. Across the figures the same colors and numbering are used for each population. The dashed lines and letters indicate some clustering: (a) populations in Scotland; (b) populations closest to the Welsh‐English border; and (c) populations in the Basque Country, Spain (excluding San Sebastian)

Twenty‐four populations of *B. oleracea* were chosen from the UK and Spain to cover both a latitudinal and longitudinal gradient of the Atlantic range for genetic analyses (Figure 1 & Table 2). French populations were not sampled here, but are the focus of a recent genetic analysis by Maggioni *et al*. (personal communication). Leaves were collected from four individual plants from each population for DNA extraction, as has been successfully applied to the study of population structure in wild relatives in the Brassicaceae (Buckley et al., 2018). Nazareno et al. (2017) found that compared to “traditional” population genetic markers, these smaller sample sizes are sufficient for various population statistics when large numbers of SNPs are available. The bedrock for each population was obtained from the British Geological Survey (BGS, 2018) and the Instituto Geológico y Minero de España (IGME, 2018). The first year a written record of a population exists was obtained for the UK populations from the Botanical Society of Britain & Ireland (BSBI, 2018). No equivalent records could be found for the Spanish populations.

**Table 2:** A summary of the natural populations of *Brassica oleracea* used in this study, including the following: the bedrock, the first time the population was recorded, the number of individuals sequenced, and the number of individuals included in downstream analyses

Region	Population	Bedrock^a^	First population record^b^	Number sequenced	Number included^c^
ES	Auchmithie	Red basic sandstone	1,913	4	4
ES	Crail	Sandstone & limestone	1,840	4	4
ES	Fortrose	Sandstone	1,968	4	3
WS	Kildonan Castle	Sandstone & limestone	1,987	4	4
NEE	Tynemouth	Sandstone & limestone	1,805	4	4
NEE	Staithes	Shale & sandstone	1,831	4	–
NEE	Whitby	Shale	1,906	4	4
NW	Little Orme	Limestone	1,895	4	–
SW	Tenby	Siltstone & sandstone	1,773	4	4
SW	Llantwit Major	Limestone	1,850	4	4
SWE	Prussia Cove	Slate, shale & siltstone	1,871	4	4
SWE	Fowey	Shale & siltstone	1,805	4	4
SWE	West Looe	Siltstone & sandstone	1,971	4	2
SWE	St. Aldhelm's Head	Limestone	1,933	4	4
A	Cudillero	Slate & sandstone		4	4
A	Playa de Xágo	Sandstone & Dolomite		4	4
A	Cabo de Peñas	Slate & quartzite		4	4
A	Playas de Viodo	Slate & shale		4	4
A	Tazones	Dolomite & limestone		4	–
C	Playa Pedrero	Quartzites		4	4
C	La Franca	Quartzites		4	–
BC	San Juan de Gaztelugatxe	Limestone		4	4
BC	Getaría	Limestone		4	4
BC	San Sebastian	Calcareous sandstone		4	3
			Total	96	76

^a^ Data obtained from the British Geological Survey (https://www.bgs.ac.uk) and the Instituto Geológico y Minero de España (http://www.igme.es). Region codes: ES, East Scotland; WS, West Scotland; NEE, Northeastern England; NW, North Wales; SW, South Wales; SWE, Southwestern England; A, Asturias Spain; C, Cantabrica Spain; BC, Basque Country Spain.

^b^ Data obtained from the Botanical Society of Britain & Ireland (https://bsbi.org).

^c^ Indicates where data were lost in quality filtering of sequences and not included in downstream analyses.

### 2.1 Molecular methods

High‐molecular‐weight DNA was extracted from the leaves of 96 individuals from 24 populations (Table 2) using DNeasy Plant Mini Kits (QIAGEN, Hilden, Germany) and quantified using a Qubit 2.0 Fluorometer (Thermo Fisher Scientific, Waltham, MA, USA). Four samples from each population were sent for library preparation and sequencing at University of Exeter Sequencing Service. Double‐digest RADseq libraries were made using a modification of the method in Wu et al. (2016) that allowed NexteraXT indexes (Illumine Corp., USA) to be used for multiplexing samples. In addition, an RYRY spacer was inserted in the adapter 3′ of the Illumina sequencing primer annealing site to provide additional complexity at the start of read 1 immediately before the Sac1 sticky end. For each sample, 400 ng DNA was fully digested with Sac1 and Mse1 restriction endonucleases and purified using Ampure XP beads. Illumina compatible i5 adapters were designed to ligate to the AGCT‐3′ sticky end left after Sac1 digest, and Illumina compatible i7 adapters were designed to ligate to the 5′‐TA overhangs remaining after Mse1 digest. Adapter‐ligation excess adapters were removed using Ampure XP beads. DNA fragments were amplified by 12 cycles of indexing PCR, purified, size selected (inserts 330–670 bp), and validated using a Tapestation D1000 HS Screentape (Agilent Technologies Ltd). Libraries were equimolar pooled, and the pool concentration was calculated after qPCR. Libraries were denatured, diluted, and sequenced with 125bp paired‐end reads on Illumina HiSeq 2500 using SBS High Output reagents v4 (Illumina Corp., USA).

### 2.2 Data processing

Reads were demultiplexed and trimmed to 100 bp using cutadapt (Martin, 2011). These were then cleaned and quality filtered using the *process_radtags* pipeline in Stacks v1.47 (Rochette & Catchen, 2017). Bowtie (v2; Langmead & Salzberg, 2012) and samtools (v1.9; Li et al., 2009) were used to align the reads to the *B. oleracea* reference genome (Liu et al., 2014). A catalogue of stacks was then created using *ref_map* (Stacks) with the default settings. The *populations* pipeline (Stacks) was used to filter the data, and calculate summary statistics. Three datasets were generated with different filtering parameters depending on the downstream analysis. Firstly, for dataset 1 (*within individuals*), which was used to estimate genetic diversity within individuals and in phylogenetic analyses, all individuals were filtered as a single population, and loci were retained if they had a minimum individual stack depth of five, a minimum minor allele frequency of 0.01, a maximum observed heterozygosity of 0.7 and were present in 60% of individuals. Secondly, dataset 2 was generated using the same filtering as dataset 1 but SNPs linked within each RAD locus were avoided by only retaining one SNP at random per locus, required for population structure analyses (Pritchard et al., 2000). Finally, for dataset 3 (*within populations*), which was used to calculate genetic distance between populations, individuals were assigned to their population of origin and loci were retained if present in 50% of the populations. This filtering was designed to reduce the inclusion of duplicate loci and balance the amount of missing data with the number of informative loci (Andrews et al., 2016). A minimum stack depth of five is higher than the default of two, but within the recommended range (Paris et al., 2017), and helps to remove potential paralogues. Spurious SNPs were avoided by using a minor allele frequency of >0.01 (Marandel et al., 2020), and the combination of a maximum observed heterozygosity of 0.7 (70% of the individuals or populations can be heterozygous for each locus), which are present in either 60% of individuals (datasets 1 and 2) or 50% of the populations (dataset 3), retains loci that have been successfully genotyped across individuals, but are not completely heterozygous. The summary statistics for each population were calculated in Stacks during the filtering of dataset 3 and included the following: the number of private alleles (PRI), expected heterozygosity (*H*_E_), observed heterozygosity (*H*_O_), percentage of polymorphic loci (%; Table 3), the inbreeding coefficient (*F*_IS_), and nucleotide diversity (π; Supplementary information).

**Table 3:** Summary statistics of within *Brassica oleracea* population genetic diversity based on both variant nucleotide sites alone (var) and all sites (all) from dataset 1, showing: the number of individuals (N), the number of private alleles (PRI), expected heterozygosity (*H*_E_), observed heterozygosity (*H*_O_), and percentage of polymorphic loci (%)

Region^a^	Population	*N*	PRI	*H*_E_		*H*_O_		%
				*Var*	*All*	*Var*	*All*	*All*
ES	Auchmithie	4	1,683	0.1043	0.0012	0.1202	0.0014	33.0
ES	Crail	4	1,727	0.1327	0.0019	0.1267	0.0018	52.8
ES	Fortrose	3	12,951	0.2006	0.0032	0.1962	0.0031	76.4
WS	Kildonan Castle	4	1,014	0.0903	0.0014	0.0944	0.0014	40.8
NEE	Tynemouth	4	1,476	0.1023	0.0013	0.0881	0.0011	36.4
NEE	Whitby	4	1,573	0.1200	0.0020	0.1184	0.0020	56.7
SW	Tenby	4	1,568	0.1227	0.0014	0.1153	0.0013	40.5
SW	Llantwit Major	4	2,073	0.1390	0.0023	0.1231	0.0022	66.2
SWE	Prussia Cove	4	1,454	0.1019	0.0016	0.1064	0.0017	45.5
SWE	Fowey	4	1,137	0.1126	0.0018	0.1083	0.0017	53.4
SWE	West Looe	2	1,412	0.1150	0.0011	0.1328	0.0013	27.1
SWE	St. Aldhelm's Head	4	2,470	0.1486	0.0014	0.1676	0.0016	39.4
A	Cudillero	4	716	0.0918	0.0015	0.0938	0.0016	44.3
A	Playa de Xágo	4	1,583	0.1140	0.0012	0.1191	0.0012	33.4
A	Cabo de Peñas	4	698	0.0933	0.0015	0.0910	0.0014	42.5
A	Playas de Viodo	4	503	0.0545	0.0004	0.0580	0.0004	11.2
C	Playa Pedrero	4	1,741	0.1313	0.0014	0.1408	0.0015	38.5
BC	San Juan de Gaztelugatxe	4	2,608	0.1423	0.0012	0.1471	0.0012	34.0
BC	Getaría	4	1,550	0.1280	0.0021	0.1391	0.0023	59.8
BC	San Sebastian	3	2,516	0.1530	0.0023	0.1538	0.0023	61.4

^a^ Region codes: ES, East Scotland; WS, West Scotland; NEE, Northeastern England; SW, South Wales; SWE, Southwestern England; A, Asturias Spain; C, Cantabrica Spain; BC, Basque Country Spain.

### 2.3 Data analyses

Clustering of samples within and between populations was investigated with dataset 1 using RAxML (v8.2; GTRCAT model and 1,000 maximum‐likelihood bootstrap replicates; Stamatakis, 2014) and visualization in SplitsTree4 (Huson & Bryant, 2005). To estimate the number of putative genetic clusters (K) and assess shared genetic ancestry, STRUCTURE (v2.3.4; Pritchard et al., 2000) was used with dataset 2, so as not to inflate sharing based on multiple SNPs within a RAD locus. A range of *K* values were tested (the number of populations successfully sequenced plus one; 1–21) using an admixture model that assumed correlated allele frequencies. For each K, five independent replicates of 100,000 MCMC repetitions, after a burn‐in of 10,000 iterations, were run. The most likely *K* was selected using the log‐likelihoods and deltaK (Evanno et al., 2005). To see whether there were significant differences between estimates of *H*_E_ and *H*_O_, pairwise ANOVAs were carried out in R version 3.4.0 (R Core Team, 2017) on estimates from dataset 3 based on variant sites alone and all sites. A genetic distance matrix was created using dataset 3, and the latitude and longitude of each population were used to calculate a geographic distance matrix using "Haversine" Great Circle Distance in the R package "geosphere" (Hijmans, 2017). In addition, genetic and geographic matrices were created for Spanish and UK populations separately, alongside a temporal distance matrix for the year when each population was first recorded within the UK (first population record; Table 2). Mantel tests were carried out with 9,999 replicates on the region‐wide matrices and country matrices separately, to assess both the overall and within‐country isolation by distance. Mantel tests were also carried out on the UK‐specific matrices to investigate any relationship between the first population records and the genetic and geographic distances.

A subset of dataset 1 where the soil pH was known was used to investigate the relationship between soil pH and $H_{E}$—for example, is a higher soil pH associated with lower genetic diversity? A linear model with soil pH as a predictor variable and *H*_E_ as a response variable was run on 21 individuals (across six populations) from four soil pH classes: Neutral (6.6–7.3), Slightly acidic (6.1–6.5), Moderately acidic (5.6–6.0), and Strongly acidic (5.0–5.5) based on USDA (1998).

In order to identify potential genotype–environment associations, redundancy analyses (RDA) were carried out using dataset 1 following Forester et al. (2018) with the R packages "vegan" and "pysch" (Oksanen et al., 2017; Revelle, 2018). The climate dataset was downloaded from the WorldClim database at a resolution of 4.5 km (Fick & Hijmans, 2017). This dataset is based on measurements made between 1970 and 2000. Therefore, it is assumed that any changes in climate will be consistent enough across the study gradient to maintain differences in the averages and variation between populations. The 19 climate variables available from WorldClim for our dataset were checked for pairwise correlations and the estimated variance inflation factor (VIF). Variables with correlations > |0.7| and VIF > 10 were removed, leaving: "Annual Mean Temperature," "Mean Temperature of Wettest Quarter," "Annual Precipitation," and "Precipitation Seasonality." Longitude was included as an additional predictor variable because it was weakly correlated with climatic variables. Those SNPs that had RDA loadings with *q*‐values < 0.1 were considered outlier loci and were compared to the annotated *B. oleracea* genome using Bedtools (v.2.17.0; Quinlan & Hall, 2010), followed by a search of the online resource "Bolbase" (Yu et al., 2013) to investigate putative gene functions.

## 3 RESULTS

### 3.1 Patterns of genetic diversity

A total of 115,746,909 reads from 76 individuals (20 populations; Table 2) were of sufficient quality and retained for downstream analysis (average reads per individual: 1,522,986; range: 220,363–5,361,799; Table S1). For four of the populations, no individuals were successfully sequenced and so these were not included in these analyses. On average, 86.3% (range 82.5–88.6) of reads mapped to the reference genome (Figure S1). Datasets 1 and 2 contained 42,517 and 13,352 SNPs, respectively, across 13,352 RAD loci (Table S2). There were 140,131 SNPs across 53,539 RAD loci in dataset 3 (Supplementary Information).

Based on variable nucleotide sites only (Table 3), average estimates of genetic diversity (considering *H*_E_) were lower than in the studies cited in Table 1; the average across populations was 0.120 among both UK (range 0.090–0.200) and Spanish (range 0.055–0.153) populations. Observed heterozygosity was consistently significantly (*H*_O_ *p* < 0.001) greater than *H*_E_ for all populations, and average *F*_IS_ was similar in the two geographic regions (UK: average = 0.039, range = 0.001 to 0.084; Spain: average = 0.027, range = 0.025–0.031). There was thus no evidence of inbreeding (as expected given the genetically controlled self‐incompatibility system) but heterozygosity excess was apparent in all populations. The Fortrose population contained 10‐fold more private alleles compared with all other populations and had the highest values for both *H*_E_ and *H*_O_. Values considering all sites were lower but did not change conclusions about relative patterns of diversity (Table 3).

### 3.2 Population structure

Based on the RAxML tree, the majority of individuals clustered by population, with the exceptions of: (a) two individuals that did not cluster with any population (one in San Juan de Gaztelugatxe, Spain, and one in St Aldhelm's Head, UK), and (b) an individual from Fortrose (Scotland, UK) that clustered more closely with other Scottish populations than other individuals from Fortrose (Figure 1ii). The most likely number of genetic clusters from STRUCTURE analyses was *K* = 12. Most individuals were admixed; however, six of the UK populations (Fortrose, Auchmithie, Crail, Tynemouth, Whitby, and Llantwit Major) were dominated by a single genetic ancestry, and two individuals from Fortrose were distinct from both the third individual from Fortrose and all other samples (Figure 1iii). The dominant genetic ancestry seen in individuals from Whitby (UK) also dominated the genetic ancestry of individuals from Cabo de Peñas (Spain), and similarly, the dominant genetic ancestry seen in individuals from Tenby (UK) was most prevalent in individuals from San Juan de Gaztelugatxe and Getaría (Spain). There were three potential regional clusters indicated by the RAxML tree and STRUCTURE analysis: (a) populations in Scotland; (b) populations closest to the Welsh‐English border; and (c) populations in the Basque Country, Spain (excluding San Sebastian; Figure 1). However, the clustering of populations was not well resolved and these "regional clusters" were not always the most geographically close populations (e.g., in cluster c, San Sebastian is closer to Getaría than San Juan de Gaztelugatxegeographically but not genetically). No isolation by distance was predicted by the data either region‐wide, or within Spanish or UK populations alone (Mantel test *p*‐values = 0.474, 0.658 and 0.705, respectively). Furthermore, no relationship was found between the first record for each of UK populations (Table 2) with either geographic or genetic distance (Mantel test *p*‐values = 0.114 and 0.933, respectively).

### 3.3 Environmental associations

**Figure 2:**
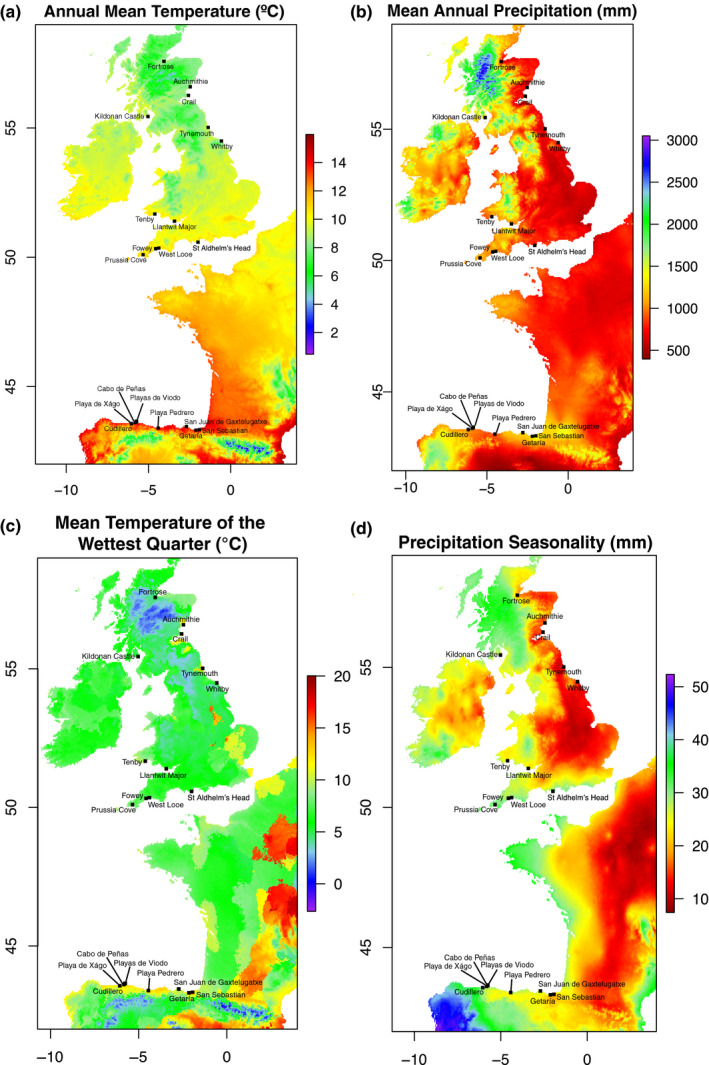
The distribution of sampled populations in relation to various climate variables: (a) annual mean temperature (°C); (b) mean annual precipitation (mm); (c) mean temperature of wettest quarter (°C); and (d) precipitation seasonality (mm). These are averages between 1970 and 2000 obtained from the WorldClim database (Fick & Hijmans, 2017)

**Figure 3:**
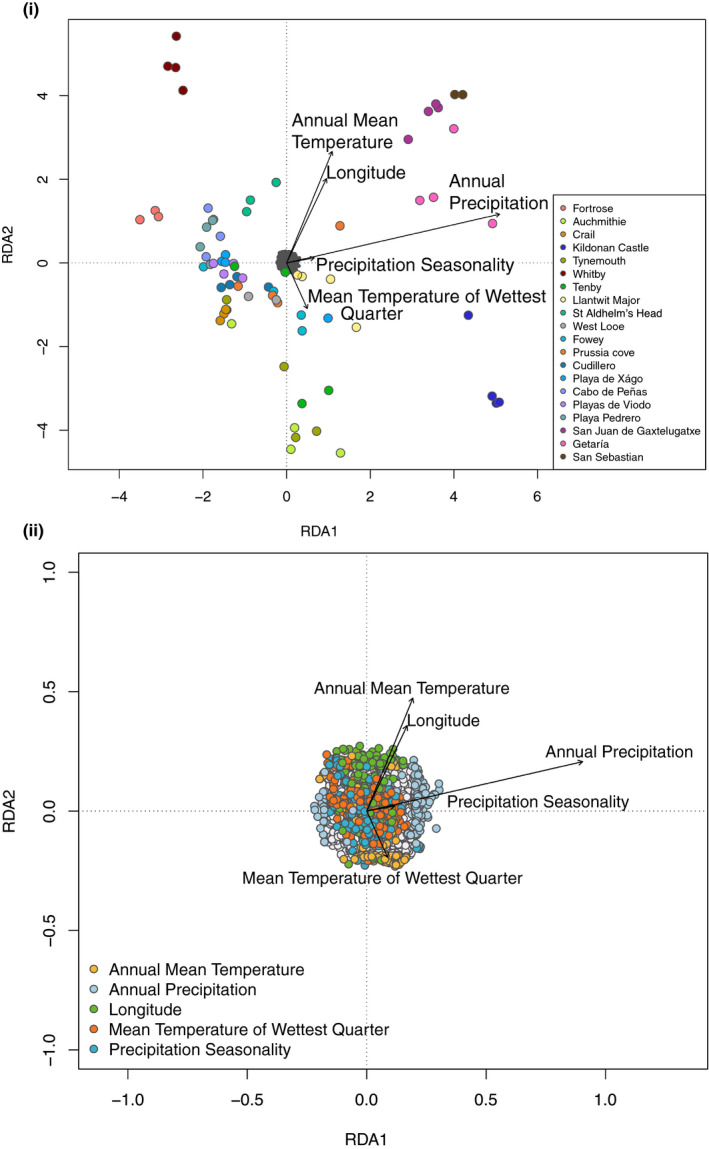
(i) Redundancy analysis (RDA) ordination plot of the association between *Brassica oleracea* individuals (colored points) and SNPs (dark gray points), with environmental variables. The different colors indicate which population each individual was from. (ii) RDA ordination plot of the SNPs alone, colored for the environmental variable with which they were most strongly associated. For both (i) & (ii), the arrows indicate the environmental predictors and the strength of the association

**Figure 4:**
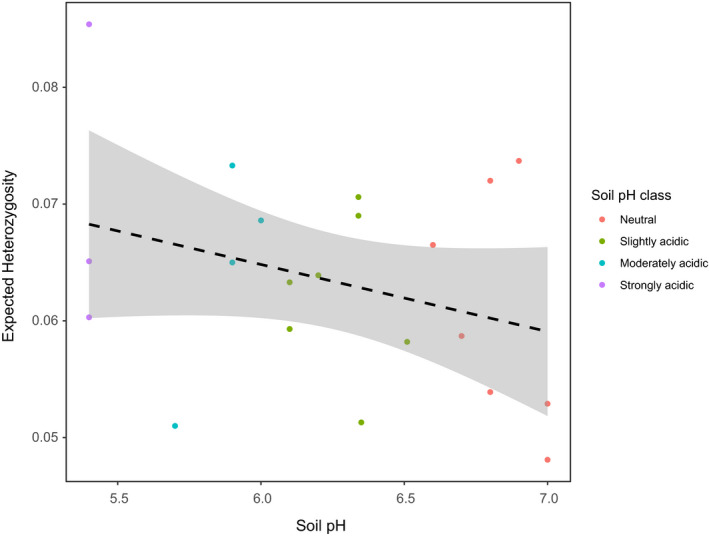
The relationship between expected heterozygosity and soil pH for 21 individuals from four soil pH classes categorized into: Neutral (6.6–7.3), Slightly acidic (6.1–6.5), Moderately acidic (5.6–6.0), and Strongly acidic (5.0–5.5) based on USDA (1998). A linear model was used to fit a regression line (dashed black line), and the standard error is shown in gray, *p*‐value > 0.05

Overall, environmental variables explained 2.3% (adjusted r‐squared) of the variation in the SNPs using RDA analysis; the strongest association of genotype with the environment was with annual precipitation (Figure 3). This environmental variation was strong enough to be reflected in the clustering of individuals, including the genetically distinct individuals from Fortrose (UK; Figure 1iii). For example, across regions, west Scotland and the Basque country experienced the greatest amount of annual precipitation on average (Figure 2b), whereas the annual mean temperature was greater in the Basque country compared with west Scotland (Figure 2a). Individuals from populations in these regions separated from other populations in the same direction as annual precipitation, but in opposing directions in relation to annual mean temperature (Figure 3i). Individuals from Whitby (UK) appear to have experienced a colder, drier environment than the geographically closest population, Tynemouth (UK), which was also reflected in the RDA analysis. Linear modeling indicated a nonsignificant negative trend between genetic diversity (*H*_E_, *H*_O_, and π) and soil pH (i.e., plant genetic diversity decreased as soil pH increased. Only $H_{E}$ is shown, but the same relationship was found with *H*_O_ and π; Figure 4).

There were 2,249 unique candidate SNPs associated with the predictor variables from the RDA analysis; the majority of these (1,039) were most closely associated with "Mean Temperature of Wettest Quarter", followed by "Precipitation Seasonality" (349), "Longitude" (333), "Annual Precipitation" (269), and "Annual Mean Temperature" (259). These were fairly evenly distributed across the genome with no indications of any single SNP with a large effect (Figure S1). A few SNPs that were more closely associated with annual precipitation had strong loadings along axis 1 in the direction of the annual precipitation vector (Figure 3ii). In total, 221 candidate SNPs mapped to unique genes in the *B. oleracea* reference genome, and of the top 18, six were annotated as part of the receptor‐like kinase family (Table 4).

**Table 4:** The top 18 candidate SNPs that mapped to unique genes in the *Brassica oleracea* reference genome and their annotations from "Bolbase" (Yu et al., 2013)

Chromosome	Location	Identity	X	Bolbase gene name	Potential protein	Function
C09	32,879,582	1	−	Bol019890	Ribonucleotide reductase‐related	Fatty acid metabolic process, creation of DNA from RNA
C04	39,737,611	0.999979	−	Bol021601	Unknown	
C09	8,499,546	1	+	Bol032146	Basic helix‐loop‐helix dimerization region	Nucleus transcription regulation
C07	43,014,116	1	−	Bol042101	Toll‐Interleukin receptor	Signal transduction, immune response, disease resistance
C02	233,586	1	+	Bol012817	Laccase/multicopper oxidase	Copper ion binding, metabolic process, maybe formation and degradation of lignin
C04	22,051,514	0.999656	+	Bol044300	Protein kinase‐serine/threonine	Protein kinase activity, signaling, plant defense
C03	29,308,196	0.472347	−	Bol012462	PIK‐related kinase	Binding and DNA repair
C03	48,963,472	0.99438	+	Bol029900	Protein kinase	Protein kinase activity, signaling, plant defense
C04	28,456,859	0.999661	−	Bol009961	Cystathionine beta‐synthase	Vitamin B6 pathway?
C03	9,456,274	1	−	Bol005573	Unknown	
C05	2,317,477	0.580051	−	Bol041075	Pentatricopeptide repeat	Often essential in mitochondria
C04	35,972,614	0.304057	+	Bol037830	Bacterial transferase hexapeptide repeat	Binding and transferase activity
C04	35,104,965	0.996501	+	Bol037950	Cyclin‐like F‐box	Growth and development
C03	2,461,137	0.999261	−	Bol034275	Serine/threonine‐protein kinase	Signaling, plant defense
C02	233,586	0.168963	−	Bol012816	Serine/threonine‐protein kinase	Signaling, plant defense
C01	11,164,295	0.999978	+	Bol039465	Initiation factor eIF−4 gamma, MA3	
C01	11,431,159	1	+	Bol039505	Heat‐shock protein Hsp20	
C01	12,106,862	0.918256	−	Bol039585	F‐box associated	

## 4 DISCUSSION

The results presented here provide the first genome‐wide estimates of genetic variation and population genetic structure of wild cabbages collected from across the UK and Spain. Although direct comparisons with cultivated species would be required to rigorously test hypotheses about origins of these populations, patterns of variation are consistent with recent linguistic and historical evidence (Maggioni, 2015; Maggioni et al., 2018), suggesting that the domestication of *B. oleracea* crops occurred in the Mediterranean, domesticates were moved by people across Europe, escaped, and established wild populations in the Atlantic region. For example, there was no indication of isolation by distance from northern Scotland to Spain (>14˚ latitude), which might be expected if these plants were natural colonizers following common phylogeographic patterns (e.g., Sharbel et al., 2000). Furthermore, genetic ancestry and clustering analyses suggested that geographically distant populations may have similar genetic sources, and could therefore have been established by similar source cultivars. The consistent excess of heterozygotes across populations, combined with evidence for admixture from STRUCTURE analyses, suggests mixing between "isolated" populations (Rousset & Raymond, 1995), which could be due to interbreeding between cultivated plants growing near the wild populations. This highlights the possibility of continued introgression between cultivated and wild plants. Despite the lack of geographic genetic population structuring, there were signals of local adaptation to different climates based on RDA analyses. In addition, within‐population genetic diversity estimates were comparable to other studies (e.g., Christensen et al., 2011; Watson‐Jones et al., 2006), and, as Watson‐Jones et al. (2006) found, lower genetic diversity estimates were associated with higher soil pH. Therefore, these wild populations could hold useful adaptive alleles for plant breeding, and a suitable approach to investigate traits of agricultural interest (e.g., drought tolerance) could be to choose populations based on their environment of origin. However, further sequencing of a range of cultivars from different geographic regions would be required to further test these hypotheses.

### 4.1 Patterns of genetic diversity

Although the magnitude of estimates of genetic diversity based on the ddRADseq data presented here were lower than in previous studies (see Table 1) using allozymes (Lanner‐Herrera et al., 1996; Lázaro & Aguinagalde, 1998; Raybould et al., 1999), microsatellites (Raybould et al., 1999), or AFLPs (Christensen et al., 2011; Watson‐Jones et al., 2006), patterns of variation within the UK and Spain were strikingly similar to one another. Most populations also showed a relatively consistent excess of heterozygosity. These similarities could provide evidence for relatively recent origins of populations in the two regions, but whether this was from feralization of cultivars or natural differentiation after natural colonization cannot be distinguished by the data. Although there has been an ongoing debate as to the origin of wild *B. oleracea* populations in the Atlantic region (Allender et al., 2007; Maggioni, 2015; Song et al., 1990), domestication of *B. oleracea* in the Mediterranean region has been suggested by other genetic, phenotypic, and linguistic studies (Maggioni, 2015; Maggioni et al., 2018; Mitchell, 1976). The subsequent movement of *B. oleracea* cultivars across Europe could then have resulted in a much narrower bottleneck than the initial domestication bottleneck in the Mediterranean as it removed the chance of gene flow from the wild relatives they originated from (Kofsky et al., 2018). Consistent with this hypothesis, although the putative Mediterranean progenitor species remains unknown, Allender et al. (2007) found much greater estimates of genetic diversity within potential progenitor species from the Mediterranean region than either previous genetic diversity estimates made in *B. oleracea* (e.g., Christensen et al., 2011; Watson‐Jones et al., 2006) or in this study.

### 4.2 Population structure

Several of the analyses here suggest less population structuring than might be expected in such geographically distinct populations if natural range expansion followed by isolation occurred. In this dataset, since the first recorded population (Tenby in 1773), one to three new populations have been recorded every thirty years within the UK (Table 2). However, neither the date the UK populations were first recorded, nor the genetic distances between populations in the UK and Spain, had a geographical pattern (i.e., no isolation by distance). Furthermore, although the majority of individuals clustered by population and some regional clustering was seen (Figure 1), it would not be possible to predict whether two individuals from geographically close or geographically distant populations are more genetically similar to each other. For example, Fowey and Prussia Cove (UK populations), and West Looe and Cabo de Peñas (UK and Spanish populations respectively), clustered together and shared more genetic ancestry than Fowey and West Looe, which are the closest geographically. Although more sampling would be required to explicitly test it, the evidence here suggests that these plants have not colonized the Atlantic region following common phylogeographic patterns (e.g., Sharbel et al., 2000) and therefore is consistent with *B. oleracea* domestication occurring outside of the Atlantic region. This is in line with results from other genetic, phenotypic, and linguistic studies, which suggest the Mediterranean region is the most likely location for *B. oleracea* domestication (Maggioni, 2015; Maggioni et al., 2018; Mitchell, 1976).

The genetic ancestry and clustering analyses hint that populations could have been established by escapees from different cultivars. The majority of individuals were assigned to multiple sources of genetic ancestry (Figure 1iii); however, there were also cases where one putative source dominated at the individual‐ and population‐levels, which could be the overall genetic background from the original source cultivar. Interestingly, there were two distinct individuals from Fortrose (10‐fold more private alleles than other populations; Table 3) with a source that was assigned to no other individuals. Due to the ease of interbreeding between cultivars (Allender et al., 2007), this could indicate that these two Fortrose individuals are recent escapees from a different source population (e.g., local gardens), which are yet to have mixed with other individuals within the population. Furthermore, the more recent record of the population at Fortrose (1968), and the lack of assignment to other populations, suggests that this genetic background could be from a cultivar that has not been grown for a long period of time or widely around the Atlantic coastlines. The excess of heterozygotes (*H*_O_ was significantly greater than *H*_E_) and the general mix of shared genetic ancestry across such a wide geographical area in distinct populations could also be an indication of continued introgression into these wild populations from agricultural and horticultural sources. It would be interesting to identify popular cultivars in the local areas of these populations, including any changes in the preferred cultivars through time, to investigate patterns of introgression in more detail. Such direct comparisons with cultivars could identify the most likely founder of these populations.

Using chloroplast microsatellite DNA markers, Allender et al. (2007) found two haplotypes in *B. oleracea* around the coasts of the UK; out of sixteen populations, fourteen were C:01 and two were C:04. The two populations with the C:04 haplotype were in Tyne & Wear, in the northeast of England; in the current study, this area is represented by the Tynemouth and Whitby populations. In line with the rarity of the chloroplast haplotypes identified in this region in the previous study, these two populations clustered most closely with populations not sampled by Allender et al. (2007); Tynemouth clustered with Fortrose, Scotland, and Whitby with the Spanish population Cabo de Peñas. Based on this information, it might be expected that the chloroplast haplotypes of Fortrose and Cabo de Peñas would also be C:04. In addition, the C:01 haplotype found in the majority of the UK populations was also found in four other species of *Brassica* (Allender et al., 2007), suggesting either that this is the ancestral form or introgression between species. A combination of nuclear and chloroplast information could be useful for disentangling the population histories further, particularly in relation to identifying introgression.

Knowledge of the founding cultivars would be useful for both plant breeders and those interested in invasive species. It could provide insights into how different cultivars have adapted (and therefore may adapt in the future) to different environmental conditions, and could also be thought of as a way to compare invasion success within a species. *Brassica oleracea* lack the characteristics thought to be fundamental for establishment in novel locations (invasions; Funk et al., 2016), but perhaps among the huge phenotypic variation found within this species, some traits are more likely to lead to successful "invasions" of particular cultivars compared to others. For example, a cultivated Danish kale was the most likely source for a wild population found in Denmark (based on AFLP markers; Christensen et al., 2011), and it could be that all the Atlantic populations were established by different kale cultivars. Overall, populations of *B. oleracea* growing along Atlantic coasts would be an excellent study system to improve understanding of invasive species that are likely to harbor useful adaptive traits for agriculture.

While comparisons with published whole‐genome sequence data or other types of genotype by sequencing approaches (e.g., Stansell et al., 2018) for cultivated *B. oleracea* would be interesting to more explicitly test origins of the populations studied here, there are several issues with ddRAD data that would make this challenging and potentially hard to interpret. A benefit of ddRAD sequencing is the generation of discrete loci that are standardized to the same length. However, the resulting short sequence segments normally contain only one or a few SNPs, which does not allow accurate assignment of paralogs in highly duplicated and rearranged genomes such as found in the Brassicaceae (e.g., Schranz et al., 2006). Instead, filtering pipelines to allow population genetics analyses based on ddRAD data are designed to be conservative (Marandel et al., 2020; Paris et al., 2017). This filtering results in fewer loci retained, but it should reduce risks of including duplicates. In the current study, excess heterozygosity was observed consistently across populations, which could suggest historical introgression. Although we cannot completely rule out the influence of combining duplicates (Ilut et al., 2014), the highly consistent patterns of excess suggest that all populations would have been affected similarly, enabling interpretations of relative variation within and between populations. The admixture suggested by the STRUCTURE analyses also supports the role of introgression in the histories of the studied populations. However, mapping of the ddRAD reads to multiple reference genomes or to data generated based on different restriction enzymes would be more problematic.

### 4.3 Environmental associations

Despite the general lack of geographic clustering, there was evidence of local adaptation to the varying environments using redundancy analyses, particularly to annual precipitation (Figure 3). Although Watson‐Jones et al. (2006) found some population structuring within the UK, the same result was not found in this study (i.e., no isolation by distance within the UK). Furthermore, no evidence of population structuring was found in the Spanish populations here, and Maggioni *et al*. (personal communication) found no evidence of population structuring in French Atlantic populations. These results could also be correlated with annual precipitation; perhaps the strong variation in annual precipitation in the UK (e.g., a strong west–east gradient) is causing more differentiation between these populations, whereas along the French range annual precipitation has a smaller gradient. One reason for the importance of annual precipitation other than water availability could be the influence of precipitation on soil pH. Soil pH is primarily determined by bedrock, but is also altered by precipitation through leaching of compounds such as calcium carbonate (Kinzel, 1983). Therefore, although slightly alkaline to neutral soils tend to form over limestone, secondary acidification can occur under higher precipitation regimes. The soil pH values recorded here ranged from neutral to strongly acidic (Figure 4). Furthermore, the bedrock of a large proportion of the populations used here (Table 2) differs from the limestone and chalk cliffs that wild *B. oleracea* are thought to be predominantly found on Christensen et al. (2011). For those individuals where the soil pH was known, the same trend was found here as by Watson‐Jones et al. (2006), with a decrease in plant genetic diversity as soil pH increased (Figure 4). For agriculture and horticulture, soil pH is an important consideration (Tilman et al., 2011). The change in plant genetic diversity suggests that soil pH is a strong selective pressure in the wild, causing an adaptive ecological bottleneck in locations where it is higher, resulting in lower genetic diversity. These indications of local adaptation despite a lack of population structure highlight environmental variables that could be investigated further in wild populations of *B. oleracea*, which regardless of their origin are surviving.

Alongside survival, a huge concern for food security related to climate change is the ability of crop plants to remain productive under rapidly changing environmental conditions (Lasky et al., 2015). Obtaining accurate phenotypic data for adaptive traits is a major barrier as we often do not know the combination of traits that underlie differences in fitness or how these vary with the environment (Kooyers et al., 2015). Although some traits will be locally adaptive due to large effect loci, the vast majority of adaptive traits are likely to have a polygenic basis (Rockman, 2012), particularly in the case of multitrait phenotypes related to environmental gradients. Our results match these expectations, as no large effect loci were found; however, some were more significantly associated with the assessed environmental variation than others. The most likely assignment for six of the top 18 candidate genes was to the receptor‐like kinase family (Table 4). This gene family underwent an expansion that is believed to be a plant‐specific adaptation for pathogen defense (Afzal et al., 2008). Interestingly, Zhang et al. (2014) also found differences in genes related to plant defense when investigating adaptations of rice (*Oryza* sp.) across four continents. These results highlight the fundamental importance of the immune system to fitness and suggest that it could be related to environmental differences across different spatial scales. Given that immune system genes are among the best candidates for local adaptation, there is a potential connection between plant genetic diversity, soil pH, and pathogens. It would be interesting to investigate whether less acidic soils host more pathogens, increasing the selective pressure on the plants and decreasing the plant genetic diversity in these soils. Overall, the impact of climate change on the spread of virulence of plant pathogens and herbivores, and the phenological mismatches that may occur between interacting species remain unknown (De Lucia et al., 2012; Fisher et al., 2012; Yang & Rudolf, 2010). What is clear is that plant defense will continue to be an important component of crop productivity, warranting further research.

Overall, the results presented here supported the hypothesis that wild populations of *B. oleracea* in the Atlantic region were established by plants from agricultural and/or horticultural sources. In addition, regardless of their origin, these wild populations are likely to contain useful genetic resources and should be considered as valuable populations of a crop wild relative to be investigated further.

## CONFLICT OF INTEREST

None declared.

## AUTHOR CONTRIBUTIONS

**Elizabeth Mittelll:** Conceptualization (lead); Data curation (lead); Formal analysis (lead); Funding acquisition (supporting); Investigation (lead); Methodology (equal); Project administration (supporting); Validation (equal); Visualization (lead); Writing—original draft (lead); and Writing—review and editing (equal). **Christina Cobbold:** Conceptualization (supporting); Funding acquisition (lead); Investigation (supporting); Methodology (equal); Supervision (equal); and Writing—review and editing (equal). **Umer Zeeshan Ijaz:** Data curation (equal); Funding acquisition (lead); Investigation (supporting); Methodology (equal); Supervision (equal); and Writing—review and editing (equal). **Elizabeth Kilbride:** Methodology (supporting); Resources (equal); and Writing—review and editing (supporting). **Karen Moore:** Data curation (lead); Methodology (equal); Validation (equal); and Writing—review and editing (equal). **Barbara Mable:** Conceptualization (lead); Formal analysis (equal); Funding acquisition (lead); Investigation (equal); Methodology (equal); Resources (equal); Supervision (lead); Validation (equal); Visualization (equal); and Writing—review and editing (equal).

## Supporting information

Table S1‐S2‐Fig S1

## Data Availability

The sequencing data and associated meta‐data are available on the European Nucleotide Archive under the study accession number: PRJEB38464 (http://www.ebi.ac.uk/ena/data/view/PRJEB38464).
