# Supplementary material for: Feral populations of Brassica oleracea along Atlantic coasts in western Europe
Source: Ecol Evol. 2020 Sep 24;10(20):11810–25. doi: 10.1002/ece3.6821 (PMC7593181; doi:10.1002/ece3.6821)
Supplement: Supplementary file 1 — Table S1‐S2‐Fig S1 [file ECE3-10-11810-s001.pdf]

Ecology and Evolution

Open Access

Feral populations of Brassica oleracea along Atlantic coasts in western Europe

Journal:	*Ecology and Evolution*
Manuscript ID	ECE-2020-04-00487.R1
Wiley - Manuscript type:	Original Research
Date Submitted by the Author:	23-Jul-2020
Complete List of Authors:	Mittelll, Elizabeth; University of Glasgow, IBAHCM; University of Saint Andrews, Centre for Biodiversity<br>Cobbold, Christina; University of Glasgow, School of Mathematics and Statistics<br>Ijaz, Umer; University of Glasgow, School of Engineering<br>Kilbride, Elizabeth; University of Glasgow, IBAHCM<br>Moore, Karen; University of Exeter, University of Exeter Sequencing Service<br>Mable, Barbara; University of Glasgow, Institute of Biodiversity, Animal Health and Comparative Medicine
Category:	Evolutionary Ecology
Habitat:	Terrestrial
Organism:	Plants
Approach:	Molecular Genetics
Abstract:	There has been growing emphasis on the role that crop wild relatives might play in supporting highly selected agriculturally valuable species in the face of climate change. In species that were domesticated many thousands of years ago, distinguishing wild populations from escaped feral forms can be challenging, but reintroducing variation from either source could supplement current cultivated forms. For economically important cabbages (Brassicaceae: Brassica oleracea), "wild'' populations occur throughout Europe but little is known about their genetic variation or potential as resources for breeding more resilient crop varieties. The main aim of this study was to characterise the population structure of geographically isolated wild cabbage populations along the coasts of the UK and Spain, including the Atlantic range edges. Double-digest restriction-site associated DNA sequencing was used to sample individual cabbage genomes, assess the similarity of plants from 20 populations, and explore environment-genotype associations across varying climatic conditions. Interestingly, there were no indications of isolation-by-distance; several geographically close populations were genetically more distinct from each other than to distant populations. Furthermore, several distant populations shared genetic ancestry, which could indicate that they were established by escapees of similar source cultivars. However, there were signals of local adaptation to different environments, including a possible relationship between genetic diversity

and soil pH. Overall, these results highlight wild cabbages in the Atlantic region as an important genetic resource worthy of further research into their relationship with existing crop varieties.

Note: The following files were submitted by the author for peer review, but cannot be converted to PDF. You must view these files (e.g. movies) online.

dataset_1_sumstats.tsv
dataset_2_sumstats.tsv
dataset_3_sumstats.tsv

SCHOLARONE™
Manuscripts

Title: Feral populations of *Brassica oleracea* along Atlantic coasts in western Europe

Running title: Feral *Brassica oleracea* in western Europe

Elizabeth A. Mittell[1,2,*], Christina A. Cobbold[3,4], Umer Zeeshan Ijaz[5], Elizabeth A. Kilbride[1], Karen A. Moore[6] & Barbara K. Mable[1,4]†

[1]Institute of Biodiversity, Animal Health and Comparative Medicine, University of Glasgow, UK; [2]School of Biology, University of St Andrews, UK; [3]School of Mathematics and Statistics, University of Glasgow, UK; [4]The Boyd Orr Centre for Population and Ecosystem Health, University of Glasgow, Glasgow, UK; [5]School of Engineering, University of Glasgow, UK; [6]Exeter Sequencing Service, University of Exeter, UK

Corresponding authors: Elizabeth A. Mittell, *em294@st-andrews.ac.uk, e.mittell@gmail.com & Barbara K. Mable, † Barbara.Mable@glasgow.ac.uk

For Review Only

Abstract

There has been growing emphasis on the role that crop wild relatives might play in supporting highly selected agriculturally valuable species in the face of climate change. In species that were domesticated many thousands of years ago, distinguishing wild populations from escaped feral forms can be challenging, but reintroducing variation from either source could supplement current cultivated forms. For economically important cabbages (Brassicaceae: *Brassica oleracea*), "wild" populations occur throughout Europe but little is known about their genetic variation or potential as resources for breeding more resilient crop varieties. The main aim of this study was to characterise the population structure of geographically isolated wild cabbage populations along the coasts of the UK and Spain, including the Atlantic range edges. Double-digest restriction-site associated DNA sequencing was used to sample individual cabbage genomes, assess the similarity of plants from 20 populations, and explore environment-genotype associations across varying climatic conditions. Interestingly, there were no indications of isolation-by-distance; several geographically close populations were genetically more

distinct from each other than to distant populations. Furthermore, several distant populations shared genetic ancestry, which could indicate that they were established by escapees of similar source cultivars. However, there were signals of local adaptation to different environments, including a possible relationship between genetic diversity and soil pH. Overall, these results highlight wild cabbages in the Atlantic region as an important genetic resource worthy of further research into their relationship with existing crop varieties.

Keywords: *Brassica oleracea*, feral populations, crop wild relatives, isolation-by-distance, environment-genotype associations, domestication

For Review Only

Introduction

Domestication was an important transition within human societies, which allowed the rise of civilisations (Diamond, 2002). Whilst vital for human success, there have been evolutionary consequences for the domesticated organisms. In crop plants, the selection of 'domestication traits' has led to many desired changes in physiological, morphological and life-history traits compared to their wild relatives (Milla, Osborne, Turcotte, & Violle, 2015; Purugganan & Fuller, 2009). However, traits that are correlated with those selected for (directly or indirectly) can also influence phenotypes via pleiotropic effects (Conner, 2002) and linkage disequilibrium (Falconer & Mackay, 1996). These genetic constraints and narrow population bottlenecks can have unintended genetic consequences for crop plants, particularly elite lines that are the result of intense artificial selection; e.g., reduced genetic diversity, increased genetic drift and increased deleterious allele frequencies (Rauf, Teixeira da Silva, Khan, & Naveed, 2010; von Wettberg et al., 2018). It is also likely that crop lines are constrained to some extent by the environment within which they were originally domesticated. Therefore, to continue to utilise crop plants successfully, it is important to understand both the genetic consequences of domestication, and where it occurred.

A classic example of domestication can be found in the commercially valuable species, *Brassica oleracea* (recognised by Darwin, 1859; Walley et al., 2012). This single species contains a huge amount of morphological diversity in cultivated varieties that has been around since at least the 1stCentury (e.g., kale, kohlrabi, broccoli, Brussels sprouts and cauliflower; Maggioni, von Bothmer, Poulsen, & Lipman, 2018); the same morphological extremes are not found in wild populations. The origin of domesticated

B. oleracea crops and the ‘wild’ or ‘feral’ status of populations, found throughout the UK and along the Atlantic coasts of north-western Europe (Raybould, Mogg, Clarke, Gliddon, & Gray, 1999), has been debated in the literature (Allender, Allainguillaume, Lynn, & King, 2007; Gómez-Campo & Prakash, 1999; Maggioni, 2015; Mitchell, 1976). Initially it was thought that different cultivars were independently domesticated from wild populations on European Atlantic coasts (e.g., Spanish cabbage varieties were domesticated from local wild Spanish populations; Gómez-Campo & Prakash, 1999), and that early domesticates were introduced to and diversified within the Mediterranean region around 3,000 to 4,000 years ago (Allender et al., 2007). Information was limited when this hypothesis was favoured (Allender et al., 2007; Gómez-Campo & Prakash, 1999), although there was already conflicting evidence (Mitchell, 1976). For example, Mitchell (1976) found that the locations of ancient human settlements and modern *B. oleracea* populations coincided along UK coasts, providing a potential source of escapees from domestic settings (agriculture or gardens) that could have established feral populations. This alternative hypothesis that *B. oleracea* originated elsewhere and escaped into the wild in the Atlantic region has been supported by recent linguistic and historical research (Maggioni, 2015; Maggioni et al., 2018). Maggioni (2015) suggested that the most plausible hypothesis is that *B. oleracea* was domesticated in the Mediterranean region, before being moved across Europe by people, where escaped plants established now naturalised populations. However, the genetic status of *B. oleracea* in the Atlantic region is still an open question (*B. oleracea* is classified as a native species in the UK and an alien species in Spain; *Euro+Med PlantBase*, 2020).

For Review Only

The ease with which cultivated and wild *B. oleracea* plants can introgress is an issue for interpreting variation within the *B. oleracea* species complex, as past hybridisation can obscure phylogeographic signals (Allender et al., 2007). However, for crop breeding purposes a close genetic relationship between wild populations and domesticated cultivars may be seen as an advantage; higher genetic similarity could make it easier to introgress adaptive traits from the wild into cultivated varieties (Hoisington et al., 1999). An alternative view is that if these populations are feral they would have experienced the same domestication bottleneck as many cultivars (von Wettberg et al., 2018), and therefore they may not be the important reservoirs of genetic diversity that crop wild relatives are typically assumed to be. Compared to domestication, feralization is under-investigated; however modern genomic data are al-

lowing its occurrence to be identified and consequences better understood (see examples in Henriksen, Gering, & Wright, 2018). Despite the agricultural importance of *B. oleracea*, there has not yet been a comprehensive genetic analysis of wild populations in the Atlantic region that would allow assessment of their utility as sources of variation for cultivation.

Escaped plants can be thought of as 'invasive' species, which are defined as those that became established after introduction outside of the biogeographic region within which they evolved (Prentis, Wilson, Dormontt, Richardson, & Lowe, 2008). However, it is not always clear where these 'native' regions are located, as is the case of *B. oleracea*, or why certain species are successful where others are not. Furthermore, wild populations of *B. oleracea* do not have the characteristics that are thought to be important for successful establishment in novel locations (i.e. 'invasive traits'; Funk, Standish, Stock, & Valladares, 2016). For example, wild *B. oleracea* are: perennials rather than annuals, woody rather than herbaceous, relatively slow- rather than fast-growing, and predominantly outcrossing rather than selfing. Self-fertilisation in plants is inhibited by polymorphic self-incompatibility (SI) recognition systems where haplotype blocks encode distinct proteins for pollen-pistil recognition (Charlesworth, Vekemans, Castric, & Glémin, 2005). A strong SI system exists in *B. oleracea* (a single-locus system with over 60 alleles; Raybould et al., 1999), making them predominantly self-incompatible (Kitashiba & Nasrallah, 2014; Walley et al., 2012; Yousef, Mueller, Börner, & Schmid, 2018). Development of self-compatible lines can aid in propagation of cultivated forms (e.g., Xiao et al., 2019), but reduce adaptive potential to changing environmental conditions. Therefore, even if the "wild" populations include escaped forms, retention of a wide range of self-incompatibility alleles could be used to enhance the potential of breeding strategies designed to maintain heterosis.

Currently too little is known about levels of genetic variation and population structure in wild *B. oleracea* populations to fully assess the potential for use of plants from different regions to supplement crop diversity. Population structure and within population genetic diversity are impacted by gene flow, which occurs via pollen and seeds in plants (Scheepens, Frei, Armbruster, & Stöcklin, 2012; Slatkin, 1987). The main pollinators of *B. oleracea* are bees that fly short distances between plants (average 2 m; Raybould et al., 1999). Seed dispersal was previously thought to be limited to approximately 4 m (Watson-Jones, Maxted, & Ford-Lloyd, 2006). However, Wichmann et al. (2009) found that wind can

For Review Only

spread seeds up to 250 m, and that rare-long distance dispersal events of up to 10 km could occur if seeds became attached to people's shoes. Therefore, although gene flow may be limited between geographically close populations leading to high genetic structuring in some instances, in other cases, such as where plants grow close (0 - 4 m) to well used coastal paths, gene flow might be greater than expected. Genetic diversity estimates have been made in some *B. oleracea* populations within the Atlantic region (e.g., Table 1), but the northern edge (Scotland) has not been investigated. A correlation between genetic distance and geographic distance in wild *B. oleracea* populations was found in some studies (Raybould et al., 1999; Sánchez-Yélamo, 2014) but not others (Christensen et al., 2011; Watson-Jones et al., 2006). Interestingly, Watson-Jones et al. (2006) also considered some environmental variables and found that higher soil pH was associated with lower genetic diversity in English and Welsh populations. The inconsistency in previous studies could be due to the varying spatial scales and molecular markers used. However, overall, these results highlight the uncertainty in the status and genetic contents of wild *B. oleracea* populations in the Atlantic region, as well as the potential effect of environment on the plant genetics. Filling these knowledge gaps could provide important insights into these crop wild relatives for agricultural use.

For Review Only

Brassica oleracea is a good model for investigating the genetic resources available (e.g., the extent of genetic diversity and local adaptation) in a potentially feral crop wild relative because it is diploid and a reference genome is available (Liu et al., 2014). Therefore, compared to other crop species (e.g., polyploids) genetic analyses are simpler. For many questions whole-genome sequencing is unnecessary (Rockman, 2012) and reduced-representation methods, such as double-digest restriction associated DNA sequencing (ddRADseq), are sufficient to: assess genetic diversity within and between populations (Andrews, Good, Miller, Luikart, & Hohenlohe, 2016); determine population genetic structuring (Gao et al., 2017); and investigate potential associations between genotypes and environmental variables (Forester, Lasky, Wagner, & Urban, 2018). Therefore, ddRADseq is an appropriate method for considering the genetic resources in, and local adaptation of, *B. oleracea* populations across their Atlantic range.

Overall, current knowledge on genetic variation of *B. oleracea* in wild populations is patchy in geographic coverage and based on outdated molecular genetic techniques (Table 1). Therefore, this study

combined modern genetic techniques and the reference genome available for this species to increase the power to detect differences among populations across a broad geographic range. The following questions were addressed: (1) how much genetic variation exists among wild populations of *B. oleracea* in the UK and Spain; (2) how are populations structured in the Atlantic region and how much differentiation exists between isolated populations; and (3) are there signals of local adaptation to the environment? The results provide insights into the utility of *B. oleracea* as a crop wild relative genetic resource for agriculture, as well as shed light on the most likely region of *B. oleracea* domestication.

Materials and Methods

Twenty-four populations of *B. oleracea* were chosen from the UK and Spain to cover both a latitudinal and longitudinal gradient of the Atlantic range for genetic analyses (Figure 1i & Table 2). French populations were not sampled here, but are the focus of a recent genetic analysis by Maggioni *et al.* (personal communication). Leaves were collected from four individual plants from each population for DNA extraction, as has been successfully applied to the study of population structure in wild relatives in the Brassicaceae (Buckley, Holub, Koch, Vergeer, & Mable, 2018). Nazareno, Bemmels, Dick, and Lohmann (2017) found that compared to "traditional" population genetic markers these smaller sample sizes are sufficient for various population statistics when large numbers of SNPs are available. The bedrock for each population was obtained from the British Geological Survey (BGS, 2018) and the Instituto Geológico y Minero de España (IGME, 2018). The first year a written record of a population exists was obtained for the UK populations from the Botanical Society of Britain & Ireland (BSBI, 2018). No equivalent records could be found for the Spanish populations.

Molecular methods

High molecular weight DNA was extracted from the leaves of 96 individuals from 24 populations (Table 2) using DNeasy Plant Mini Kits (QIAGEN, Hilden, Germany) and quantified using a Qubit 2.0 Fluorometer (ThermoFisher Scientific, Waltham, Massachusetts, U.S). Four samples from each population were sent for library preparation and sequencing at University of Exeter Sequencing Service. Double-digest

For Review Only

RADseq libraries were made using a modification of the method in Wu et al. (2016) that allowed NexteraXT indexes (Illumine Corp., USA) to be used for multiplexing samples. In addition, an RYRY spacer was inserted in the adapter 3' of the Illumina sequencing primer annealing site to provide additional complexity at the start of read 1 immediately before the Sac1 sticky end. For each sample 400 ng DNA was fully digested with Sac1 and Mse1 restriction endonucleases and purified using Ampure XP beads. Illumina compatible i5 adapters were designed to ligate to the at the AGCT-3' sticky end left after Sac1 digest, and Illumina compatible i7 adapters were designed to ligate to the 5'-TA overhangs remaining after Mse1 digest. Adapter-ligation excess adapters were removed using Ampure XP beads. DNA fragments were amplified by 12 cycles of indexing PCR, purified, size selected (inserts 330-670 bp) and validated using a Tapestation D1000 HS Screentape (Agilent Technologies Ltd). Libraries were equimolar pooled and the pool concentration was calculated after qPCR. Libraries were denatured, diluted and sequenced with 125bp paired-end reads on Illumina HiSeq 2500 using SBS High Output reagents v4 (Illumina Corp., USA).

Data processing

Reads were demultiplexed and trimmed to 100 bp using cutadapt (Martin, 2011). These were then cleaned and quality filtered using the *process_radtags* pipeline in Stacks v1.47 (Rochette & Catchen, 2017). Bowtie (v2; Langmead & Salzberg, 2012) and samtools (v1.9; Li et al., 2009) were used to align the reads to the *B. oleracea* reference genome (Liu et al., 2014). A catalogue of stacks was then created using *ref_map* (Stacks) with the default settings. The *populations* pipeline (Stacks) was used to filter the data, and calculate summary statistics. Three datasets were generated with different filtering parameters depending on the downstream analysis. Firstly, for dataset 1 (*within individuals*), which was used to estimate genetic diversity within individuals and in phylogenetic analyses, all individuals were filtered as a single population, and loci were retained if they had a minimum individual stack depth of five, a minimum minor allele frequency of 0.01, a maximum observed heterozygosity of 0.7 and were present in 60% of individuals. Secondly, dataset 2 was generated using the same filtering as dataset 1 but SNPs linked within each RAD locus were avoided by only retaining one SNP at random per locus; required for population structure analyses (Pritchard, Stephens, & Donnelly, 2000). Finally, for dataset

For Review Only

3 (*within populations*), which was used to calculate genetic distance between populations, individuals were assigned to their population of origin and loci were retained if present in 50% of the populations. This filtering was designed to reduce the inclusion of duplicate loci and balance the amount of missing data with the number of informative loci (Andrews et al., 2016). A minimum stack depth of five is higher than the default of two, but within the recommended range (Paris, Stevens, & Catchen, 2017), and helps to remove potential paralogues. Spurious SNPs were avoided by using a minor allele frequency of > 0.01 (Marandel et al., 2020), and the combination of a maximum observed heterozygosity of 0.7 (70% of the individuals or populations can be heterozygous for each locus) which are present in either 60% of individuals (datasets 1 and 2) or 50% of the populations (dataset 3) retains loci that have been successfully genotyped across individuals, but are not completely heterozygous. The summary statistics for each population were calculated in Stacks during the filtering of dataset 3 and included: the number of private alleles (PRI), expected heterozygosity (H_E), observed heterozygosity (H_O), percentage of polymorphic loci (%; Table 3), the inbreeding coefficient (F_{IS}) and nucleotide diversity (π; Supplementary information).

Data analyses

Clustering of samples within and between populations was investigated with dataset 1 using RAxML (v8.2; GTRCAT model and 1000 maximum likelihood bootstrap replicates; Stamatakis, 2014) and visualisation in SplitsTree4 (Huson & Bryant, 2005). To estimate the number of putative genetic clusters (K) and assess shared genetic ancestry, STRUCTURE (v2.3.4; Pritchard et al., 2000) was used with dataset 2, so as not to inflate sharing based on multiple SNPs within a RAD locus. A range of K values were tested (the number of populations successfully sequenced plus one; 1 – 21) using an admixture model that assumed correlated allele frequencies. For each K, five independent replicates of 100,000 MCMC repetitions, after a burn-in of 10,000 iterations, were run. The most likely K was selected using the log likelihoods and deltaK (Evanno, Regnaut, & Goudet, 2005). To see if there were significant differences between estimates of H_E and H_O, pairwise-ANOVAs were carried out in R version 3.4.0 (R Core Team, 2017) on estimates from dataset 3 based on variant sites alone and all sites. A genetic distance matrix was created using dataset 3, and the latitude and longitude of each population was

For Review Only

used to calculate a geographic distance matrix using ‘Haversine’ Great Circle Distance in the R package ‘geosphere’ (Hijmans, 2017). In addition, genetic and geographic matrices were created for Spanish and UK populations separately, alongside a temporal distance matrix for the year when each population was first recorded within the UK (first population record; Table 2). Mantel tests were carried out with 9999 replicates on the region-wide matrices and country matrices separately, to assess both the overall and within country isolation-by-distance. Mantel tests were also carried out on the UK specific matrices to investigate any relationship between the first population records and the genetic and geographic distances.

A subset of dataset 1 where the soil pH was known was used to investigate the relationship between soil pH and H_E – e.g., is a higher soil pH associated with lower genetic diversity? A linear model with soil pH as a predictor variable and H_E as a response variable was run on 21 individuals (across six populations) from four soil pH classes: Neutral (6.6 - 7.3), Slightly acidic (6.1 - 6.5), Moderately acidic (5.6 - 6.0) and Strongly acidic (5.0 - 5.5) based on USDA (1998).

In order to identify potential genotype-environment associations, redundancy analyses (RDA) were carried out using dataset 1 following Forester et al. (2018) with the R packages ‘vegan’ and ‘pysch’ (Oksanen et al., 2017; Revelle, 2018). The climate dataset was downloaded from the WorldClim database at a resolution of 4.5 km (Fick & Hijmans, 2017). This dataset is based on measurements made between 1970 – 2000. Therefore, it is assumed that any changes in climate will be consistent enough across the study gradient to maintain differences in the averages and variation between populations. The 19 climate variables available from WorldClim for our dataset were checked for pairwise correlations and the estimated variance inflation factor (VIF). Variables with correlations $> |0.7|$ and VIF > 10 were removed, leaving: ‘Annual Mean Temperature’, ‘Mean Temperature of Wettest Quarter’, ‘Annual Precipitation’ and ‘Precipitation Seasonality’. Longitude was included as an additional predictor variable because it was weakly correlated with climatic variables. Those SNPs that had RDA loadings with q-values < 0.1 were considered outlier loci, and were compared to the annotated *B. oleracea* genome using Bedtools (v.2.17.0; Quinlan & Hall, 2010), followed by a search of the online resource ‘Bolbase’ (Yu et al., 2013) to investigate putative gene functions.

For Review Only

Results

Patterns of genetic diversity

A total of 115,746,909 reads from 76 individuals (20 populations; Table 2) were of sufficient quality and retained for down-stream analysis (average reads per individual: 1,522,986; range: 220,363 – 5,361,799; Supplementary Table 1). For four of the populations, no individuals were successfully sequenced and so these were not included in these analyses. On average 86.3% (range 82.5 - 88.6) of reads mapped to the reference genome (Supplementary Figure 1). Datasets 1 and 2 contained 42,517 and 13,352 SNPs, respectively, across 13,352 RAD-loci (Supplementary Table 2). There were 140,131 SNPs across 53,539 RAD-loci in dataset 3 (Supplementary Information).

Based on variable nucleotide sites only (Table 3), average estimates of genetic diversity (considering H_E) were lower than in the studies cited in Table 1; the average across populations was 0.120 among both UK (range 0.090 – 0.200) and Spanish (range 0.055 – 0.153) populations. Observed heterozygosity was consistently significantly (H_O $p < 0.001$) greater than H_E for all populations and average F_{IS} was similar in the two geographic regions (UK: average = 0.039, range = 0.001 to 0.084; Spain: average = 0.027, range = 0.025 to 0.031). There was thus no evidence of inbreeding (as expected given the genetically controlled self-incompatibility system) but heterozygosity excess was apparent in all populations. The Fortrose population contained 10-fold more private alleles compared to all other populations and had the highest values for both H_E and H_O. Values considering all sites were lower but did not change conclusions about relative patterns of diversity (Table 3).

For Review Only

Population structure

Based on the RAxML tree, the majority of individuals clustered by population, with the exceptions of: (i) two individuals that did not cluster with any population (one in San Juan de Gaxtelugatxe, Spain and one in St Aldehelm's Head, UK), and (ii) an individual from Fortrose (Scotland, UK) that clustered more closely with other Scottish populations than other individuals from Fortrose (Figure 1ii). The most likely number of genetic clusters from STRUCTURE analyses was K = 12. Most individuals were admixed, however, six of the UK populations (Fortrose, Auchmithie, Crail, Tynemouth, Whitby and Llantwit Major)

were dominated by a single genetic ancestry, and two individuals from Fortrose were distinct from both the third individual from Fortrose and all other samples (Figure 1iii). The dominant genetic ancestry seen in individuals from Whitby (UK) also dominated the genetic ancestry of individuals from Cabo de Peñas (Spain), and similarly, the dominant genetic ancestry seen in individuals from Tenby (UK) was most prevalent in individuals from San Juan de Gaxtelugatxe and Getaría (Spain). There were three potential regional clusters indicated by the RAxML tree and STRUCTURE analysis: (a) populations in Scotland; (b) populations closest to the Welsh-English border; and (c) populations in the Basque Country, Spain (excluding San Sebastian; Figure 1). However, the clustering of populations was not well resolved and these 'regional clusters' were not always the most geographically close populations (e.g., in cluster c, San Sebastian is closer to Getaría than San Juan de Gaxtelugaxte geographically but not genetically). No isolation-by-distance was predicted by the data either region-wide, or within Spanish or UK populations alone (Mantel test p-values = 0.474, 0.658 and 0.705, respectively). Furthermore, no relationship was found between the first record for each of UK populations (Table 2) with either geographic or genetic distance (Mantel test p-values = 0.114 and 0.933, respectively).

Environmental associations

Overall, environmental variables explained 2.3% (adjusted r-squared) of the variation in the SNPs using RDA analysis; the strongest association of genotype with the environment was with annual precipitation (Figure 3). This environmental variation was strong enough to be reflected in the clustering of individuals, including the genetically distinct individuals from Fortrose (UK; Figure 1iii). For example, across regions, west Scotland and the Basque country experienced the greatest amount of annual precipitation on average (Figure 2b), whereas the annual mean temperature was greater in the Basque country compared to west Scotland (Figure 2a). Individuals from populations in these regions separated from other populations in the same direction as annual precipitation, but in opposing directions in relation to annual mean temperature (Figure 3i). Individuals from Whitby (UK) appear to have experienced a colder, drier environment than the geographically closest population, Tynemouth (UK), which was also reflected in the RDA analysis. Linear modelling indicated a non-significant negative trend between genetic diversity (H_E, H_O & π) and soil pH (i.e. plant genetic diversity decreased as soil pH increased. Only H_E is shown

For Review Only

but the same relationship was found with H_O & π; Figure 4).

There were 2249 unique candidate SNPs associated with the predictor variables from the RDA analysis; the majority of these (1039) were most closely associated with 'Mean Temperature of Wettest Quarter', followed by 'Precipitation Seasonality' (349), 'Longitude' (333), 'Annual Precipitation' (269) and 'Annual Mean Temperature' (259). These were fairly evenly distributed across the genome with no indications of any single SNP with a large effect. A few SNPs that were more closely associated with annual precipitation had strong loadings along axis 1 in the direction of the annual precipitation vector (Figure 3(ii)). In total, 221 candidate SNPs mapped to unique genes in the *B. oleracea* reference genome, and of the top 18, six were annotated as part of the receptor-like kinase family (Table 4).

Discussion

The results presented here provide the first genome-wide estimates of genetic variation and population genetic structure of wild cabbages collected from across the UK and Spain. Although direct comparisons with cultivated species would be required to rigorously test hypotheses about origins of these populations, patterns of variation are consistent with recent linguistic and historical evidence (Maggioni, 2015; Maggioni et al., 2018) suggesting that the domestication of *B. oleracea* crops occurred in the Mediterranean, domesticates were moved by people across Europe, escaped and established wild populations in the Atlantic region. For example, there was no indication of isolation-by-distance from northern Scotland to Spain ($> 14°$ latitude), which might be expected if these plants were natural colonisers following common phylogeographic patterns (e.g., Sharbel, Haubold, & Mitchell-Olds, 2000). Furthermore, genetic ancestry and clustering analyses suggested that geographically distant populations may have similar genetic sources, and could therefore have been established by similar source cultivars. The consistent excess of heterozygotes across populations, combined with evidence for admixture from STRUCTURE analyses, suggests mixing between 'isolated' populations (Rousset & Raymond, 1995), which could be due to interbreeding between cultivated plants growing near the wild populations. This highlights the possibility of continued introgression between cultivated and wild plants. Despite the lack of geographic genetic population structuring, there were signals of local adaptation to different climates

For Review Only

based on RDA analyses. In addition, within population genetic diversity estimates were comparable to other studies (e.g., Christensen et al., 2011; Watson-Jones et al., 2006), and as Watson-Jones et al. (2006) found, lower genetic diversity estimates were associated with higher soil pH. Therefore, these wild populations could hold useful adaptive alleles for plant breeding, and a suitable approach to investigate traits of agricultural interest (e.g., drought tolerance) could be to choose populations based on their environment of origin. However, further sequencing of a range of cultivars from different geographic regions would be required to further test these hypotheses.

Patterns of Genetic Diversity

For Review Only

Although the magnitude of estimates of genetic diversity based on the ddRADseq data presented here were lower than in previous studies (see Table 1) using allozymes (Lanner-Herrera, Gustafeson, Filt, & Bryngelsson, 1996; Lázaro & Aguinagalde, 1998; Raybould et al., 1999), microsatellites (Raybould et al., 1999) or AFLPs (Watson-Jones et al., 2006; Christensen et al., 2011), patterns of variation within the UK and Spain were strikingly similar to one another. Most populations also showed a relatively consistent excess of heterozygosity. These similarities could provide evidence for relatively recent origins of populations in the two regions, but whether this was from feralisation of cultivars or natural differentiation after natural colonisation cannot be distinguished by the data. Although there has been an ongoing debate as to the origin of wild *B. oleracea* populations in the Atlantic region (Song, Osborn, & Williams, 1990; Allender et al., 2007; Maggioni, 2015), domestication of *B. oleracea* in the Mediterranean region has been suggested by other genetic, phenotypic and linguistic studies (Mitchell, 1976; Maggioni, 2015; Maggioni et al., 2018). The subsequent movement of *B. oleracea* cultivars across Europe could then have resulted in a much narrower bottleneck than the initial domestication bottleneck in the Mediterranean as it removed the chance of gene flow from the wild relatives they originated from (Kofsky, Zhang, & Song, 2018). Consistent with this hypothesis, although the putative Mediterranean progenitor species remains unknown, Allender et al. (2007) found much greater estimates of genetic diversity within potential progenitor species from the Mediterranean region than either previous genetic diversity estimates made in *B. oleracea* (e.g., Christensen et al., 2011; Watson-Jones et al., 2006) or in this study.

Population structure

Several of the analyses here suggest less population structuring than might be expected in such geographically distinct populations if natural range expansion followed by isolation occurred. In this dataset, since the first recorded population (Tenby in 1773), one to three new populations have been recorded every thirty years within the UK (Table 2). However, neither the date the UK populations were first recorded, nor the genetic distances between populations in the UK and Spain, had a geographical pattern (i.e. no isolation by distance). Furthermore, although the majority of individuals clustered by population and some regional clustering was seen (Figure 1), it would not be possible to predict whether two individuals from geographically close or geographically distant populations are more genetically similar to each other. For example, Fowey and Prussia Cove (UK populations), and West Looe and Cabo de Peñas (UK and Spanish populations respectively), clustered together and shared more genetic ancestry than Fowey and West Looe, which are the closest geographically. Although more sampling would be required to explicitly test it, the evidence here suggests that these plants have not colonised the Atlantic region following common phylogeographic patterns (e.g., Sharbel et al., 2000) and therefore is consistent with *B. oleracea* domestication occurring outside of the Atlantic region. This is in line with results from other genetic, phenotypic and linguistic studies, which suggest the Mediterranean region is the most likely location for *B. oleracea* domestication (Maggioni, 2015; Maggioni et al., 2018; Mitchell, 1976).

The genetic ancestry and clustering analyses hint that populations could have been established by escapees from different cultivars. The majority of individuals were assigned to multiple sources of genetic ancestry (Figure 1iii), however, there were also cases where one putative source dominated at the individual- and population-levels, which could be the overall genetic background from the original source cultivar. Interestingly, there were two distinct individuals from Fortrose (10-fold more private alleles than other populations; Table 3) with a source that was assigned to no other individuals. Due to the ease of interbreeding between cultivars (Allender et al., 2007), this could indicate that these two Fortrose individuals are recent escapees from a different source population (e.g., local gardens), which are yet to have mixed with other individuals within the population. Furthermore, the more recent record of the population at Fortrose (1968), and the lack of assignment to other populations, suggests that this genetic background could be from a cultivar that has not been grown for a long period of time or widely

For Review Only

around the Atlantic coastlines. The excess of heterozygotes (H_O was significantly greater than H_E) and the general mix of shared genetic ancestry across such a wide geographical area in distinct populations, could also be an indication of continued introgression into these wild populations from agricultural and horticultural sources. It would be interesting to identify popular cultivars in the local areas of these populations, including any changes in the preferred cultivars through time, to investigate patterns of introgression in more detail. Such direct comparisons with cultivars could identify the most likely founder of these populations.

Using chloroplast microsatellite DNA markers, Allender et al. (2007) found two haplotypes in *B. oleracea* around the coasts of the UK; out of sixteen populations, fourteen were C:01 and two were C:04. The two populations with the C:04 haplotype were in Tyne & Wear, in the northeast of England; in the current study, this area is represented by the Tynemouth and Whitby populations. In line with the rarity of the chloroplast haplotypes identified in this region in the previous study, these two populations clustered most closely with populations not sampled by Allender et al. (2007); Tynemouth clustered with Fortrose, Scotland, and Whitby with the Spanish population Cabo de Peñas. Based on this information, it might be expected that the chloroplast haplotypes of Fortrose and Cabo de Peñas would also be C:04. In addition, the C:01 haplotype found in the majority of the UK populations was also found in four other species of *Brassica* (Allender et al., 2007), suggesting either that this is the ancestral form or introgression between species. A combination of nuclear and chloroplast information could be useful for disentangling the population histories further, particularly in relation to identifying introgression.

Knowledge of the founding cultivars would be useful for both plant breeders and those interested in invasive species. It could provide insights into how different cultivars have adapted (and therefore may adapt in the future) to different environmental conditions, and could also be thought of as a way to compare invasion success within a species. *Brassica oleracea* lack the characteristics thought to be fundamental for establishment in novel locations (invasions; Funk et al., 2016), but perhaps amongst the huge phenotypic variation found within this species, some traits are more likely to lead to successful 'invasions' of particular cultivars compared to others. For example, a cultivated Danish kale was the most likely source for a wild population found in Denmark (based on AFLP markers; Christensen et al., 2011), and it could be that all the Atlantic populations were established by different kale cultivars.

For Review Only

Overall, populations of *B. oleracea* growing along Atlantic coasts would be an excellent study system to improve understanding of invasive species that are likely to harbour useful adaptive traits for agriculture.

While comparisons with published whole genome sequence data or other types of genotype by sequencing approaches (e.g., Stansell et al., 2018) for cultivated *B. oleracea* would be interesting to more explicitly test origins of the populations studied here, there are several issues with ddRAD data that would make this challenging and potentially hard to interpret. A benefit of ddRAD sequencing is the generation of discrete loci that are standardised to the same length. However, the resulting short sequence segments normally contain only one or a few SNPs, which does not allow accurate assignment of paralogs in highly duplicated and rearranged genomes such as found in the Brassicaceae (e.g., Schranz, Lysak, & Mitchell-Olds, 2006). Instead, filtering pipelines to allow population genetics analyses based on ddRAD data are designed to be conservative (Paris et al., 2017; Marandel et al., 2020). This filtering results in fewer loci retained, but it should reduce risks of including duplicates. In the current study, excess heterozygosity was observed consistently across populations, which could suggest historical introgression. Although we cannot completely rule out the influence of combining duplicates (Ilut, Nydam, & Hare, 2014), the highly consistent patterns of excess suggest that all populations would have been affected similarly, enabling interpretations of relative variation within and between populations. The admixture suggested by the STRUCTURE analyses also supports the role of introgression in the histories of the studied populations. However, mapping of the ddRAD reads to multiple reference genomes or to data generated based on different restriction enzymes would be more problematic.

Environmental associations

Despite the general lack of geographic clustering, there was evidence of local adaptation to the varying environments using redundancy analyses, particularly to annual precipitation (Figure 3). Although Watson-Jones et al. (2006) found some population structuring within the UK, the same result was not found in this study (i.e. no isolation-by-distance within the UK). Furthermore, no evidence of population structuring was found in the Spanish populations here, and Maggioni *et al.* (personal communication) found no evidence of population structuring in French Atlantic populations. These results could also be correlated with annual precipitation; perhaps the strong variation in annual precipitation in the UK (e.g.,

For Review Only

a strong west-east gradient) is causing more differentiation between these populations, whereas along the French range annual precipitation has a smaller gradient. One reason for the importance of annual precipitation other than water availability could be the influence of precipitation on soil pH. Soil pH is primarily determined by bedrock, but is also altered by precipitation through leaching of compounds such as calcium carbonate (Kinzel, 1983). Therefore, although slightly alkaline to neutral soils tend to form over limestone, secondary acidification can occur under higher precipitation regimes. The soil pH values recorded here ranged from neutral to strongly acidic (Figure 4). Furthermore, the bedrock of a large proportion of the populations used here (Table 2) differ from the limestone and chalk cliffs that wild *B. oleracea* are thought to be predominantly found on (Christensen et al., 2011). For those individuals where the soil pH was known, the same trend was found here as by Watson-Jones et al. (2006), with a decrease in plant genetic diversity as soil pH increased (Figure 4). For agriculture and horticulture, soil pH is an important consideration (Tilman, Balzer, Hill, & Befort, 2011). The change in plant genetic diversity suggests that soil pH is a strong selective pressure in the wild, causing an adaptive ecological bottleneck in locations where it is higher, resulting in lower genetic diversity. These indications of local adaptation despite a lack of population structure highlight environmental variables that could be investigated further in wild populations of *B. oleracea*, which regardless of their origin are surviving.

Alongside survival, a huge concern for food security related to climate change is the ability of crop plants to remain productive under rapidly changing environmental conditions (Lasky et al., 2015). Obtaining accurate phenotypic data for adaptive traits is a major barrier as we often do not know the combination of traits that underlie differences in fitness or how these vary with the environment (Kooyers, Greenlee, Colicchio, Oh, & Blackman, 2015). Although some traits will be locally adaptive due to large effect loci, the vast majority of adaptive traits are likely to have a polygenic basis (Rockman, 2012), particularly in the case of multi-trait phenotypes related to environmental gradients. Our results match these expectations, as no large effect loci were found; however, some were more significantly associated with the assessed environmental variation than others. The most likely assignment for six of the top 18 candidate genes was to the receptor-like kinase family (Table 4). This gene family underwent an expansion that is believed to be a plant-specific adaptation for pathogen defence (Afzal, Wood, & Lightfoot, 2008). Interestingly, Zhang et al. (2014) also found differences in genes related to plant defence

For Review Only

when investigating adaptations of rice (*Oryza* sp.) across four continents. These results highlight the fundamental importance of the immune system to fitness, and suggest that it could be related to environmental differences across different spatial scales. Given that immune system genes are among the best candidates for local adaptation, there is a potential connection between plant genetic diversity, soil pH and pathogens. It would be interesting to investigate whether less acidic soils host more pathogens, increasing the selective pressure on the plants and decreasing the plant genetic diversity in these soils. Overall, the impact of climate change on the spread of virulence of plant pathogens and herbivores, and the phenological mismatches that may occur between interacting species remain unknown (De Lucia, Nabity, Zavala, & Berenbaum, 2012; Fisher et al., 2012; Yang & Rudolf, 2010). What is clear is that plant defence will continue to be an important component of crop productivity, warranting further research.

Overall, the results presented here supported the hypothesis that wild populations of *B. oleracea* in the Atlantic region were established by plants from agricultural and/or horticultural sources. In addition, regardless of their origin, these wild populations are likely to contain useful genetic resources and should be considered as valuable populations of a crop wild relative to be investigated further.

Acknowledgements

EAM was funded by a University of Glasgow Lord Kelvin Adam Smith PhD studentship; UZI was funded by a NERC Independent Research Fellowship (NE/L011956); CAC is supported by the BBSRC (BB/P004202/1); KAM utilised equipment funded by the Wellcome Trust Institutional Strategic Support Fund (WT097835MF), Wellcome Trust Multi User Equipment Award (WT101650MA) and BBSRC LOLA award (BB/K003240/1). Part of the work was supported by a British Society for Plant Pathology summer studentship, and grants from the Botanical Research Fund, and the Blodwen Lloyd Bins trust funded through the Glasgow Natural History Society. None of the sponsors had any role in the study design, data collection, analysis, and interpretation or any aspects during the write up and publication of this work. We thank anonymous reviewers for helpful comments, Danijela Dimitrijević and Deborah Davy for assistance in the field, and Dr Lorenzo Maggioni for useful discussions.

For Review Only

Conflict of Interest

None declared.

References

Afzal, A. J., Wood, A. J., & Lightfoot, D. A. (2008). Plant receptor-like serine threonine kinases: roles in signaling and plant defense. *Molecular Plant-Microbe Interactions*, *21*(5), 507–517.

Allender, C., Allainguillaume, J., Lynn, J., & King, G. J. (2007). Simple sequence repeats reveal uneven distribution of genetic diversity in chloroplast genomes of *Brassica oleracea* L. and (n= 9) wild relatives. *Theoretical and Applied Genetics*, *114*(4), 609–618.

Andrews, K. R., Good, J. M., Miller, M. R., Luikart, G., & Hohenlohe, P. A. (2016). Harnessing the power of RADseq for ecological and evolutionary genomics. *Nature Reviews Genetics*, *17*(2), 81–92.

BGS. (2018). *British Geological Survey.* http://www.bgs.ac.uk/. (Accessed: 2018-08-26)

BSBI. (2018). *Botanical Society of Britain & Ireland.* https://bsbi.org. (Accessed: 2018-08-26)

Buckley, J., Holub, E. B., Koch, M. A., Vergeer, P., & Mable, B. K. (2018). Restriction associated DNA-genotyping at multiple spatial scales in *Arabidopsis lyrata* reveals signatures of pathogen-mediated selection. *BMC Genomics*, *19*(1), 496.

Charlesworth, D., Vekemans, X., Castric, V., & Glémin, S. (2005). Plant self-incompatibility systems: a molecular evolutionary perspective. *New Phytologist*, *168*(1), 61–69.

Christensen, S., von Bothmer, R., Poulsen, G., Maggioni, L., Phillip, M., Andersen, B. A., & Jørgensen, R. B. (2011). AFLP analysis of genetic diversity in leafy kale (*Brassica oleracea* L. convar. *acephala* (DC.) Alef.) landraces, cultivars and wild populations in Europe. *Genetic Resources and Crop Evolution*, *58*(5), 657–666.

Conner, J. K. (2002). Genetic mechanisms of floral trait correlations in a natural population. *Nature*, *420*(6914), 407–410.

Darwin, C. R. (1859). *The origin of species*. London: John Murray.

De Lucia, E., Nabity, P., Zavala, J., & Berenbaum, M. (2012). Climate change: resetting plant-insect interactions. *Plant Physiology*, pp–112.

Diamond, J. (2002). Evolution, consequences and future of plant and animal domestication. *Nature*, *418*(6898), 700.

Euro+Med PlantBase. (2020). http://ww2.bgbm.org/EuroPlusMed/. (Accessed: 2020-05-23)

Evanno, G., Regnaut, S., & Goudet, J. (2005). Detecting the number of clusters of individuals using the software STRUCTURE: a simulation study. *Molecular Ecology*, *14*(8), 2611–2620.

Falconer, D. S., & Mackay, T. F. C. (1996). *Introduction to Quantitative Genetics* (4th ed.). Harlow, UK: Longman.

Fick, S. E., & Hijmans, R. J. (2017). WorldClim 2: new 1-km spatial resolution climate surfaces for global land areas. *International Journal of Climatology*, *37*(12), 4302–4315.

Fisher, M. C., Henk, D. A., Briggs, C. J., Brownstein, J. S., Madoff, L. C., McCraw, S. L., & Gurr, S. J. (2012). Emerging fungal threats to animal, plant and ecosystem health. *Nature*, *484*(7393), 186.

Forester, B. R., Lasky, J. R., Wagner, H. H., & Urban, D. L. (2018). Comparing methods for detecting multilocus adaptation with multivariate genotype–environment associations. *Molecular Ecology*, *27*(9), 2215–2233.

Funk, J. L., Standish, R. J., Stock, W. D., & Valladares, F. (2016). Plant functional traits of dominant native and invasive species in mediterranean-climate ecosystems. *Ecology*, *97*(1), 75–83.

Gao, Y., Yin, S., Wu, L., Dai, D., Wang, H., Liu, C., & Tang, L. (2017). Genetic diversity and structure of wild and cultivated *Amorphophallus paeoniifolius* populations in southwestern China as revealed by RAD-seq. *Scientific Reports*, *7*(1), 14183.

Gómez-Campo, C., & Prakash, S. (1999). Origin and domestication. In *Developments in plant genetics and breeding* (Vol. 4, pp. 33–58). Elsevier.

Henriksen, R., Gering, E., & Wright, D. (2018). Feralisation—The understudied counterpoint to domestication. In *Origin and evolution of biodiversity* (pp. 183–195). Springer.

Hijmans, R. J. (2017). geosphere: Spherical Trigonometry [Computer software manual]. Retrieved from https://CRAN.R-project.org/package=geosphere (R package version 1.5-7)

For Review Only

Hoisington, D., Khairallah, M., Reeves, T., Ribaut, J.-M., Skovmand, B., Taba, S., & Warburton, M. (1999). Plant genetic resources: What can they contribute toward increased crop productivity? *Proceedings of the National Academy of Sciences*, *96*(11), 5937–5943.

Huson, D. H., & Bryant, D. (2005). Application of phylogenetic networks in evolutionary studies. *Molecular Biology and Evolution*, *23*(2), 254–267.

IGME. (2018). *Instituto Geológico y Minero de España.* `http://www.igme.es`. (Accessed: 2018-08-26)

Ilut, D. C., Nydam, M. L., & Hare, M. P. (2014). Defining loci in restriction-based reduced representation genomic data from nonmodel species: sources of bias and diagnostics for optimal clustering. *BioMed Research International*, *2014*.

Kinzel, H. (1983). Influence of limestone, silicates and soil pH on vegetation. In *Physiological plant ecology iii* (pp. 201–244). Springer.

Kitashiba, H., & Nasrallah, J. B. (2014). Self-incompatibility in Brassicaceae crops: lessons for interspecific incompatibility. *Breeding Science*, *64*(1), 23–37.

Kofsky, J., Zhang, H., & Song, B.-H. (2018). The untapped genetic reservoir: The past, current, and future applications of the wild soybean (*Glycine soja*). *Frontiers in Plant Science*, *9*.

Kooyers, N. J., Greenlee, A. B., Colicchio, J. M., Oh, M., & Blackman, B. K. (2015). Replicate altitudinal clines reveal that evolutionary flexibility underlies adaptation to drought stress in annual *Mimulus guttatus*. *New Phytologist*, *206*(1), 152–165.

Langmead, B., & Salzberg, S. L. (2012). Fast gapped-read alignment with Bowtie 2. *Nature Methods*, *9*(4), 357.

Lanner-Herrera, C., Gustafeson, M., Filt, A., & Bryngelsson, T. (1996). Diversity in natural populations of wild *Brassica oleracea* as estimated by isozyme and RAPD analysis. *Genetic Resources and Crop Evolution*, *43*(1), 13–23.

Lasky, J. R., Upadhyaya, H. D., Ramu, P., Deshpande, S., Hash, C. T., Bonnette, J., ... others (2015). Genome-environment associations in sorghum landraces predict adaptive traits. *Science Advances*, *1*(6), e1400218.

Lázaro, A., & Aguinagalde, I. (1998). Genetic diversity in *Brassica oleracea* L.(Cruciferae) and wild relatives (2 n= 18) using isozymes. *Annals of Botany*, *82*(6), 821–828.

Li, H., Handsaker, B., Wysoker, A., Fennell, T., Ruan, J., Homer, N., ... Durbin, R. (2009). The sequence alignment/map format and SAMtools. *Bioinformatics*, *25*(16), 2078–2079.

Liu, S., Liu, Y., Yang, X., Tong, C., Edwards, D., Parkin, I. A., ... others (2014). The *Brassica oleracea* genome reveals the asymmetrical evolution of polyploid genomes. *Nature Communications*, *5*.

Maggioni, L. (2015). *Domestication of* Brassica oleracea *l.* (Unpublished doctoral dissertation). Swedish University of Agricultural Studies, Alnarp, Sweden.

Maggioni, L., von Bothmer, R., Poulsen, G., & Lipman, E. (2018). Domestication, diversity and use of *Brassica oleracea* L., based on ancient Greek and Latin texts. *Genetic Resources and Crop Evolution*, *65*(1), 137–159.

Marandel, F., Charrier, G., Lamy, J.-B., Le Cam, S., Lorance, P., & Trenkel, V. M. (2020). Estimating effective population size using RADseq: Effects of SNP selection and sample size. *Ecology and Evolution*, *10*(4), 1929–1937.

Martin, M. (2011, May). Cutadapt removes adapter sequences from high-throughput sequencing reads. *EMBnet.journal*, *17*(1), 10–12. Retrieved from `http://journal.embnet.org/index.php/embnetjournal/article/view/200` doi: http://dx.doi.org/10.14806/ej.17.1.200

Milla, R., Osborne, C. P., Turcotte, M. M., & Violle, C. (2015). Plant domestication through an ecological lens. *Trends in Ecology & Evolution*.

Mitchell, N. (1976). The Status of *Brassica oleracea* L. subsp. *oleracea* (wild cabbage) in the British Ilses. *Watsonia*, *11*, 97–103.

Nazareno, A. G., Bemmels, J. B., Dick, C. W., & Lohmann, L. G. (2017). Minimum sample sizes for population genomics: An empirical study from an Amazonian plant species. *Molecular Ecology Resources*, *17*(6), 1136–1147.

Nei, M. (1973). Analysis of gene diversity in subdivided populations. *Proceedings of the National Academy of Sciences*, *70*(12), 3321–3323.

Oksanen, J., Blanchet, F. G., Friendly, M., Kindt, R., Legendre, P., McGlinn, D., ... Wagner, H. (2017). vegan: Community Ecology Package [Computer software manual]. Retrieved from `https://CRAN.R-project.org/package=vegan` (R package version 2.4-3)

For Review Only

Paris, J. R., Stevens, J. R., & Catchen, J. M. (2017). Lost in parameter space: a road map for stacks. *Methods in Ecology and Evolution*, *8*(10), 1360–1373.

Prentis, P. J., Wilson, J. R., Dormontt, E. E., Richardson, D. M., & Lowe, A. J. (2008). Adaptive evolution in invasive species. *Trends in Plant Science*, *13*(6), 288–294.

Pritchard, J. K., Stephens, M., & Donnelly, P. (2000). Inference of population structure using multilocus genotype data. *Genetics*, *155*(2), 945–959.

Purugganan, M. D., & Fuller, D. Q. (2009). The nature of selection during plant domestication. *Nature*, *457*(7231), 843–848.

Quinlan, A. R., & Hall, I. M. (2010). BEDTools: a flexible suite of utilities for comparing genomic features. *Bioinformatics*, *26*(6), 841–842.

R Core Team. (2017). R: A language and environment for statistical computing [Computer software manual]. Vienna, Austria. Retrieved from `https://www.R-project.org/`

Rauf, S., Teixeira da Silva, J., Khan, A. A., & Naveed, A. (2010). Consequences of plant breeding on genetic diversity. *International Journal of Plant Breeding*, *4*(1), 1–21.

Raybould, A., Mogg, R., Clarke, R., Gliddon, C., & Gray, A. (1999). Variation and population structure at microsatellite and isozyme loci in wild cabbage (*Brassica oleracea* L.) in Dorset (UK). *Genetic Resources and Crop Evolution*, *46*(4), 351–360.

Revelle, W. (2018). psych: Procedures for Psychological, Psychometric, and Personality Research [Computer software manual]. Evanston, Illinois. Retrieved from `https://CRAN.R-project.org/package=psych` (R package version 1.8.10)

Rochette, N. C., & Catchen, J. M. (2017). Deriving genotypes from RAD-seq short-read data using Stacks. *Nature Protocols*, *12*(12), 2640.

Rockman, M. V. (2012). The QTN program and the alleles that matter for evolution: all that's gold does not glitter. *Evolution*, *66*(1), 1–17.

Rousset, F., & Raymond, M. (1995). Testing heterozygote excess and deficiency. *Genetics*, *140*(4), 1413–1419.

Sánchez-Yélamo, M. (2014). Characterisation of wild cabbage (*Brassica oleracea* L.) based on isoenzyme data. considerations on the current status of this taxon in Spain. *Genetic Resources and Crop Evolution*, *61*(7), 1295–1306.

Scheepens, J., Frei, E. S., Armbruster, G. F., & Stöcklin, J. (2012). Pollen dispersal and gene flow within and into a population of the alpine monocarpic plant *Campanula thyrsoides*. *Annals of Botany*, *110*(7), 1479–1488.

Schranz, M. E., Lysak, M. A., & Mitchell-Olds, T. (2006). The ABC's of comparative genomics in the Brassicaceae: building blocks of crucifer genomes. *Trends in Plant Science*, *11*(11), 535–542.

Sharbel, T. F., Haubold, B., & Mitchell-Olds, T. (2000). Genetic isolation by distance in *Arabidopsis thaliana*: biogeography and postglacial colonization of Europe. *Molecular Ecology*, *9*(12), 2109–2118.

Slatkin, M. (1987). Gene flow and the geographic structure of natural populations. *Science*, *236*(4803), 787–792.

Song, K., Osborn, T. C., & Williams, P. H. (1990). *Brassica* taxonomy based on nuclear restriction fragment length polymorphisms (RFLPs). *Theoretical and Applied Genetics*, *79*(4), 497–506.

Stamatakis, A. (2014). RAxML version 8: a tool for phylogenetic analysis and post-analysis of large phylogenies. *Bioinformatics*, *30*(9), 1312–1313.

Stansell, Z., Hyma, K., Fresnedo-Ramírez, J., Sun, Q., Mitchell, S., Björkman, T., & Hua, J. (2018). Genotyping-by-sequencing of *Brassica oleracea* vegetables reveals unique phylogenetic patterns, population structure and domestication footprints. *Horticulture Research*, *5*.

Tilman, D., Balzer, C., Hill, J., & Befort, B. L. (2011). Global food demand and the sustainable intensification of agriculture. *Proceedings of the National Academy of Sciences*, *108*(50), 20260–20264.

USDA, D. o. A. (1998). *Soil Quality Indicators: pH.* `https://www.nrcs.usda.gov/Internet/FSE_DOCUMENTS/nrcs142p2_052208.pdf`. (Accessed: 2016-04-20)

von Wettberg, E. J., Chang, P. L., Başdemir, F., Carrasquila-Garcia, N., Korbu, L. B., Moenga, S. M., ... others (2018). Ecology and genomics of an important crop wild relative as a prelude to agricultural innovation. *Nature Communications*, *9*(1), 649.

Walley, P. G., Teakle, G. R., Moore, J. D., Allender, C. J., Pink, D. A., Buchanan-Wollaston, V., & Barker, G. C. (2012). Developing genetic resources for pre-breeding in *Brassica oleracea* L.: an overview of the UK perspective. *Journal of Plant Biotechnology*, *39*(1), 62–68.

For Review Only

Watson-Jones, S., Maxted, N., & Ford-Lloyd, B. (2006). Population baseline data for monitoring genetic diversity loss for 2010: a case study for *Brassica* species in the UK. *Biological Conservation*, *132*(4), 490–499.
Wichmann, M. C., Alexander, M. J., Soons, M. B., Galsworthy, S., Dunne, L., Gould, R., ... Bullock, J. M. (2009). Human-mediated dispersal of seeds over long distances. *Proceedings of the Royal Society of London B: Biological Sciences*, *276*(1656), 523–532.
Wu, Z., Wang, B., Chen, X., Wu, J., King, G. J., Xiao, Y., & Liu, K. (2016). Evaluation of linkage disequilibrium pattern and association study on seed oil content in *Brassica napus* using ddRAD sequencing. *PloS One*, *11*(1), e0146383.
Xiao, Z., Han, F., Hu, Y., Xue, Y., Fang, Z., Yang, L., ... others (2019). Overcoming cabbage crossing incompatibility by the development and application of self-compatibility-QTL-specific markers and genome-wide background analysis. *Frontiers in Plant Science*, *10*, 189.
Yang, L. H., & Rudolf, V. (2010). Phenology, ontogeny and the effects of climate change on the timing of species interactions. *Ecology Letters*, *13*(1), 1–10.
Yousef, E. A., Mueller, T., Börner, A., & Schmid, K. J. (2018). Comparative analysis of genetic diversity and differentiation of cauliflower (*Brassica oleracea* var. botrytis) accessions from two *ex situ* genebanks. *PLoS One*, *13*(2), e0192062.
Yu, J., Zhao, M., Wang, X., Tong, C., Huang, S., Tehrim, S., ... Liu, S. (2013). Bolbase: a comprehensive genomics database for *Brassica oleracea*. *BMC Genomics*, *14*(1), 664.
Zhang, Q.-J., Zhu, T., Xia, E.-H., Shi, C., Liu, Y.-L., Zhang, Y., ... others (2014). Rapid diversification of five *Oryza* AA genomes associated with rice adaptation. *Proceedings of the National Academy of Sciences*, *111*(46), E4954–E4962.

Data Accessibility

The sequencing data and associated meta data are available on the European Nucleotide Archive under the study accession number: PRJEB38464 (`http://www.ebi.ac.uk/ena/data/view/PRJEB38464`)

Author Contributions

EAM and BKM conceived and proposed the study to the co-authors; EAM, BKM, CAC, and UZI then developed the study design. BKM, CAC, and UZI obtained the main funding award. EAM and EAK collected the data. EAM carried out statistical analysis, and wrote the paper. KAM carried out the double-digest RAD-sequencing. All authors reviewed the final version of the manuscript and agreed to its content before submission.

For Review Only

Table 1: Estimates of genetic diversity within wild *B. oleracea* populations from previous studies using different molecular markers. H_E is expected heterozygosity estimated using Nei's gene diversity (Nei, 1973).

Study	Molecular marker	H_E	Populations
Lanner-Herrera et al. (1996)	Isozymes	0.10 – 0.56	France, Spain, UK
Lázaro and Aguinagalde (1998)	Isozymes	0.26 – 0.30	France, Spain, UK
Raybould et al. (1999)	Isozymes	0.40 (0.18 – 0.41)†	UK
Raybould et al. (1999)	Microsatellites	0.36 (0.21 – 0.33)†	UK
Watson-Jones et al. (2006)	AFLPs	0.19 – 0.33	UK
Christensen et al. (2011)	AFLPs	0.23, 0.20	Spain, UK
Maggioni *et al.* (pers. comm. 2019)	AFLPs	0.25	France

† – pooled population H_E with the range of estimates from individual populations shown in brackets.

For Review Only

Table 2: A summary of the natural populations of *B. oleracea* used in this study, including: the bedrock, the first time the population was recorded, the number of individuals sequenced, and the number of individuals included in down-stream analyses.

Region	Population	Bedrock†	First population record‡	Number sequenced	Number included§
ES	Auchmithie	Red basic sandstone	1913	4	4
ES	Crail	Sandstone & limestone	1840	4	4
ES	Fortrose	Sandstone	1968	4	3
WS	Kildonan Castle	Sandstone & limestone	1987	4	4
NEE	Tynemouth	Sandstone & limestone	1805	4	4
NEE	Staithes	Shale & sandstone	1831	4	–
NEE	Whitby	Shale	1906	4	4
NW	Little Orme	Limestone	1895	4	–
SW	Tenby	Siltstone & sandstone	1773	4	4
SW	Llantwit Major	Limestone	1850	4	4
SWE	Prussia Cove	Slate, shale & siltstone	1871	4	4
SWE	Fowey	Shale & siltstone	1805	4	4
SWE	West Looe	Siltstone & sandstone	1971	4	2
SWE	St. Aldhelm's Head	Limestone	1933	4	4
A	Cudillero	Slate & sandstone		4	4
A	Playa de Xágo	Sandstone & Dolomite		4	4
A	Cabo de Peñas	Slate & quartzite		4	4
A	Playas de Viodo	Slate & shale		4	4
A	Tazones	Dolomite & limestone		4	–
C	Playa Pedrero	Quartzites		4	4
C	La Franca	Quartzites		4	–
BC	San Juan de Gaxtelugatxe	Limestone		4	4
BC	Getaría	Limestone		4	4
BC	San Sebastian	Calcareous sandstone		4	3
			**Total:**	96	76

† data obtained from the British Geological Survey (https://www.bgs.ac.uk) and the Instituto Geológico y Minero de España (http://www.igme.es). Region codes: ES – East Scotland, WS – West Scotland, NEE – North-eastern England, NW – North Wales, SW – South Wales, SWE – South-western England, A – Asturias Spain, C – Cantabrica Spain, BC – Basque Country Spain. ‡ data obtained from the Botanical Society of Britain & Ireland (https://bsbi.org). § indicates where data was lost in quality filtering of sequences and not included in down-stream analyses.

For Review Only

24

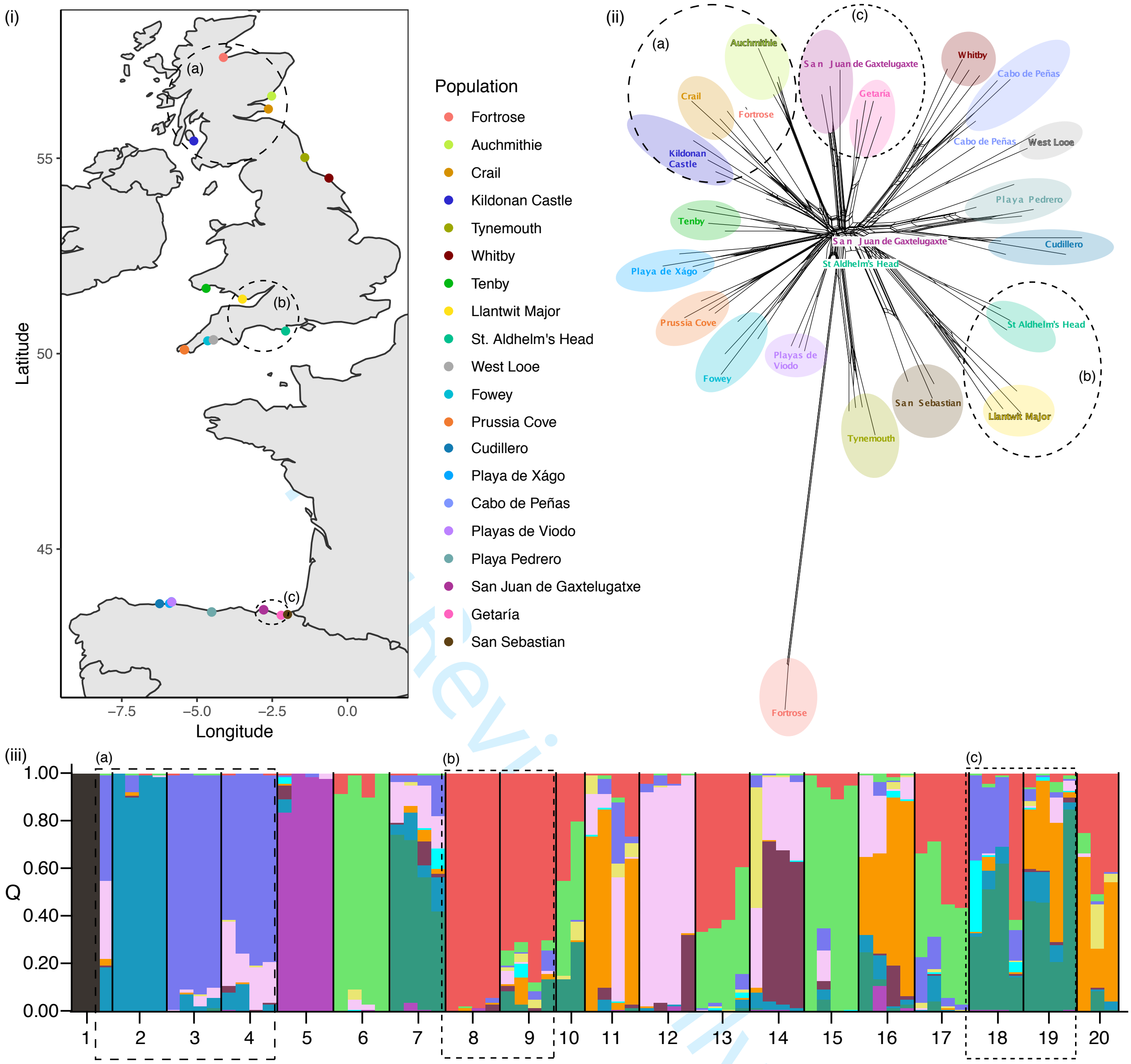

Figure 1: Population structuring of wild populations of *B. oleracea*. (i) Location of the populations considered here. (ii) Clustering of samples from RAxML (v8.2; GTRCAT model and 1000 maximum likelihood bootstrap replicates), visualised in SplitsTree4. (iii) STRUCTURE plot illustrating shared genetic ancestry for K = 12, ordered by population: 1 – Fortrose; 2 – Auchmithie; 3 – Crail; 4 – Kildonan Castle; 5 – Tynemouth; 6 – Whitby; 7 – Tenby; 8 – Llantwit Major; 9 – St. Aldhelm's Head; 10 – West Looe; 11 – Fowey; 12 – Prussia Cova; 13 – Cudillero; 14 – Playa de Xágo; 15 – Cabo de Peñas; 16 – Playas de Viodo; 17 – Playa Pedrero; 18 – San Juan de Gaxtelugatxe; 19 – Getaría; 20 – San Sebastian. Across the figures the same colours and numbering is used for each population. The dashed lines and letters indicate some clustering: (a) populations in Scotland; (b) populations closest to the Welsh-English border; and (c) populations in the Basque Country, Spain (excluding San Sebastian).

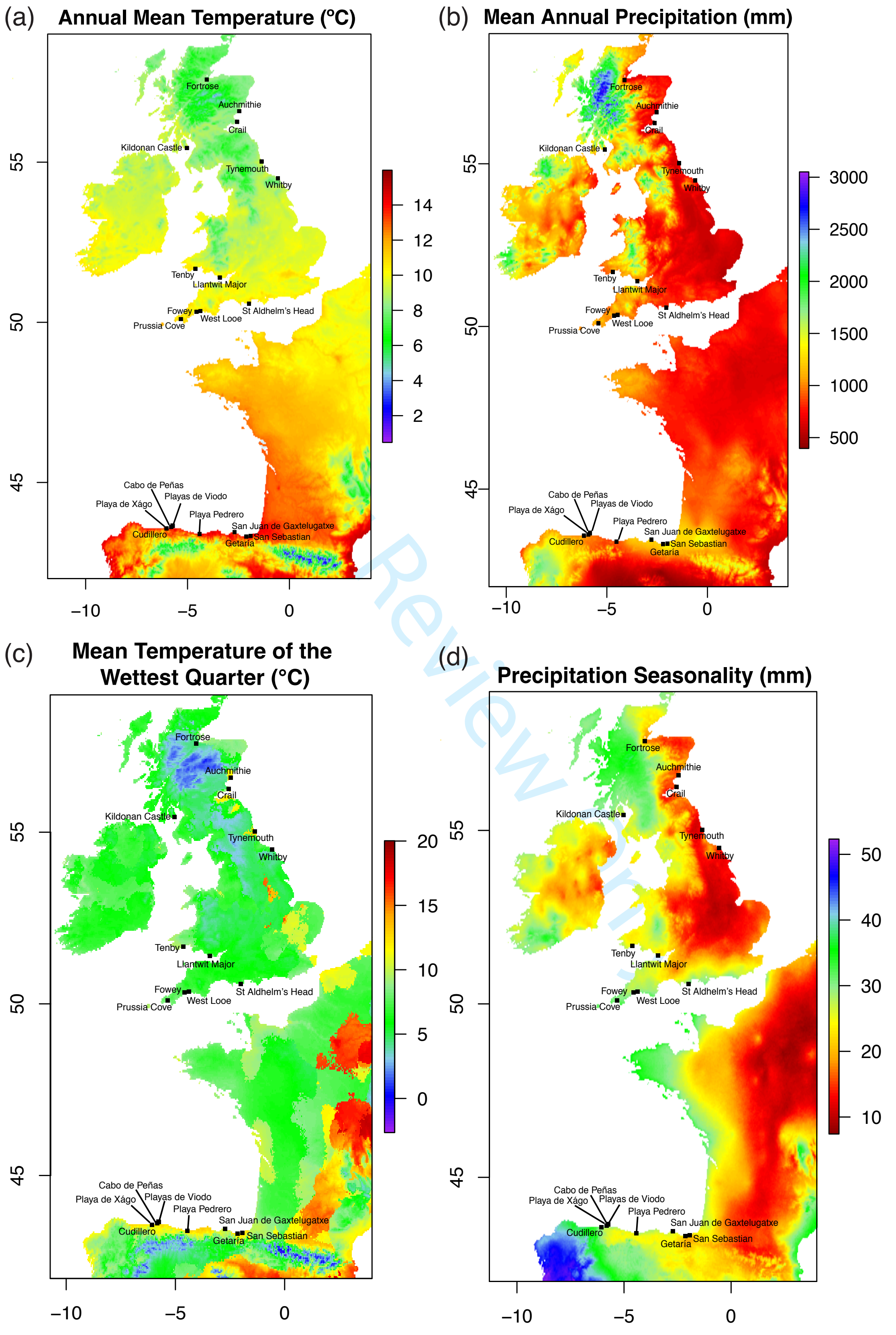

Figure 2: The distribution of sampled populations in relation to various climate variables: (a) annual mean temperature (℃); (b) mean annual precipitation (mm); (c) mean temperature of wettest quarter (℃); (d) precipitation seasonality (mm). These are averages between 1970 – 2000 obtained from the WorldClim database (Fick & Hijmans, 2017).

Table 3: Summary statistics of within *B. oleracea* population genetic diversity based on both variant nucleotide sites alone (var) and all sites (all) from dataset 1, showing: the number of individuals (N), the number of private alleles (PRI), expected heterozygosity (H_E), observed heterozygosity (H_O) and percentage of polymorphic loci (%).

Region†	Population	N	PRI	H_E		H_O		%
				Var	*All*	*Var*	*All*	*All*
ES	Authmithie	4	1683	0.1043	0.0012	0.1202	0.0014	33.0
ES	Crail	4	1727	0.1327	0.0019	0.1267	0.0018	52.8
ES	Fortrose	3	12951	0.2006	0.0032	0.1962	0.0031	76.4
WS	Kildonan Castle	4	1014	0.0903	0.0014	0.0944	0.0014	40.8
NEE	Tynemouth	4	1476	0.1023	0.0013	0.0881	0.0011	36.4
NEE	Whitby	4	1573	0.1200	0.0020	0.1184	0.0020	56.7
SW	Tenby	4	1568	0.1227	0.0014	0.1153	0.0013	40.5
SW	Llantwit Major	4	2073	0.1390	0.0023	0.1231	0.0022	66.2
SWE	Prussia Cove	4	1454	0.1019	0.0016	0.1064	0.0017	45.5
SWE	Fowey	4	1137	0.1126	0.0018	0.1083	0.0017	53.4
SWE	West Looe	2	1412	0.1150	0.0011	0.1328	0.0013	27.1
SWE	St. Aldhelm's Head	4	2470	0.1486	0.0014	0.1676	0.0016	39.4
A	Cudillero	4	716	0.0918	0.0015	0.0938	0.0016	44.3
A	Playa de Xágo	4	1583	0.1140	0.0012	0.1191	0.0012	33.4
A	Cabo de Peñas	4	698	0.0933	0.0015	0.0910	0.0014	42.5
A	Playas de Viodo	4	503	0.0545	0.0004	0.0580	0.0004	11.2
C	Playa Pedrero	4	1741	0.1313	0.0014	0.1408	0.0015	38.5
BC	San Juan de Gaxtelugatxe	4	2608	0.1423	0.0012	0.1471	0.0012	34.0
BC	Getaría	4	1550	0.1280	0.0021	0.1391	0.0023	59.8
BC	San Sebastian	3	2516	0.1530	0.0023	0.1538	0.0023	61.4

†Region codes: ES – East Scotland, WS – West Scotland, NEE – North-eastern England, SW – South Wales, SWE – South-western England, A – Asturias Spain, C – Cantabrica Spain, BC – Basque Country Spain.

For Review Only

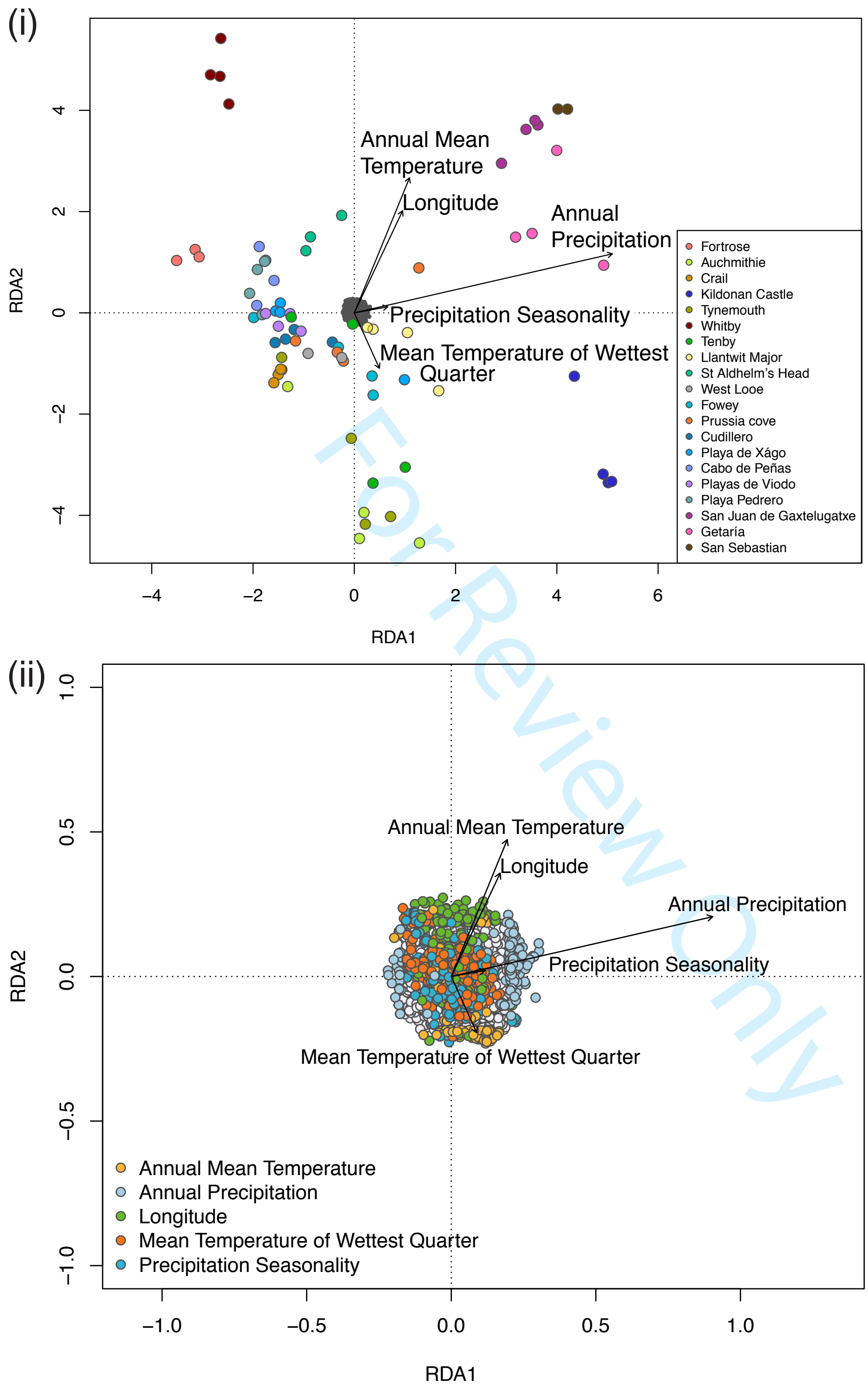

Figure 3: (i) Redundancy analysis (RDA) ordination plot of the association between *B. oleracea* individuals (coloured points) and SNPs (dark grey points), with environmental variables. The different colours indicate which population each individual was from. (ii) RDA ordination plot of the SNPs alone, coloured for the environmental variable with which they were most strongly associated. For both (i) & (ii) the arrows indicate the environmental predictors and the strength of the association.

For Review Only

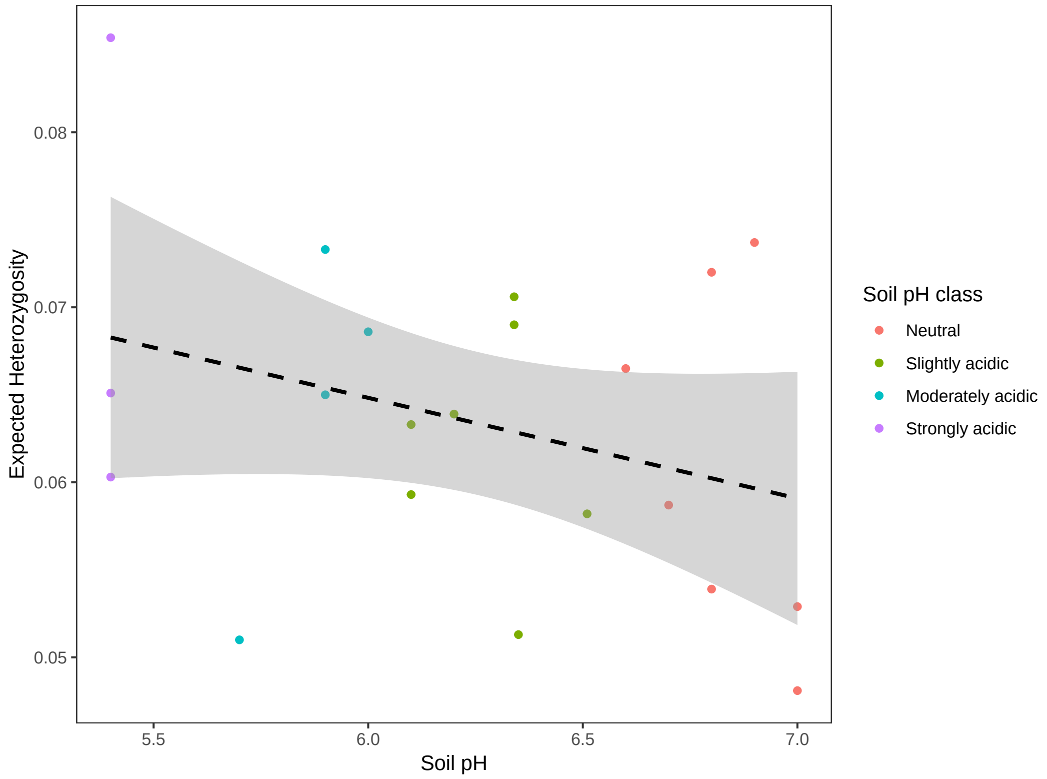

Figure 4: The relationship between expected heterozygosity and soil pH for 21 individuals from four soil pH classes categorised into: Neutral (6.6 - 7.3), Slightly acidic (6.1 - 6.5), Moderately acidic (5.6- 6.0) and Strongly acidic (5.0 - 5.5) based on USDA (1998). A linear model was used to fit a regression line (dashed black line), the standard error is shown in grey, p-value $>$ 0.05.

Table 4: The top 18 candidate SNPs that mapped to unique genes in the *B. oleracea* reference genome and their annotations from 'Bolbase' (Yu et al., 2013).

Chromosome	Location	Identity	X	Bolbase gene name	Potential protein	Function
C09	32879582	1	-	Bol019890	Ribonucleotide reductase-related	Fatty acid metabolic process, creation of DNA from RNA
C04	39737611	0.999979	-	Bol021601	Unknown	
C09	8499546	1	+	Bol032146	Basic helix-loop-helix dimerisation region	Nucleus transcription regulation
C07	43014116	1	-	Bol042101	Toll-Interleukin receptor	Signal transduction, immune response, disease resistance
C02	233586	1	+	Bol012817	Laccase/multicopper oxidase	Copper ion binding, metabolic process, maybe formation and degradation of lignin
C04	22051514	0.999656	+	Bol044300	Protein kinase - serine/threonine	Protein kinase activity, signalling, plant defence
C03	29308196	0.472347	-	Bol012462	PIK-related kinase	Binding and DNA repair
C03	48963472	0.99438	+	Bol029900	Protein kinase	Protein kinase activity, signalling, plant defence
C04	28456859	0.999661	-	Bol009961	Cystathionine beta-synthase	Vitamin B6 pathway?
C03	9456274	1	-	Bol005573	Unknown	
C05	2317477	0.580051	-	Bol041075	Pentotricopeptide repeat	Often essential in mitochondria
C04	35972614	0.304057	+	Bol037830	Bacterial transferase haxapeptide repeat	Binding and transferase activity
C04	35104965	0.996501	+	Bol037950	Cyclin-like F-box	Growth and development
C03	2461137	0.999261	-	Bol034275	Serine/threonine-protein kinase	Signalling, plant defence
C02	233586	0.168963	-	Bol012816	Serine/threonine-protein kinase	Signalling, plant defence
C01	11164295	0.999978	+	Bol039465	Initiation factor eIF-4 gamma, MA3	
C01	11431159	1	+	Bol039505	Heat shock protein Hsp20	
C01	12106862	0.918256	-	Bol039585	F-box associated	

For Peer Review Only

Title: Feral populations of *Brassica oleracea* along Atlantic coasts in western Europe

Running title: Feral *Brassica oleracea* in western Europe

Elizabeth A. Mittell[1,2,*], Christina A. Cobbold[3,4], Umer Zeeshan Ijaz[5], Elizabeth A. Kilbride[1], Karen A. Moore[6] & Barbara K. Mable[1,4]†

[1]Institute of Biodiversity, Animal Health and Comparative Medicine, University of Glasgow, UK; [2]School of Biology, University of St Andrews, UK; [3]School of Mathematics and Statistics, University of Glasgow, UK; [4]The Boyd Orr Centre for Population and Ecosystem Health, University of Glasgow, Glasgow, UK; [5]School of Engineering, University of Glasgow, UK; [6]Exeter Sequencing Service, University of Exeter, UK

Corresponding authors: Elizabeth A. Mittell, *em294@st-andrews.ac.uk, e.mittell@gmail.com & Barbara K. Mable, † Barbara.Mable@glasgow.ac.uk

For Review Only

Abstract

There has been growing emphasis on the role that crop wild relatives might play in supporting highly selected agriculturally valuable species in the face of climate change. In species that were domesticated many thousands of years ago, distinguishing wild populations from escaped feral forms can be challenging, but reintroducing variation from either source could supplement current cultivated forms. For economically important cabbages (Brassicaceae: *Brassica oleracea*), "wild" populations occur throughout Europe but little is known about their genetic variation or potential as resources for breeding more resilient crop varieties. The main aim of this study was to characterise the population structure of geographically isolated wild cabbage populations along the coasts of the UK and Spain, including the Atlantic range edges. Double-digest restriction-site associated DNA sequencing was used to sample individual cabbage genomes, assess the similarity of plants from 20 populations, and explore environment-genotype associations across varying climatic conditions. Interestingly, there were no indications of isolation-by-distance; several geographically close populations were genetically more

distinct from each other than to distant populations. Furthermore, several distant populations shared genetic ancestry, which could indicate that they were established by escapees of similar source cultivars. However, there were signals of local adaptation to different environments, including a possible relationship between genetic diversity and soil pH. Overall, these results highlight wild cabbages in the Atlantic region as an important genetic resource worthy of further research into their relationship with existing crop varieties.

Keywords: *Brassica oleracea*, feral populations, crop wild relatives, isolation-by-distance, environment-genotype associations, domestication

For Review Only

Introduction

Domestication was an important transition within human societies, which allowed the rise of civilisations (Diamond, 2002). Whilst vital for human success, there have been evolutionary consequences for the domesticated organisms. In crop plants, the selection of ‘domestication traits’ has led to many desired changes in physiological, morphological and life-history traits compared to their wild relatives (Milla, Osborne, Turcotte, & Violle, 2015; Purugganan & Fuller, 2009). However, traits that are correlated with those selected for (directly or indirectly) can also influence phenotypes via pleiotropic effects (Conner, 2002) and linkage disequilibrium (Falconer & Mackay, 1996). These genetic constraints and narrow population bottlenecks can have unintended genetic consequences for crop plants, particularly elite lines that are the result of intense artificial selection; e.g., reduced genetic diversity, increased genetic drift and increased deleterious allele frequencies (Rauf, Teixeira da Silva, Khan, & Naveed, 2010; von Wettberg et al., 2018). It is also likely that crop lines are constrained to some extent by the environment within which they were originally domesticated. Therefore, to continue to utilise crop plants successfully, it is important to understand both the genetic consequences of domestication, and where it occurred.

A classic example of domestication can be found in the commercially valuable species, *Brassica oleracea* (recognised by Darwin, 1859; Walley et al., 2012). This single species contains a huge amount of morphological diversity in cultivated varieties that has been around since at least the 1st Century (e.g., kale, kohlrabi, broccoli, Brussels sprouts and cauliflower; Maggioni, von Bothmer, Poulsen, & Lipman, 2018); the same morphological extremes are not found in wild populations. The origin of domesticated

B. oleracea crops and the 'wild' or 'feral' status of populations, found throughout the UK and along the Atlantic coasts of north-western Europe (Raybould, Mogg, Clarke, Gliddon, & Gray, 1999), has been debated in the literature (Allender, Allainguillaume, Lynn, & King, 2007; Gómez-Campo & Prakash, 1999; Maggioni, 2015; Mitchell, 1976). Initially it was thought that different cultivars were independently domesticated from wild populations on European Atlantic coasts (e.g., Spanish cabbage varieties were domesticated from local wild Spanish populations; Gómez-Campo & Prakash, 1999), and that early domesticates were introduced to and diversified within the Mediterranean region around 3,000 to 4,000 years ago (Allender et al., 2007). Information was limited when this hypothesis was favoured (Allender et al., 2007; Gómez-Campo & Prakash, 1999), although there was already conflicting evidence (Mitchell, 1976). For example, Mitchell (1976) found that the locations of ancient human settlements and modern *B. oleracea* populations coincided along UK coasts, providing a potential source of escapees from domestic settings (agriculture or gardens) that could have established feral populations. This alternative hypothesis that *B. oleracea* originated elsewhere and escaped into the wild in the Atlantic region has been supported by recent linguistic and historical research (Maggioni, 2015; Maggioni et al., 2018). Maggioni (2015) suggested that the most plausible hypothesis is that *B. oleracea* was domesticated in the Mediterranean region, before being moved across Europe by people, where escaped plants established now naturalised populations. However, the genetic status of *B. oleracea* in the Atlantic region is still an open question (*B. oleracea* is classified as a native species in the UK and an alien species in Spain; *Euro+Med PlantBase*, 2020).

The ease with which cultivated and wild *B. oleracea* plants can introgress is an issue for interpreting variation within the *B. oleracea* species complex, as past hybridisation can obscure phylogeographic signals (Allender et al., 2007). However, for crop breeding purposes a close genetic relationship between wild populations and domesticated cultivars may be seen as an advantage; higher genetic similarity could make it easier to introgress adaptive traits from the wild into cultivated varieties (Hoisington et al., 1999). An alternative view is that if these populations are feral they would have experienced the same domestication bottleneck as many cultivars (von Wettberg et al., 2018), and therefore they may not be the important reservoirs of genetic diversity that crop wild relatives are typically assumed to be. Compared to domestication, feralization is under-investigated; however modern genomic data are al-

For Review Only

lowing its occurrence to be identified and consequences better understood (see examples in Henriksen, Gering, & Wright, 2018). Despite the agricultural importance of *B. oleracea*, there has not yet been a comprehensive genetic analysis of wild populations in the Atlantic region that would allow assessment of their utility as sources of variation for cultivation.

Escaped plants can be thought of as 'invasive' species, which are defined as those that became established after introduction outside of the biogeographic region within which they evolved (Prentis, Wilson, Dormontt, Richardson, & Lowe, 2008). However, it is not always clear where these 'native' regions are located, as is the case of *B. oleracea*, or why certain species are successful where others are not. Furthermore, wild populations of *B. oleracea* do not have the characteristics that are thought to be important for successful establishment in novel locations (i.e. 'invasive traits'; Funk, Standish, Stock, & Valladares, 2016). For example, wild *B. oleracea* are: perennials rather than annuals, woody rather than herbaceous, relatively slow- rather than fast-growing, and predominantly outcrossing rather than selfing. Self-fertilisation in plants is inhibited by polymorphic self-incompatibility (SI) recognition systems where haplotype blocks encode distinct proteins for pollen-pistil recognition (Charlesworth, Vekemans, Castric, & Glémin, 2005). A strong SI system exists in *B. oleracea* (a single-locus system with over 60 alleles; Raybould et al., 1999), making them predominantly self-incompatible (Kitashiba & Nasrallah, 2014; Walley et al., 2012; Yousef, Mueller, Börner, & Schmid, 2018). Development of self-compatible lines can aid in propagation of cultivated forms (e.g., Xiao et al., 2019), but reduce adaptive potential to changing environmental conditions. Therefore, even if the "wild" populations include escaped forms, retention of a wide range of self-incompatibility alleles could be used to enhance the potential of breeding strategies designed to maintain heterosis.

Currently too little is known about levels of genetic variation and population structure in wild *B. oleracea* populations to fully assess the potential for use of plants from different regions to supplement crop diversity. Population structure and within population genetic diversity are impacted by gene flow, which occurs via pollen and seeds in plants (Scheepens, Frei, Armbruster, & Stöcklin, 2012; Slatkin, 1987). The main pollinators of *B. oleracea* are bees that fly short distances between plants (average 2 m; Raybould et al., 1999). Seed dispersal was previously thought to be limited to approximately 4 m (Watson-Jones, Maxted, & Ford-Lloyd, 2006). However, Wichmann et al. (2009) found that wind can

For Review Only

spread seeds up to 250 m, and that rare-long distance dispersal events of up to 10 km could occur if seeds became attached to people's shoes. Therefore, although gene flow may be limited between geographically close populations leading to high genetic structuring in some instances, in other cases, such as where plants grow close (0 - 4 m) to well used coastal paths, gene flow might be greater than expected. Genetic diversity estimates have been made in some *B. oleracea* populations within the Atlantic region (e.g., Table 1), but the northern edge (Scotland) has not been investigated. A correlation between genetic distance and geographic distance in wild *B. oleracea* populations was found in some studies (Raybould et al., 1999; Sánchez-Yélamo, 2014) but not others (Christensen et al., 2011; Watson-Jones et al., 2006). Interestingly, Watson-Jones et al. (2006) also considered some environmental variables and found that higher soil pH was associated with lower genetic diversity in English and Welsh populations. The inconsistency in previous studies could be due to the varying spatial scales and molecular markers used. However, overall, these results highlight the uncertainty in the status and genetic contents of wild *B. oleracea* populations in the Atlantic region, as well as the potential effect of environment on the plant genetics. Filling these knowledge gaps could provide important insights into these crop wild relatives for agricultural use.

Brassica oleracea is a good model for investigating the genetic resources available (e.g., the extent of genetic diversity and local adaptation) in a potentially feral crop wild relative because it is diploid and a reference genome is available (Liu et al., 2014). Therefore, compared to other crop species (e.g., polyploids) genetic analyses are simpler. For many questions whole-genome sequencing is unnecessary (Rockman, 2012) and reduced-representation methods, such as double-digest restriction associated DNA sequencing (ddRADseq), are sufficient to: assess genetic diversity within and between populations (Andrews, Good, Miller, Luikart, & Hohenlohe, 2016); determine population genetic structuring (Gao et al., 2017); and investigate potential associations between genotypes and environmental variables (Forester, Lasky, Wagner, & Urban, 2018). Therefore, ddRADseq is an appropriate method for considering the genetic resources in, and local adaptation of, *B. oleracea* populations across their Atlantic range.

Overall, current knowledge on genetic variation of *B. oleracea* in wild populations is patchy in geographic coverage and based on outdated molecular genetic techniques (Table 1). Therefore, this study

For Review Only

combined modern genetic techniques and the reference genome available for this species to increase the power to detect differences among populations across a broad geographic range. The following questions were addressed: (1) how much genetic variation exists among wild populations of *B. oleracea* in the UK and Spain; (2) how are populations structured in the Atlantic region and how much differentiation exists between isolated populations; and (3) are there signals of local adaptation to the environment? The results provide insights into the utility of *B. oleracea* as a crop wild relative genetic resource for agriculture, as well as shed light on the most likely region of *B. oleracea* domestication.

Materials and Methods

Twenty-four populations of *B. oleracea* were chosen from the UK and Spain to cover both a latitudinal and longitudinal gradient of the Atlantic range for genetic analyses (Figure 1i & Table 2). French populations were not sampled here, but are the focus of a recent genetic analysis by Maggioni *et al.* (personal communication). Leaves were collected from four individual plants from each population for DNA extraction, as has been successfully applied to the study of population structure in wild relatives in the Brassicaceae (Buckley, Holub, Koch, Vergeer, & Mable, 2018). Nazareno, Bemmels, Dick, and Lohmann (2017) found that compared to "traditional" population genetic markers these smaller sample sizes are sufficient for various population statistics when large numbers of SNPs are available. The bedrock for each population was obtained from the British Geological Survey (BGS, 2018) and the Instituto Geológico y Minero de España (IGME, 2018). The first year a written record of a population exists was obtained for the UK populations from the Botanical Society of Britain & Ireland (BSBI, 2018). No equivalent records could be found for the Spanish populations.

Molecular methods

High molecular weight DNA was extracted from the leaves of 96 individuals from 24 populations (Table 2) using DNeasy Plant Mini Kits (QIAGEN, Hilden, Germany) and quantified using a Qubit 2.0 Fluorometer (ThermoFisher Scientific, Waltham, Massachusetts, U.S). Four samples from each population were sent for library preparation and sequencing at University of Exeter Sequencing Service. Double-digest

For Review Only

RADseq libraries were made using a modification of the method in Wu et al. (2016) that allowed NexteraXT indexes (Illumine Corp., USA) to be used for multiplexing samples. In addition, an RYRY spacer was inserted in the adapter 3' of the Illumina sequencing primer annealing site to provide additional complexity at the start of read 1 immediately before the Sac1 sticky end. For each sample 400 ng DNA was fully digested with Sac1 and Mse1 restriction endonucleases and purified using Ampure XP beads. Illumina compatible i5 adapters were designed to ligate to the at the AGCT-3' sticky end left after Sac1 digest, and Illumina compatible i7 adapters were designed to ligate to the 5'-TA overhangs remaining after Mse1 digest. Adapter-ligation excess adapters were removed using Ampure XP beads. DNA fragments were amplified by 12 cycles of indexing PCR, purified, size selected (inserts 330-670 bp) and validated using a Tapestation D1000 HS Screentape (Agilent Technologies Ltd). Libraries were equimolar pooled and the pool concentration was calculated after qPCR. Libraries were denatured, diluted and sequenced with 125bp paired-end reads on Illumina HiSeq 2500 using SBS High Output reagents v4 (Illumina Corp., USA).

Data processing

Reads were demultiplexed and trimmed to 100 bp using cutadapt (Martin, 2011). These were then cleaned and quality filtered using the *process_radtags* pipeline in Stacks v1.47 (Rochette & Catchen, 2017). Bowtie (v2; Langmead & Salzberg, 2012) and samtools (v1.9; Li et al., 2009) were used to align the reads to the *B. oleracea* reference genome (Liu et al., 2014). A catalogue of stacks was then created using *ref_map* (Stacks) with the default settings. The *populations* pipeline (Stacks) was used to filter the data, and calculate summary statistics. Three datasets were generated with different filtering parameters depending on the downstream analysis. Firstly, for dataset 1 (*within individuals*), which was used to estimate genetic diversity within individuals and in phylogenetic analyses, all individuals were filtered as a single population, and loci were retained if they had a minimum individual stack depth of five, a minimum minor allele frequency of 0.01, a maximum observed heterozygosity of 0.7 and were present in 60% of individuals. Secondly, dataset 2 was generated using the same filtering as dataset 1 but SNPs linked within each RAD locus were avoided by only retaining one SNP at random per locus; required for population structure analyses (Pritchard, Stephens, & Donnelly, 2000). Finally, for dataset

For Review Only

3 (*within populations*), which was used to calculate genetic distance between populations, individuals were assigned to their population of origin and loci were retained if present in 50% of the populations. This filtering was designed to reduce the inclusion of duplicate loci and balance the amount of missing data with the number of informative loci (Andrews et al., 2016). A minimum stack depth of five is higher than the default of two, but within the recommended range (Paris, Stevens, & Catchen, 2017), and helps to remove potential paralogues. Spurious SNPs were avoided by using a minor allele frequency of > 0.01 (Marandel et al., 2020), and the combination of a maximum observed heterozygosity of 0.7 (70% of the individuals or populations can be heterozygous for each locus) which are present in either 60% of individuals (datasets 1 and 2) or 50% of the populations (dataset 3) retains loci that have been successfully genotyped across individuals, but are not completely heterozygous. The summary statistics for each population were calculated in Stacks during the filtering of dataset 3 and included: the number of private alleles (PRI), expected heterozygosity (H_E), observed heterozygosity (H_O), percentage of polymorphic loci (%; Table 3), the inbreeding coefficient (F_{IS}) and nucleotide diversity (π; Supplementary information).

For Review Only

Data analyses

Clustering of samples within and between populations was investigated with dataset 1 using RAxML (v8.2; GTRCAT model and 1000 maximum likelihood bootstrap replicates; Stamatakis, 2014) and visualisation in SplitsTree4 (Huson & Bryant, 2005). To estimate the number of putative genetic clusters (K) and assess shared genetic ancestry, STRUCTURE (v2.3.4; Pritchard et al., 2000) was used with dataset 2, so as not to inflate sharing based on multiple SNPs within a RAD locus. A range of K values were tested (the number of populations successfully sequenced plus one; 1 – 21) using an admixture model that assumed correlated allele frequencies. For each K, five independent replicates of 100,000 MCMC repetitions, after a burn-in of 10,000 iterations, were run. The most likely K was selected using the log likelihoods and deltaK (Evanno, Regnaut, & Goudet, 2005). To see if there were significant differences between estimates of H_E and H_O, pairwise-ANOVAs were carried out in R version 3.4.0 (R Core Team, 2017) on estimates from dataset 3 based on variant sites alone and all sites. A genetic distance matrix was created using dataset 3, and the latitude and longitude of each population was

used to calculate a geographic distance matrix using 'Haversine' Great Circle Distance in the R package 'geosphere' (Hijmans, 2017). In addition, genetic and geographic matrices were created for Spanish and UK populations separately, alongside a temporal distance matrix for the year when each population was first recorded within the UK (first population record; Table 2). Mantel tests were carried out with 9999 replicates on the region-wide matrices and country matrices separately, to assess both the overall and within country isolation-by-distance. Mantel tests were also carried out on the UK specific matrices to investigate any relationship between the first population records and the genetic and geographic distances.

A subset of dataset 1 where the soil pH was known was used to investigate the relationship between soil pH and H_E – e.g., is a higher soil pH associated with lower genetic diversity? A linear model with soil pH as a predictor variable and H_E as a response variable was run on 21 individuals (across six populations) from four soil pH classes: Neutral (6.6 - 7.3), Slightly acidic (6.1 - 6.5), Moderately acidic (5.6 - 6.0) and Strongly acidic (5.0 - 5.5) based on USDA (1998).

In order to identify potential genotype-environment associations, redundancy analyses (RDA) were carried out using dataset 1 following Forester et al. (2018) with the R packages 'vegan' and 'pysch' (Oksanen et al., 2017; Revelle, 2018). The climate dataset was downloaded from the WorldClim database at a resolution of 4.5 km (Fick & Hijmans, 2017). This dataset is based on measurements made between 1970 – 2000. Therefore, it is assumed that any changes in climate will be consistent enough across the study gradient to maintain differences in the averages and variation between populations. The 19 climate variables available from WorldClim for our dataset were checked for pairwise correlations and the estimated variance inflation factor (VIF). Variables with correlations $> |0.7|$ and VIF > 10 were removed, leaving: 'Annual Mean Temperature', 'Mean Temperature of Wettest Quarter', 'Annual Precipitation' and 'Precipitation Seasonality'. Longitude was included as an additional predictor variable because it was weakly correlated with climatic variables. Those SNPs that had RDA loadings with q-values < 0.1 were considered outlier loci, and were compared to the annotated *B. oleracea* genome using Bedtools (v.2.17.0; Quinlan & Hall, 2010), followed by a search of the online resource 'Bolbase' (Yu et al., 2013) to investigate putative gene functions.

For Review Only

Results

Patterns of genetic diversity

A total of 115,746,909 reads from 76 individuals (20 populations; Table 2) were of sufficient quality and retained for down-stream analysis (average reads per individual: 1,522,986; range: 220,363 – 5,361,799; Supplementary Table 1). For four of the populations, no individuals were successfully sequenced and so these were not included in these analyses. On average 86.3% (range 82.5 - 88.6) of reads mapped to the reference genome (Supplementary Figure 1). Datasets 1 and 2 contained 42,517 and 13,352 SNPs, respectively, across 13,352 RAD-loci (Supplementary Table 2). There were 140,131 SNPs across 53,539 RAD-loci in dataset 3 (Supplementary Information).

Based on variable nucleotide sites only (Table 3), average estimates of genetic diversity (considering H_E) were lower than in the studies cited in Table 1; the average across populations was 0.120 among both UK (range 0.090 – 0.200) and Spanish (range 0.055 – 0.153) populations. Observed heterozygosity was consistently significantly (H_O $p < 0.001$) greater than H_E for all populations and average F_{IS} was similar in the two geographic regions (UK: average = 0.039, range = 0.001 to 0.084; Spain: average = 0.027, range = 0.025 to 0.031). There was thus no evidence of inbreeding (as expected given the genetically controlled self-incompatibility system) but heterozygosity excess was apparent in all populations. The Fortrose population contained 10-fold more private alleles compared to all other populations and had the highest values for both H_E and H_O. Values considering all sites were lower but did not change conclusions about relative patterns of diversity (Table 3).

Population structure

Based on the RAxML tree, the majority of individuals clustered by population, with the exceptions of: (i) two individuals that did not cluster with any population (one in San Juan de Gaxtelugatxe, Spain and one in St Aldehelm's Head, UK), and (ii) an individual from Fortrose (Scotland, UK) that clustered more closely with other Scottish populations than other individuals from Fortrose (Figure 1ii). The most likely number of genetic clusters from STRUCTURE analyses was K = 12. Most individuals were admixed, however, six of the UK populations (Fortrose, Auchmithie, Crail, Tynemouth, Whitby and Llantwit Major)

For Review Only

were dominated by a single genetic ancestry, and two individuals from Fortrose were distinct from both the third individual from Fortrose and all other samples (Figure 1iii). The dominant genetic ancestry seen in individuals from Whitby (UK) also dominated the genetic ancestry of individuals from Cabo de Peñas (Spain), and similarly, the dominant genetic ancestry seen in individuals from Tenby (UK) was most prevalent in individuals from San Juan de Gaxtelugatxe and Getaría (Spain). There were three potential regional clusters indicated by the RAxML tree and STRUCTURE analysis: (a) populations in Scotland; (b) populations closest to the Welsh-English border; and (c) populations in the Basque Country, Spain (excluding San Sebastian; Figure 1). However, the clustering of populations was not well resolved and these 'regional clusters' were not always the most geographically close populations (e.g., in cluster c, San Sebastian is closer to Getaría than San Juan de Gaxtelugaxte geographically but not genetically). No isolation-by-distance was predicted by the data either region-wide, or within Spanish or UK populations alone (Mantel test p-values = 0.474, 0.658 and 0.705, respectively). Furthermore, no relationship was found between the first record for each of UK populations (Table 2) with either geographic or genetic distance (Mantel test p-values = 0.114 and 0.933, respectively).

Environmental associations

Overall, environmental variables explained 2.3% (adjusted r-squared) of the variation in the SNPs using RDA analysis; the strongest association of genotype with the environment was with annual precipitation (Figure 3). This environmental variation was strong enough to be reflected in the clustering of individuals, including the genetically distinct individuals from Fortrose (UK; Figure 1iii). For example, across regions, west Scotland and the Basque country experienced the greatest amount of annual precipitation on average (Figure 2b), whereas the annual mean temperature was greater in the Basque country compared to west Scotland (Figure 2a). Individuals from populations in these regions separated from other populations in the same direction as annual precipitation, but in opposing directions in relation to annual mean temperature (Figure 3i). Individuals from Whitby (UK) appear to have experienced a colder, drier environment than the geographically closest population, Tynemouth (UK), which was also reflected in the RDA analysis. Linear modelling indicated a non-significant negative trend between genetic diversity (H_E, H_O & π) and soil pH (i.e. plant genetic diversity decreased as soil pH increased. Only H_E is shown

For Review Only

but the same relationship was found with H_O & π; Figure 4).

There were 2249 unique candidate SNPs associated with the predictor variables from the RDA analysis; the majority of these (1039) were most closely associated with 'Mean Temperature of Wettest Quarter', followed by 'Precipitation Seasonality' (349), 'Longitude' (333), 'Annual Precipitation' (269) and 'Annual Mean Temperature' (259). These were fairly evenly distributed across the genome with no indications of any single SNP with a large effect. A few SNPs that were more closely associated with annual precipitation had strong loadings along axis 1 in the direction of the annual precipitation vector (Figure 3(ii)). In total, 221 candidate SNPs mapped to unique genes in the *B. oleracea* reference genome, and of the top 18, six were annotated as part of the receptor-like kinase family (Table 4).

Discussion

The results presented here provide the first genome-wide estimates of genetic variation and population genetic structure of wild cabbages collected from across the UK and Spain. Although direct comparisons with cultivated species would be required to rigorously test hypotheses about origins of these populations, patterns of variation are consistent with recent linguistic and historical evidence (Maggioni, 2015; Maggioni et al., 2018) suggesting that the domestication of *B. oleracea* crops occurred in the Mediterranean, domesticates were moved by people across Europe, escaped and established wild populations in the Atlantic region. For example, there was no indication of isolation-by-distance from northern Scotland to Spain ($>$ 14° latitude), which might be expected if these plants were natural colonisers following common phylogeographic patterns (e.g., Sharbel, Haubold, & Mitchell-Olds, 2000). Furthermore, genetic ancestry and clustering analyses suggested that geographically distant populations may have similar genetic sources, and could therefore have been established by similar source cultivars. The consistent excess of heterozygotes across populations, combined with evidence for admixture from STRUCTURE analyses, suggests mixing between 'isolated' populations (Rousset & Raymond, 1995), which could be due to interbreeding between cultivated plants growing near the wild populations. This highlights the possibility of continued introgression between cultivated and wild plants. Despite the lack of geographic genetic population structuring, there were signals of local adaptation to different climates

based on RDA analyses. In addition, within population genetic diversity estimates were comparable to other studies (e.g., Christensen et al., 2011; Watson-Jones et al., 2006), and as Watson-Jones et al. (2006) found, lower genetic diversity estimates were associated with higher soil pH. Therefore, these wild populations could hold useful adaptive alleles for plant breeding, and a suitable approach to investigate traits of agricultural interest (e.g., drought tolerance) could be to choose populations based on their environment of origin. However, further sequencing of a range of cultivars from different geographic regions would be required to further test these hypotheses.

Patterns of Genetic Diversity

Although the magnitude of estimates of genetic diversity based on the ddRADseq data presented here were lower than in previous studies (see Table 1) using allozymes (Lanner-Herrera, Gustafeson, Filt, & Bryngelsson, 1996; Lázaro & Aguinagalde, 1998; Raybould et al., 1999), microsatellites (Raybould et al., 1999) or AFLPs (Watson-Jones et al., 2006; Christensen et al., 2011), patterns of variation within the UK and Spain were strikingly similar to one another. Most populations also showed a relatively consistent excess of heterozygosity. These similarities could provide evidence for relatively recent origins of populations in the two regions, but whether this was from feralisation of cultivars or natural differentiation after natural colonisation cannot be distinguished by the data. Although there has been an ongoing debate as to the origin of wild *B. oleracea* populations in the Atlantic region (Song, Osborn, & Williams, 1990; Allender et al., 2007; Maggioni, 2015), domestication of *B. oleracea* in the Mediterranean region has been suggested by other genetic, phenotypic and linguistic studies (Mitchell, 1976; Maggioni, 2015; Maggioni et al., 2018). The subsequent movement of *B. oleracea* cultivars across Europe could then have resulted in a much narrower bottleneck than the initial domestication bottleneck in the Mediterranean as it removed the chance of gene flow from the wild relatives they originated from (Kofsky, Zhang, & Song, 2018). Consistent with this hypothesis, although the putative Mediterranean progenitor species remains unknown, Allender et al. (2007) found much greater estimates of genetic diversity within potential progenitor species from the Mediterranean region than either previous genetic diversity estimates made in *B. oleracea* (e.g., Christensen et al., 2011; Watson-Jones et al., 2006) or in this study.

For Review Only

Population structure

Several of the analyses here suggest less population structuring than might be expected in such geographically distinct populations if natural range expansion followed by isolation occurred. In this dataset, since the first recorded population (Tenby in 1773), one to three new populations have been recorded every thirty years within the UK (Table 2). However, neither the date the UK populations were first recorded, nor the genetic distances between populations in the UK and Spain, had a geographical pattern (i.e. no isolation by distance). Furthermore, although the majority of individuals clustered by population and some regional clustering was seen (Figure 1), it would not be possible to predict whether two individuals from geographically close or geographically distant populations are more genetically similar to each other. For example, Fowey and Prussia Cove (UK populations), and West Looe and Cabo de Peñas (UK and Spanish populations respectively), clustered together and shared more genetic ancestry than Fowey and West Looe, which are the closest geographically. Although more sampling would be required to explicitly test it, the evidence here suggests that these plants have not colonised the Atlantic region following common phylogeographic patterns (e.g., Sharbel et al., 2000) and therefore is consistent with *B. oleracea* domestication occurring outside of the Atlantic region. This is in line with results from other genetic, phenotypic and linguistic studies, which suggest the Mediterranean region is the most likely location for *B. oleracea* domestication (Maggioni, 2015; Maggioni et al., 2018; Mitchell, 1976).

The genetic ancestry and clustering analyses hint that populations could have been established by escapees from different cultivars. The majority of individuals were assigned to multiple sources of genetic ancestry (Figure 1iii), however, there were also cases where one putative source dominated at the individual- and population-levels, which could be the overall genetic background from the original source cultivar. Interestingly, there were two distinct individuals from Fortrose (10-fold more private alleles than other populations; Table 3) with a source that was assigned to no other individuals. Due to the ease of interbreeding between cultivars (Allender et al., 2007), this could indicate that these two Fortrose individuals are recent escapees from a different source population (e.g., local gardens), which are yet to have mixed with other individuals within the population. Furthermore, the more recent record of the population at Fortrose (1968), and the lack of assignment to other populations, suggests that this genetic background could be from a cultivar that has not been grown for a long period of time or widely

For Review Only

around the Atlantic coastlines. The excess of heterozygotes (H_O was significantly greater than H_E) and the general mix of shared genetic ancestry across such a wide geographical area in distinct populations, could also be an indication of continued introgression into these wild populations from agricultural and horticultural sources. It would be interesting to identify popular cultivars in the local areas of these populations, including any changes in the preferred cultivars through time, to investigate patterns of introgression in more detail. Such direct comparisons with cultivars could identify the most likely founder of these populations.

Using chloroplast microsatellite DNA markers, Allender et al. (2007) found two haplotypes in *B. oleracea* around the coasts of the UK; out of sixteen populations, fourteen were C:01 and two were C:04. The two populations with the C:04 haplotype were in Tyne & Wear, in the northeast of England; in the current study, this area is represented by the Tynemouth and Whitby populations. In line with the rarity of the chloroplast haplotypes identified in this region in the previous study, these two populations clustered most closely with populations not sampled by Allender et al. (2007); Tynemouth clustered with Fortrose, Scotland, and Whitby with the Spanish population Cabo de Peñas. Based on this information, it might be expected that the chloroplast haplotypes of Fortrose and Cabo de Peñas would also be C:04. In addition, the C:01 haplotype found in the majority of the UK populations was also found in four other species of *Brassica* (Allender et al., 2007), suggesting either that this is the ancestral form or introgression between species. A combination of nuclear and chloroplast information could be useful for disentangling the population histories further, particularly in relation to identifying introgression.

Knowledge of the founding cultivars would be useful for both plant breeders and those interested in invasive species. It could provide insights into how different cultivars have adapted (and therefore may adapt in the future) to different environmental conditions, and could also be thought of as a way to compare invasion success within a species. *Brassica oleracea* lack the characteristics thought to be fundamental for establishment in novel locations (invasions; Funk et al., 2016), but perhaps amongst the huge phenotypic variation found within this species, some traits are more likely to lead to successful 'invasions' of particular cultivars compared to others. For example, a cultivated Danish kale was the most likely source for a wild population found in Denmark (based on AFLP markers; Christensen et al., 2011), and it could be that all the Atlantic populations were established by different kale cultivars.

Overall, populations of *B. oleracea* growing along Atlantic coasts would be an excellent study system to improve understanding of invasive species that are likely to harbour useful adaptive traits for agriculture.

While comparisons with published whole genome sequence data or other types of genotype by sequencing approaches (e.g., Stansell et al., 2018) for cultivated *B. oleracea* would be interesting to more explicitly test origins of the populations studied here, there are several issues with ddRAD data that would make this challenging and potentially hard to interpret. A benefit of ddRAD sequencing is the generation of discrete loci that are standardised to the same length. However, the resulting short sequence segments normally contain only one or a few SNPs, which does not allow accurate assignment of paralogs in highly duplicated and rearranged genomes such as found in the Brassicaceae (e.g., Schranz, Lysak, & Mitchell-Olds, 2006). Instead, filtering pipelines to allow population genetics analyses based on ddRAD data are designed to be conservative (Paris et al., 2017; Marandel et al., 2020). This filtering results in fewer loci retained, but it should reduce risks of including duplicates. In the current study, excess heterozygosity was observed consistently across populations, which could suggest historical introgression. Although we cannot completely rule out the influence of combining duplicates (Ilut, Nydam, & Hare, 2014), the highly consistent patterns of excess suggest that all populations would have been affected similarly, enabling interpretations of relative variation within and between populations. The admixture suggested by the STRUCTURE analyses also supports the role of introgression in the histories of the studied populations. However, mapping of the ddRAD reads to multiple reference genomes or to data generated based on different restriction enzymes would be more problematic.

For Review Only

Environmental associations

Despite the general lack of geographic clustering, there was evidence of local adaptation to the varying environments using redundancy analyses, particularly to annual precipitation (Figure 3). Although Watson-Jones et al. (2006) found some population structuring within the UK, the same result was not found in this study (i.e. no isolation-by-distance within the UK). Furthermore, no evidence of population structuring was found in the Spanish populations here, and Maggioni *et al.* (personal communication) found no evidence of population structuring in French Atlantic populations. These results could also be correlated with annual precipitation; perhaps the strong variation in annual precipitation in the UK (e.g.,

a strong west-east gradient) is causing more differentiation between these populations, whereas along the French range annual precipitation has a smaller gradient. One reason for the importance of annual precipitation other than water availability could be the influence of precipitation on soil pH. Soil pH is primarily determined by bedrock, but is also altered by precipitation through leaching of compounds such as calcium carbonate (Kinzel, 1983). Therefore, although slightly alkaline to neutral soils tend to form over limestone, secondary acidification can occur under higher precipitation regimes. The soil pH values recorded here ranged from neutral to strongly acidic (Figure 4). Furthermore, the bedrock of a large proportion of the populations used here (Table 2) differ from the limestone and chalk cliffs that wild *B. oleracea* are thought to be predominantly found on (Christensen et al., 2011). For those individuals where the soil pH was known, the same trend was found here as by Watson-Jones et al. (2006), with a decrease in plant genetic diversity as soil pH increased (Figure 4). For agriculture and horticulture, soil pH is an important consideration (Tilman, Balzer, Hill, & Befort, 2011). The change in plant genetic diversity suggests that soil pH is a strong selective pressure in the wild, causing an adaptive ecological bottleneck in locations where it is higher, resulting in lower genetic diversity. These indications of local adaptation despite a lack of population structure highlight environmental variables that could be investigated further in wild populations of *B. oleracea*, which regardless of their origin are surviving.

Alongside survival, a huge concern for food security related to climate change is the ability of crop plants to remain productive under rapidly changing environmental conditions (Lasky et al., 2015). Obtaining accurate phenotypic data for adaptive traits is a major barrier as we often do not know the combination of traits that underlie differences in fitness or how these vary with the environment (Kooyers, Greenlee, Colicchio, Oh, & Blackman, 2015). Although some traits will be locally adaptive due to large effect loci, the vast majority of adaptive traits are likely to have a polygenic basis (Rockman, 2012), particularly in the case of multi-trait phenotypes related to environmental gradients. Our results match these expectations, as no large effect loci were found; however, some were more significantly associated with the assessed environmental variation than others. The most likely assignment for six of the top 18 candidate genes was to the receptor-like kinase family (Table 4). This gene family underwent an expansion that is believed to be a plant-specific adaptation for pathogen defence (Afzal, Wood, & Lightfoot, 2008). Interestingly, Zhang et al. (2014) also found differences in genes related to plant defence

For Review Only

when investigating adaptations of rice (*Oryza* sp.) across four continents. These results highlight the fundamental importance of the immune system to fitness, and suggest that it could be related to environmental differences across different spatial scales. Given that immune system genes are among the best candidates for local adaptation, there is a potential connection between plant genetic diversity, soil pH and pathogens. It would be interesting to investigate whether less acidic soils host more pathogens, increasing the selective pressure on the plants and decreasing the plant genetic diversity in these soils. Overall, the impact of climate change on the spread of virulence of plant pathogens and herbivores, and the phenological mismatches that may occur between interacting species remain unknown (De Lucia, Nabity, Zavala, & Berenbaum, 2012; Fisher et al., 2012; Yang & Rudolf, 2010). What is clear is that plant defence will continue to be an important component of crop productivity, warranting further research.

Overall, the results presented here supported the hypothesis that wild populations of *B. oleracea* in the Atlantic region were established by plants from agricultural and/or horticultural sources. In addition, regardless of their origin, these wild populations are likely to contain useful genetic resources and should be considered as valuable populations of a crop wild relative to be investigated further.

Acknowledgements

EAM was funded by a University of Glasgow Lord Kelvin Adam Smith PhD studentship; UZI was funded by a NERC Independent Research Fellowship (NE/L011956); CAC is supported by the BBSRC (BB/P004202/1); KAM utilised equipment funded by the Wellcome Trust Institutional Strategic Support Fund (WT097835MF), Wellcome Trust Multi User Equipment Award (WT101650MA) and BBSRC LOLA award (BB/K003240/1). Part of the work was supported by a British Society for Plant Pathology summer studentship, and grants from the Botanical Research Fund, and the Blodwen Lloyd Bins trust funded through the Glasgow Natural History Society. None of the sponsors had any role in the study design, data collection, analysis, and interpretation or any aspects during the write up and publication of this work. We thank Danijela Dimitrijević and Deborah Davy for assistance in the field, and Dr Lorenzo Maggioni for useful discussions.

For Review Only

References

Afzal, A. J., Wood, A. J., & Lightfoot, D. A. (2008). Plant receptor-like serine threonine kinases: roles in signaling and plant defense. *Molecular Plant-Microbe Interactions*, *21*(5), 507–517.

Allender, C., Allainguillaume, J., Lynn, J., & King, G. J. (2007). Simple sequence repeats reveal uneven distribution of genetic diversity in chloroplast genomes of *Brassica oleracea* L. and (n= 9) wild relatives. *Theoretical and Applied Genetics*, *114*(4), 609–618.

Andrews, K. R., Good, J. M., Miller, M. R., Luikart, G., & Hohenlohe, P. A. (2016). Harnessing the power of RADseq for ecological and evolutionary genomics. *Nature Reviews Genetics*, *17*(2), 81–92.

BGS. (2018). *British Geological Survey.* http://www.bgs.ac.uk/. (Accessed: 2018-08-26)

BSBI. (2018). *Botanical Society of Britain & Ireland.* https://bsbi.org. (Accessed: 2018-08-26)

Buckley, J., Holub, E. B., Koch, M. A., Vergeer, P., & Mable, B. K. (2018). Restriction associated DNA-genotyping at multiple spatial scales in *Arabidopsis lyrata* reveals signatures of pathogen-mediated selection. *BMC Genomics*, *19*(1), 496.

Charlesworth, D., Vekemans, X., Castric, V., & Glémin, S. (2005). Plant self-incompatibility systems: a molecular evolutionary perspective. *New Phytologist*, *168*(1), 61–69.

Christensen, S., von Bothmer, R., Poulsen, G., Maggioni, L., Phillip, M., Andersen, B. A., & Jørgensen, R. B. (2011). AFLP analysis of genetic diversity in leafy kale (*Brassica oleracea* L. convar. *acephala* (DC.) Alef.) landraces, cultivars and wild populations in Europe. *Genetic Resources and Crop Evolution*, *58*(5), 657–666.

Conner, J. K. (2002). Genetic mechanisms of floral trait correlations in a natural population. *Nature*, *420*(6914), 407–410.

Darwin, C. R. (1859). *The origin of species.* London: John Murray.

De Lucia, E., Nabity, P., Zavala, J., & Berenbaum, M. (2012). Climate change: resetting plant-insect interactions. *Plant Physiology*, pp–112.

Diamond, J. (2002). Evolution, consequences and future of plant and animal domestication. *Nature*, *418*(6898), 700.

Euro+Med PlantBase. (2020). http://ww2.bgbm.org/EuroPlusMed/. (Accessed: 2020-05-23)

Evanno, G., Regnaut, S., & Goudet, J. (2005). Detecting the number of clusters of individuals using the software STRUCTURE: a simulation study. *Molecular Ecology*, *14*(8), 2611–2620.

Falconer, D. S., & Mackay, T. F. C. (1996). *Introduction to Quantitative Genetics* (4th ed.). Harlow, UK: Longman.

Fick, S. E., & Hijmans, R. J. (2017). WorldClim 2: new 1-km spatial resolution climate surfaces for global land areas. *International Journal of Climatology*, *37*(12), 4302–4315.

Fisher, M. C., Henk, D. A., Briggs, C. J., Brownstein, J. S., Madoff, L. C., McCraw, S. L., & Gurr, S. J. (2012). Emerging fungal threats to animal, plant and ecosystem health. *Nature*, *484*(7393), 186.

Forester, B. R., Lasky, J. R., Wagner, H. H., & Urban, D. L. (2018). Comparing methods for detecting multilocus adaptation with multivariate genotype–environment associations. *Molecular Ecology*, *27*(9), 2215–2233.

Funk, J. L., Standish, R. J., Stock, W. D., & Valladares, F. (2016). Plant functional traits of dominant native and invasive species in mediterranean-climate ecosystems. *Ecology*, *97*(1), 75–83.

Gao, Y., Yin, S., Wu, L., Dai, D., Wang, H., Liu, C., & Tang, L. (2017). Genetic diversity and structure of wild and cultivated *Amorphophallus paeoniifolius* populations in southwestern China as revealed by RAD-seq. *Scientific Reports*, *7*(1), 14183.

Gómez-Campo, C., & Prakash, S. (1999). Origin and domestication. In *Developments in plant genetics and breeding* (Vol. 4, pp. 33–58). Elsevier.

Henriksen, R., Gering, E., & Wright, D. (2018). Feralisation—The understudied counterpoint to domestication. In *Origin and evolution of biodiversity* (pp. 183–195). Springer.

Hijmans, R. J. (2017). geosphere: Spherical Trigonometry [Computer software manual]. Retrieved from https://CRAN.R-project.org/package=geosphere (R package version 1.5-7)

Hoisington, D., Khairallah, M., Reeves, T., Ribaut, J.-M., Skovmand, B., Taba, S., & Warburton, M. (1999). Plant genetic resources: What can they contribute toward increased crop productivity? *Proceedings of the National Academy of Sciences*, *96*(11), 5937–5943.

Huson, D. H., & Bryant, D. (2005). Application of phylogenetic networks in evolutionary studies. *Molecular Biology and Evolution*, *23*(2), 254–267.

IGME. (2018). *Instituto Geológico y Minero de España.* http://www.igme.es. (Accessed: 2018-08-26)

For Review Only

Ilut, D. C., Nydam, M. L., & Hare, M. P. (2014). Defining loci in restriction-based reduced representation genomic data from nonmodel species: sources of bias and diagnostics for optimal clustering. *BioMed Research International*, *2014*.

Kinzel, H. (1983). Influence of limestone, silicates and soil pH on vegetation. In *Physiological plant ecology iii* (pp. 201–244). Springer.

Kitashiba, H., & Nasrallah, J. B. (2014). Self-incompatibility in Brassicaceae crops: lessons for interspecific incompatibility. *Breeding Science*, *64*(1), 23–37.

Kofsky, J., Zhang, H., & Song, B.-H. (2018). The untapped genetic reservoir: The past, current, and future applications of the wild soybean (*Glycine soja*). *Frontiers in Plant Science*, *9*.

Kooyers, N. J., Greenlee, A. B., Colicchio, J. M., Oh, M., & Blackman, B. K. (2015). Replicate altitudinal clines reveal that evolutionary flexibility underlies adaptation to drought stress in annual *Mimulus guttatus*. *New Phytologist*, *206*(1), 152–165.

Langmead, B., & Salzberg, S. L. (2012). Fast gapped-read alignment with Bowtie 2. *Nature Methods*, *9*(4), 357.

Lanner-Herrera, C., Gustafeson, M., Filt, A., & Bryngelsson, T. (1996). Diversity in natural populations of wild *Brassica oleracea* as estimated by isozyme and RAPD analysis. *Genetic Resources and Crop Evolution*, *43*(1), 13–23.

Lasky, J. R., Upadhyaya, H. D., Ramu, P., Deshpande, S., Hash, C. T., Bonnette, J., ... others (2015). Genome-environment associations in sorghum landraces predict adaptive traits. *Science Advances*, *1*(6), e1400218.

Lázaro, A., & Aguinagalde, I. (1998). Genetic diversity in *Brassica oleracea* L.(Cruciferae) and wild relatives (2 n= 18) using isozymes. *Annals of Botany*, *82*(6), 821–828.

Li, H., Handsaker, B., Wysoker, A., Fennell, T., Ruan, J., Homer, N., ... Durbin, R. (2009). The sequence alignment/map format and SAMtools. *Bioinformatics*, *25*(16), 2078–2079.

Liu, S., Liu, Y., Yang, X., Tong, C., Edwards, D., Parkin, I. A., ... others (2014). The *Brassica oleracea* genome reveals the asymmetrical evolution of polyploid genomes. *Nature Communications*, *5*.

Maggioni, L. (2015). *Domestication of* Brassica oleracea *l.* (Unpublished doctoral dissertation). Swedish University of Agricultural Studies, Alnarp, Sweden.

Maggioni, L., von Bothmer, R., Poulsen, G., & Lipman, E. (2018). Domestication, diversity and use of *Brassica oleracea* L., based on ancient Greek and Latin texts. *Genetic Resources and Crop Evolution*, *65*(1), 137–159.

Marandel, F., Charrier, G., Lamy, J.-B., Le Cam, S., Lorance, P., & Trenkel, V. M. (2020). Estimating effective population size using RADseq: Effects of SNP selection and sample size. *Ecology and Evolution*, *10*(4), 1929–1937.

Martin, M. (2011, May). Cutadapt removes adapter sequences from high-throughput sequencing reads. *EMBnet.journal*, *17*(1), 10–12. Retrieved from `http://journal.embnet.org/index.php/embnetjournal/article/view/200` doi: http://dx.doi.org/10.14806/ej.17.1.200

Milla, R., Osborne, C. P., Turcotte, M. M., & Violle, C. (2015). Plant domestication through an ecological lens. *Trends in Ecology & Evolution*.

Mitchell, N. (1976). The Status of *Brassica oleracea* L. subsp. *oleracea* (wild cabbage) in the British Ilses. *Watsonia*, *11*, 97–103.

Nazareno, A. G., Bemmels, J. B., Dick, C. W., & Lohmann, L. G. (2017). Minimum sample sizes for population genomics: An empirical study from an Amazonian plant species. *Molecular Ecology Resources*, *17*(6), 1136–1147.

Nei, M. (1973). Analysis of gene diversity in subdivided populations. *Proceedings of the National Academy of Sciences*, *70*(12), 3321–3323.

Oksanen, J., Blanchet, F. G., Friendly, M., Kindt, R., Legendre, P., McGlinn, D., ... Wagner, H. (2017). vegan: Community Ecology Package [Computer software manual]. Retrieved from `https://CRAN.R-project.org/package=vegan` (R package version 2.4-3)

Paris, J. R., Stevens, J. R., & Catchen, J. M. (2017). Lost in parameter space: a road map for stacks. *Methods in Ecology and Evolution*, *8*(10), 1360–1373.

Prentis, P. J., Wilson, J. R., Dormontt, E. E., Richardson, D. M., & Lowe, A. J. (2008). Adaptive evolution in invasive species. *Trends in Plant Science*, *13*(6), 288–294.

Pritchard, J. K., Stephens, M., & Donnelly, P. (2000). Inference of population structure using multilocus genotype data. *Genetics*, *155*(2), 945–959.

For Review Only

Purugganan, M. D., & Fuller, D. Q. (2009). The nature of selection during plant domestication. *Nature*, *457*(7231), 843–848.

Quinlan, A. R., & Hall, I. M. (2010). BEDTools: a flexible suite of utilities for comparing genomic features. *Bioinformatics*, *26*(6), 841–842.

R Core Team. (2017). R: A language and environment for statistical computing [Computer software manual]. Vienna, Austria. Retrieved from `https://www.R-project.org/`

Rauf, S., Teixeira da Silva, J., Khan, A. A., & Naveed, A. (2010). Consequences of plant breeding on genetic diversity. *International Journal of Plant Breeding*, *4*(1), 1–21.

Raybould, A., Mogg, R., Clarke, R., Gliddon, C., & Gray, A. (1999). Variation and population structure at microsatellite and isozyme loci in wild cabbage (*Brassica oleracea* L.) in Dorset (UK). *Genetic Resources and Crop Evolution*, *46*(4), 351–360.

Revelle, W. (2018). psych: Procedures for Psychological, Psychometric, and Personality Research [Computer software manual]. Evanston, Illinois. Retrieved from `https://CRAN.R-project.org/package=psych` (R package version 1.8.10)

Rochette, N. C., & Catchen, J. M. (2017). Deriving genotypes from RAD-seq short-read data using Stacks. *Nature Protocols*, *12*(12), 2640.

Rockman, M. V. (2012). The QTN program and the alleles that matter for evolution: all that's gold does not glitter. *Evolution*, *66*(1), 1–17.

Rousset, F., & Raymond, M. (1995). Testing heterozygote excess and deficiency. *Genetics*, *140*(4), 1413–1419.

Sánchez-Yélamo, M. (2014). Characterisation of wild cabbage (*Brassica oleracea* L.) based on isoenzyme data. considerations on the current status of this taxon in Spain. *Genetic Resources and Crop Evolution*, *61*(7), 1295–1306.

Scheepens, J., Frei, E. S., Armbruster, G. F., & Stöcklin, J. (2012). Pollen dispersal and gene flow within and into a population of the alpine monocarpic plant *Campanula thyrsoides*. *Annals of Botany*, *110*(7), 1479–1488.

Schranz, M. E., Lysak, M. A., & Mitchell-Olds, T. (2006). The ABC's of comparative genomics in the Brassicaceae: building blocks of crucifer genomes. *Trends in Plant Science*, *11*(11), 535–542.

Sharbel, T. F., Haubold, B., & Mitchell-Olds, T. (2000). Genetic isolation by distance in *Arabidopsis thaliana*: biogeography and postglacial colonization of Europe. *Molecular Ecology*, *9*(12), 2109–2118.

Slatkin, M. (1987). Gene flow and the geographic structure of natural populations. *Science*, *236*(4803), 787–792.

Song, K., Osborn, T. C., & Williams, P. H. (1990). *Brassica* taxonomy based on nuclear restriction fragment length polymorphisms (RFLPs). *Theoretical and Applied Genetics*, *79*(4), 497–506.

Stamatakis, A. (2014). RAxML version 8: a tool for phylogenetic analysis and post-analysis of large phylogenies. *Bioinformatics*, *30*(9), 1312–1313.

Stansell, Z., Hyma, K., Fresnedo-Ramírez, J., Sun, Q., Mitchell, S., Björkman, T., & Hua, J. (2018). Genotyping-by-sequencing of *Brassica oleracea* vegetables reveals unique phylogenetic patterns, population structure and domestication footprints. *Horticulture Research*, *5*.

Tilman, D., Balzer, C., Hill, J., & Befort, B. L. (2011). Global food demand and the sustainable intensification of agriculture. *Proceedings of the National Academy of Sciences*, *108*(50), 20260–20264.

USDA, D. o. A. (1998). *Soil Quality Indicators: pH.* `https://www.nrcs.usda.gov/Internet/FSE_DOCUMENTS/nrcs142p2_052208.pdf`. (Accessed: 2016-04-20)

von Wettberg, E. J., Chang, P. L., Başdemir, F., Carrasquila-Garcia, N., Korbu, L. B., Moenga, S. M., ... others (2018). Ecology and genomics of an important crop wild relative as a prelude to agricultural innovation. *Nature Communications*, *9*(1), 649.

Walley, P. G., Teakle, G. R., Moore, J. D., Allender, C. J., Pink, D. A., Buchanan-Wollaston, V., & Barker, G. C. (2012). Developing genetic resources for pre-breeding in *Brassica oleracea* L.: an overview of the UK perspective. *Journal of Plant Biotechnology*, *39*(1), 62–68.

Watson-Jones, S., Maxted, N., & Ford-Lloyd, B. (2006). Population baseline data for monitoring genetic diversity loss for 2010: a case study for *Brassica* species in the UK. *Biological Conservation*, *132*(4), 490–499.

Wichmann, M. C., Alexander, M. J., Soons, M. B., Galsworthy, S., Dunne, L., Gould, R., ... Bullock, J. M. (2009). Human-mediated dispersal of seeds over long distances. *Proceedings of the Royal Society of London B: Biological Sciences*, *276*(1656), 523–532.

For Review Only

Wu, Z., Wang, B., Chen, X., Wu, J., King, G. J., Xiao, Y., & Liu, K. (2016). Evaluation of linkage disequilibrium pattern and association study on seed oil content in *Brassica napus* using ddRAD sequencing. *PloS One*, *11*(1), e0146383.
Xiao, Z., Han, F., Hu, Y., Xue, Y., Fang, Z., Yang, L., ... others (2019). Overcoming cabbage crossing incompatibility by the development and application of self-compatibility-QTL-specific markers and genome-wide background analysis. *Frontiers in Plant Science*, *10*, 189.
Yang, L. H., & Rudolf, V. (2010). Phenology, ontogeny and the effects of climate change on the timing of species interactions. *Ecology Letters*, *13*(1), 1–10.
Yousef, E. A., Mueller, T., Börner, A., & Schmid, K. J. (2018). Comparative analysis of genetic diversity and differentiation of cauliflower (*Brassica oleracea* var. botrytis) accessions from two *ex situ* genebanks. *PLoS One*, *13*(2), e0192062.
Yu, J., Zhao, M., Wang, X., Tong, C., Huang, S., Tehrim, S., ... Liu, S. (2013). Bolbase: a comprehensive genomics database for *Brassica oleracea*. *BMC Genomics*, *14*(1), 664.
Zhang, Q.-J., Zhu, T., Xia, E.-H., Shi, C., Liu, Y.-L., Zhang, Y., ... others (2014). Rapid diversification of five *Oryza* AA genomes associated with rice adaptation. *Proceedings of the National Academy of Sciences*, *111*(46), E4954–E4962.

Data Accessibility

The sequencing data and associated meta data are available on the European Nucleotide Archive under the study accession number: PRJEB38464 (http://www.ebi.ac.uk/ena/data/view/PRJEB38464)

Author Contributions

EAM and BKM conceived and proposed the study to the co-authors; EAM, BKM, CAC, and UZI then developed the study design. BKM, CAC, and UZI obtained the main funding award. EAM and EAK collected the data. EAM carried out statistical analysis, and wrote the paper. KAM carried out the double-digest RAD-sequencing. All authors reviewed the final version of the manuscript and agreed to its content before submission.

For Review Only

For Review Only

Table 1: Estimates of genetic diversity within wild *B. oleracea* populations from previous studies using different molecular markers. H_E is expected heterozygosity estimated using Nei's gene diversity (Nei, 1973).

Study	Molecular marker	H_E	Populations
Lanner-Herrera et al. (1996)	Isozymes	0.10 – 0.56	France, Spain, UK
Lázaro and Aguinagalde (1998)	Isozymes	0.26 – 0.30	France, Spain, UK
Raybould et al. (1999)	Isozymes	0.40 (0.18 – 0.41)†	UK
Raybould et al. (1999)	Microsatellites	0.36 (0.21 – 0.33)†	UK
Watson-Jones et al. (2006)	AFLPs	0.19 – 0.33	UK
Christensen et al. (2011)	AFLPs	0.23, 0.20	Spain, UK
Maggioni *et al.* (pers. comm. 2019)	AFLPs	0.25	France

† – pooled population H_E with the range of estimates from individual populations shown in brackets.

Table 2: A summary of the natural populations of *B. oleracea* used in this study, including: the bedrock, the first time the population was recorded, the number of individuals sequenced, and the number of individuals included in down-stream analyses.

Region	Population	Bedrock†	First population record‡	Number sequenced	Number included§
ES	Auchmithie	Red basic sandstone	1913	4	4
ES	Crail	Sandstone & limestone	1840	4	4
ES	Fortrose	Sandstone	1968	4	3
WS	Kildonan Castle	Sandstone & limestone	1987	4	4
NEE	Tynemouth	Sandstone & limestone	1805	4	4
NEE	Staithes	Shale & sandstone	1831	4	–
NEE	Whitby	Shale	1906	4	4
NW	Little Orme	Limestone	1895	4	–
SW	Tenby	Siltstone & sandstone	1773	4	4
SW	Llantwit Major	Limestone	1850	4	4
SWE	Prussia Cove	Slate, shale & siltstone	1871	4	4
SWE	Fowey	Shale & siltstone	1805	4	4
SWE	West Looe	Siltstone & sandstone	1971	4	2
SWE	St. Aldhelm's Head	Limestone	1933	4	4
A	Cudillero	Slate & sandstone		4	4
A	Playa de Xágo	Sandstone & Dolomite		4	4
A	Cabo de Peñas	Slate & quartzite		4	4
A	Playas de Viodo	Slate & shale		4	4
A	Tazones	Dolomite & limestone		4	–
C	Playa Pedrero	Quartzites		4	4
C	La Franca	Quartzites		4	–
BC	San Juan de Gaxtelugatxe	Limestone		4	4
BC	Getaría	Limestone		4	4
BC	San Sebastian	Calcareous sandstone		4	3
			**Total:**	96	76

† data obtained from the British Geological Survey (`https://www.bgs.ac.uk`) and the Instituto Geológico y Minero de España (`http://www.igme.es`). Region codes: ES – East Scotland, WS – West Scotland, NEE – North-eastern England, NW – North Wales, SW – South Wales, SWE – South-western England, A – Asturias Spain, C – Cantabrica Spain, BC – Basque Country Spain. ‡ data obtained from the Botanical Society of Britain & Ireland (`https://bsbi.org`). § indicates where data was lost in quality filtering of sequences and not included in down-stream analyses.

For Review Only

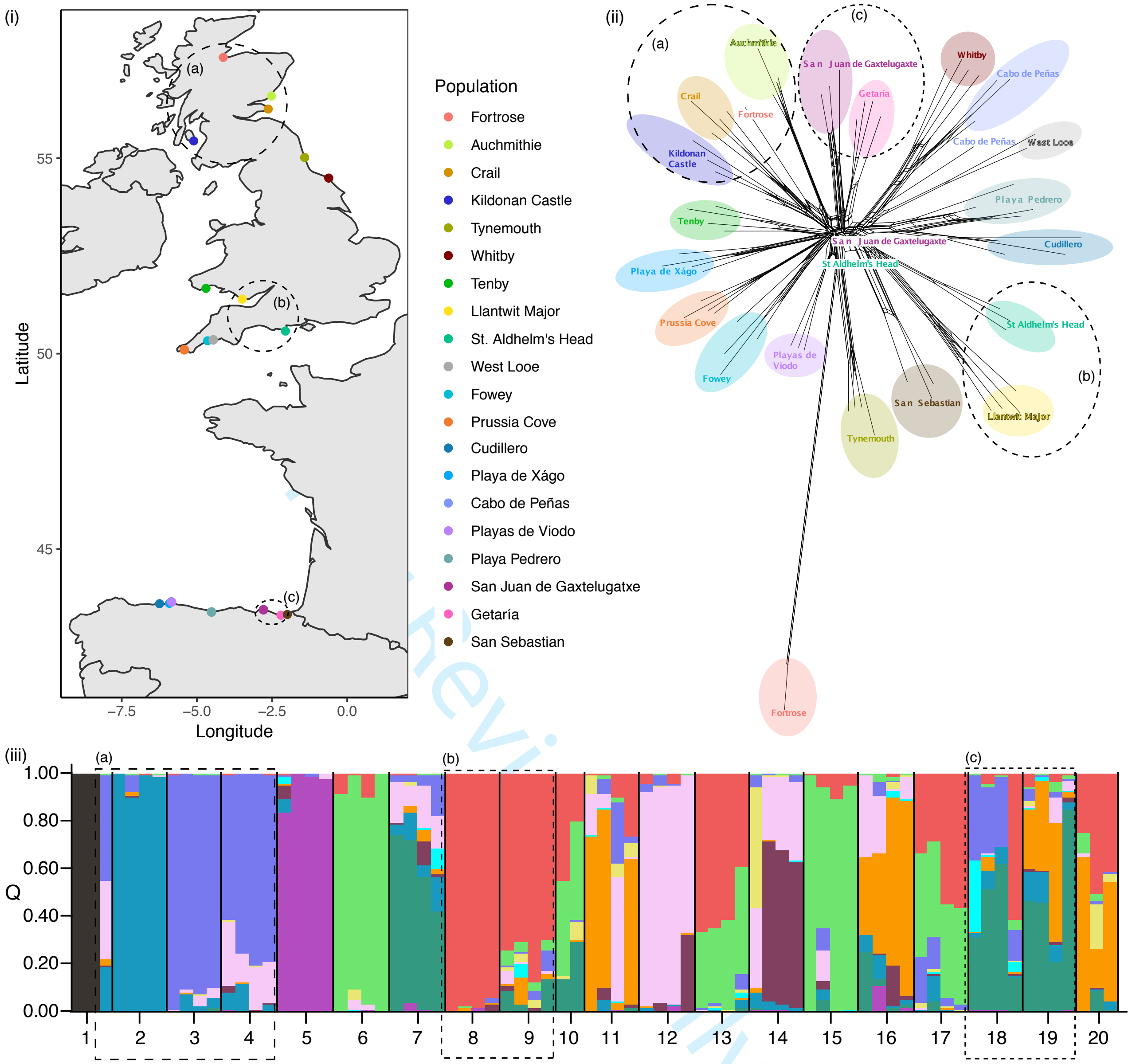

Figure 1: Population structuring of wild populations of *B. oleracea*. (i) Location of the populations considered here. (ii) Clustering of samples from RAxML (v8.2; GTRCAT model and 1000 maximum likelihood bootstrap replicates), visualised in SplitsTree4. (iii) STRUCTURE plot illustrating shared genetic ancestry for K = 12, ordered by population: 1 – Fortrose; 2 – Auchmithie; 3 – Crail; 4 – Kildonan Castle; 5 – Tynemouth; 6 – Whitby; 7 – Tenby; 8 – Llantwit Major; 9 – St. Aldhelm's Head; 10 – West Looe; 11 – Fowey; 12 – Prussia Cova; 13 – Cudillero; 14 – Playa de Xágo; 15 – Cabo de Peñas; 16 – Playas de Viodo; 17 – Playa Pedrero; 18 – San Juan de Gaxtelugatxe; 19 – Getaría; 20 – San Sebastian. Across the figures the same colours and numbering is used for each population. The dashed lines and letters indicate some clustering: (a) populations in Scotland; (b) populations closest to the Welsh-English border; and (c) populations in the Basque Country, Spain (excluding San Sebastian).

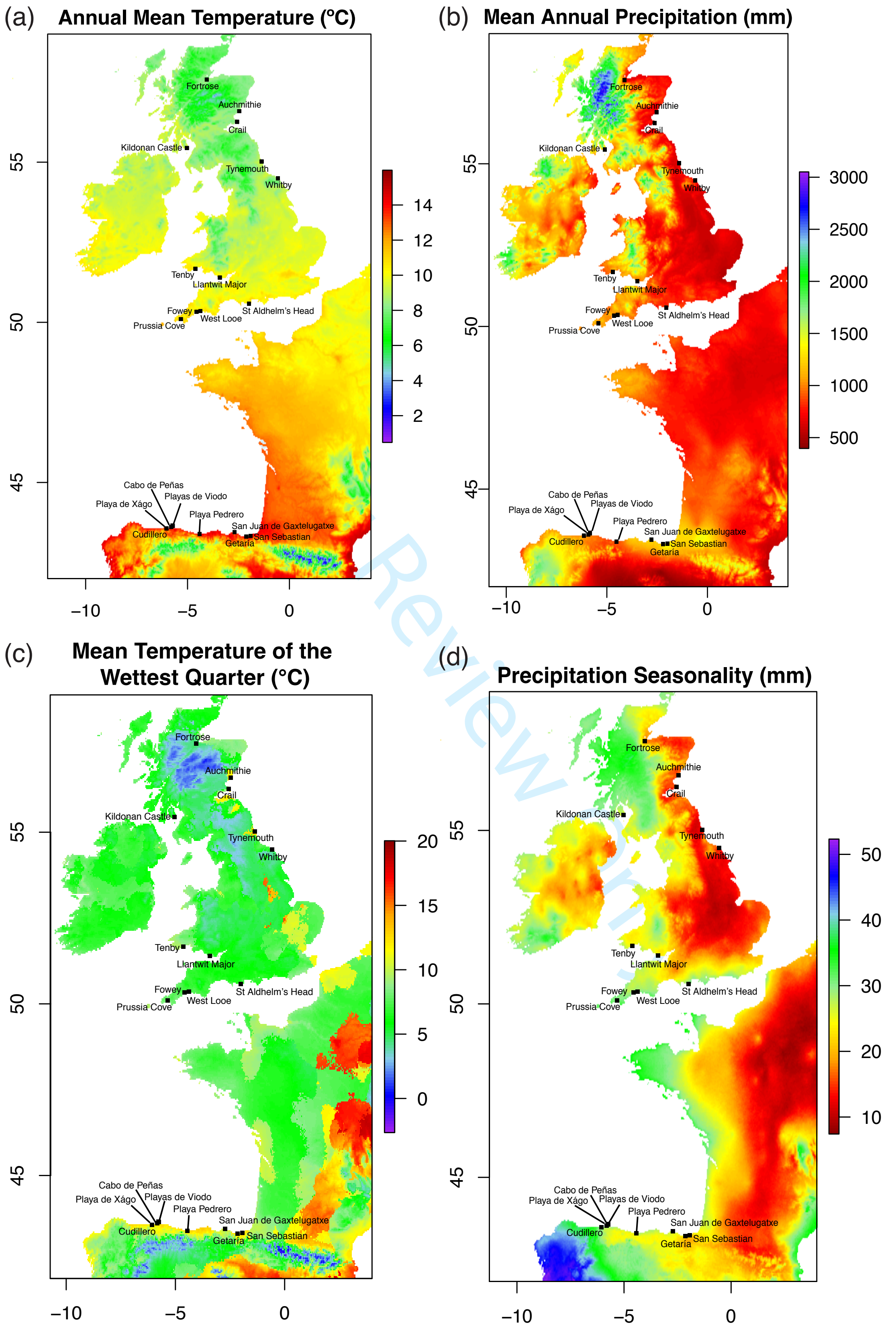

Figure 2: The distribution of sampled populations in relation to various climate variables: (a) annual mean temperature (℃); (b) mean annual precipitation (mm); (c) mean temperature of wettest quarter (℃); (d) precipitation seasonality (mm). These are averages between 1970 – 2000 obtained from the WorldClim database (Fick & Hijmans, 2017).

Table 3: Summary statistics of within *B. oleracea* population genetic diversity based on both variant nucleotide sites alone (var) and all sites (all) from dataset 1, showing: the number of individuals (N), the number of private alleles (PRI), expected heterozygosity (H_E), observed heterozygosity (H_O) and percentage of polymorphic loci (%).

**Region**†	**Population**	**N**	**PRI**	H_E		H_O		**%**
				Var	*All*	*Var*	*All*	*All*
ES	Authmithie	4	1683	0.1043	0.0012	0.1202	0.0014	33.0
ES	Crail	4	1727	0.1327	0.0019	0.1267	0.0018	52.8
ES	Fortrose	3	12951	0.2006	0.0032	0.1962	0.0031	76.4
WS	Kildonan Castle	4	1014	0.0903	0.0014	0.0944	0.0014	40.8
NEE	Tynemouth	4	1476	0.1023	0.0013	0.0881	0.0011	36.4
NEE	Whitby	4	1573	0.1200	0.0020	0.1184	0.0020	56.7
SW	Tenby	4	1568	0.1227	0.0014	0.1153	0.0013	40.5
SW	Llantwit Major	4	2073	0.1390	0.0023	0.1231	0.0022	66.2
SWE	Prussia Cove	4	1454	0.1019	0.0016	0.1064	0.0017	45.5
SWE	Fowey	4	1137	0.1126	0.0018	0.1083	0.0017	53.4
SWE	West Looe	2	1412	0.1150	0.0011	0.1328	0.0013	27.1
SWE	St. Aldhelm's Head	4	2470	0.1486	0.0014	0.1676	0.0016	39.4
A	Cudillero	4	716	0.0918	0.0015	0.0938	0.0016	44.3
A	Playa de Xágo	4	1583	0.1140	0.0012	0.1191	0.0012	33.4
A	Cabo de Peñas	4	698	0.0933	0.0015	0.0910	0.0014	42.5
A	Playas de Viodo	4	503	0.0545	0.0004	0.0580	0.0004	11.2
C	Playa Pedrero	4	1741	0.1313	0.0014	0.1408	0.0015	38.5
BC	San Juan de Gaxtelugatxe	4	2608	0.1423	0.0012	0.1471	0.0012	34.0
BC	Getaría	4	1550	0.1280	0.0021	0.1391	0.0023	59.8
BC	San Sebastian	3	2516	0.1530	0.0023	0.1538	0.0023	61.4

†Region codes: ES – East Scotland, WS – West Scotland, NEE – North-eastern England, SW – South Wales, SWE – South-western England, A – Asturias Spain, C – Cantabrica Spain, BC – Basque Country Spain.

For Review Only

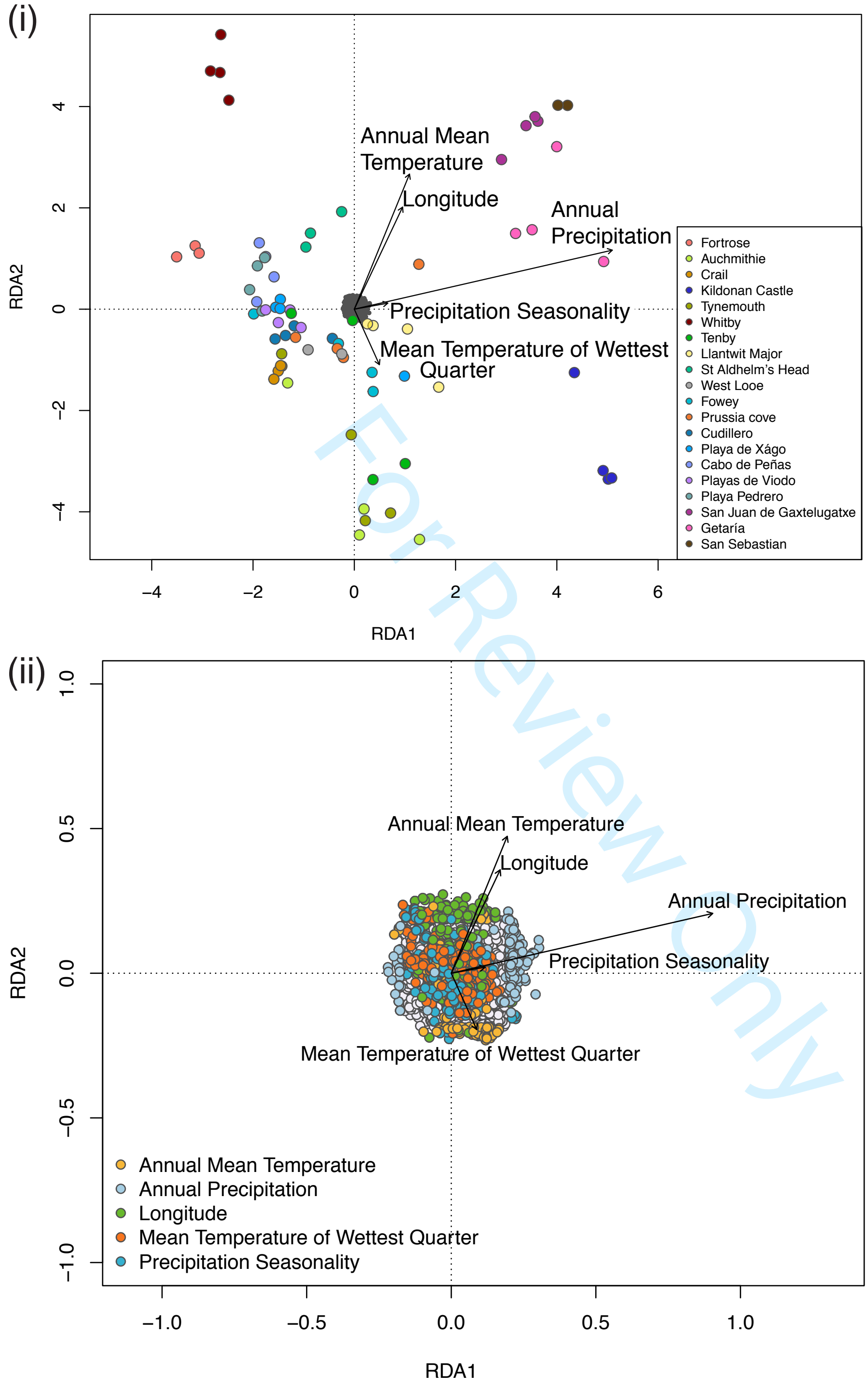

For Review Only

Figure 3: (i) Redundancy analysis (RDA) ordination plot of the association between *B. oleracea* individuals (coloured points) and SNPs (dark grey points), with environmental variables. The different colours indicate which population each individual was from. (ii) RDA ordination plot of the SNPs alone, coloured for the environmental variable with which they were most strongly associated. For both (i) & (ii) the arrows indicate the environmental predictors and the strength of the association.

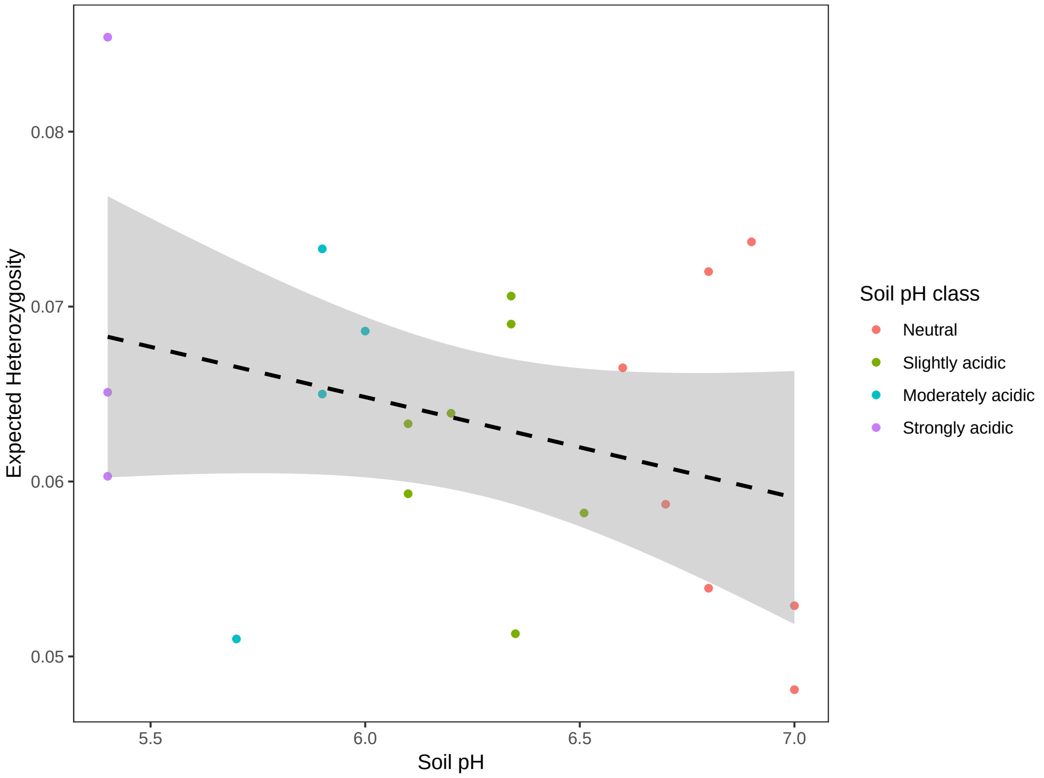

Figure 4: The relationship between expected heterozygosity and soil pH for 21 individuals from four soil pH classes categorised into: Neutral (6.6 - 7.3), Slightly acidic (6.1 - 6.5), Moderately acidic (5.6- 6.0) and Strongly acidic (5.0 - 5.5) based on USDA (1998). A linear model was used to fit a regression line (dashed black line), the standard error is shown in grey, p-value $>$ 0.05.

Table 4: The top 18 candidate SNPs that mapped to unique genes in the *B. oleracea* reference genome and their annotations from 'Bolbase' (Yu et al., 2013).

Chromosome	Location	Identity	X	Bolbase gene name	Potential protein	Function
C09	32879582	1	-	Bol019890	Ribonucleotide reductase-related	Fatty acid metabolic process, creation of DNA from RNA
C04	39737611	0.999979	-	Bol021601	Unknown	
C09	8499546	1	+	Bol032146	Basic helix-loop-helix dimerisation region	Nucleus transcription regulation
C07	43014116	1	-	Bol042101	Toll-Interleukin receptor	Signal transduction, immune response, disease resistance
C02	233586	1	+	Bol012817	Laccase/multicopper oxidase	Copper ion binding, metabolic process, maybe formation and degradation of lignin
C04	22051514	0.999656	+	Bol044300	Protein kinase - serine/threonine	Protein kinase activity, signalling, plant defence
C03	29308196	0.472347	-	Bol012462	PIK-related kinase	Binding and DNA repair
C03	48963472	0.99438	+	Bol029900	Protein kinase	Protein kinase activity, signalling, plant defence
C04	28456859	0.999661	-	Bol009961	Cystathionine beta-synthase	Vitamin B6 pathway?
C03	9456274	1	-	Bol005573	Unknown	
C05	2317477	0.580051	-	Bol041075	Pentotricopeptide repeat	Often essential in mitochondria
C04	35972614	0.304057	+	Bol037830	Bacterial transferase haxapeptide repeat	Binding and transferase activity
C04	35104965	0.996501	+	Bol037950	Cyclin-like F-box	Growth and development
C03	2461137	0.999261	-	Bol034275	Serine/threonine-protein kinase	Signalling, plant defence
C02	233586	0.168963	-	Bol012816	Serine/threonine-protein kinase	Signalling, plant defence
C01	11164295	0.999978	+	Bol039465	Initiation factor eIF-4 gamma, MA3	
C01	11431159	1	+	Bol039505	Heat shock protein Hsp20	
C01	12106862	0.918256	-	Bol039585	F-box associated	

For Review Only

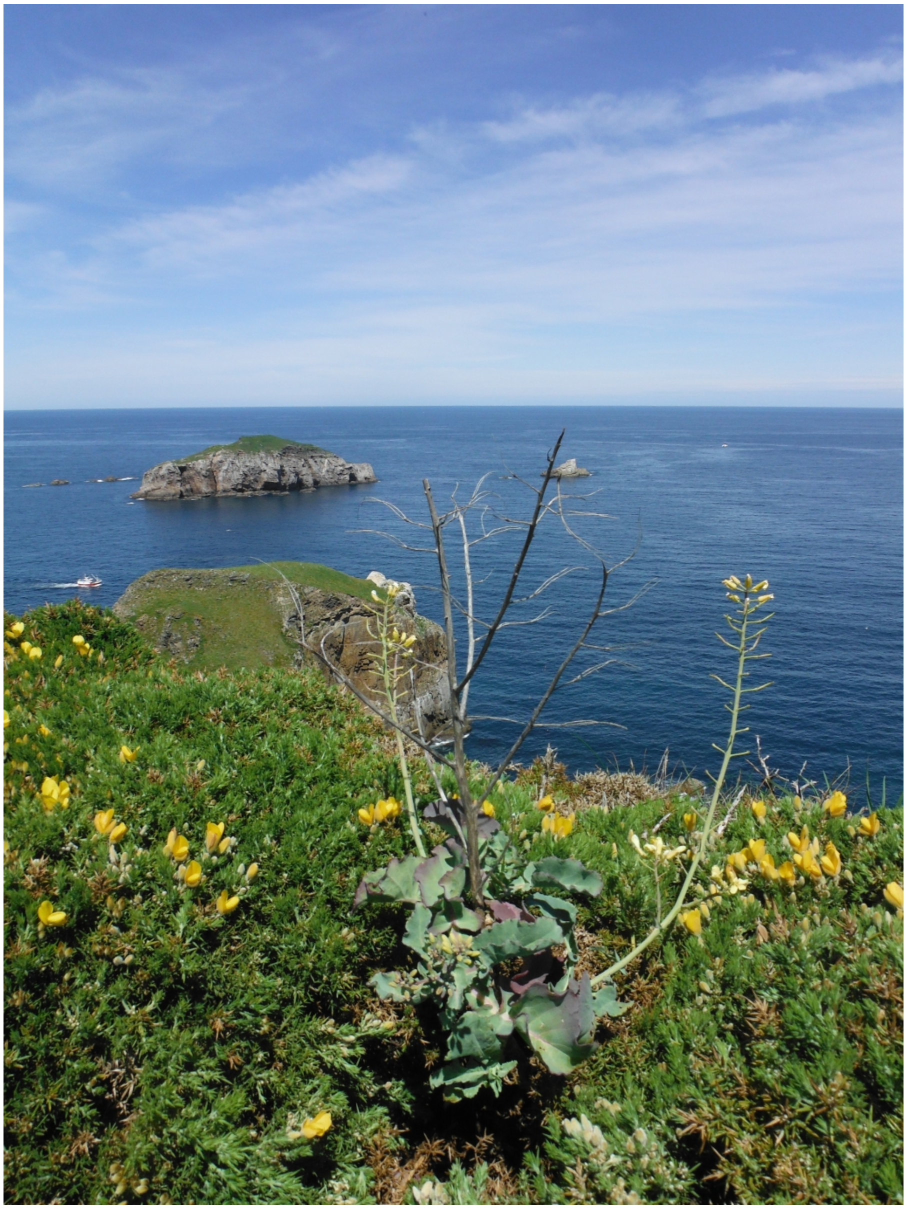

1143x1524mm (72 x 72 DPI)

Mittell_et_al 2020 Supplementary Information output from populations in stacks for the three datasets that were analysed in the manuscript.
Dataset 1 -- Within individuals. All individuals analysed together as one population.
Dataset 2 -- As dataset 1, but only one SNP was retained randomly per RAD-locus.
Dataset 3 -- Within populations. Individuals were assigned to the population they were sampled from.

Populations log for dataset 1

Distribution of population loci.
Distribution of valid loci matched to catalog locus.

# Valid samples at locus	Count
1	313602
2	65350
3	18307
4	8690
5	5498
6	4104
7	3142
8	2794
9	2283
10	2055
11	1949
12	1761
13	1652
14	1547
15	1455
16	1439
17	1342
18	1296
19	1283
20	1190
21	1229
22	1175
23	1156
24	1093
25	1088
26	1094
27	1030
28	1003
29	1016
30	960
31	967
32	918
33	950
34	990

For Review Only

35	977
36	933
37	830
38	859
39	910
40	898
41	838
42	848
43	830
44	852
45	869
46	868
47	804
48	817
49	888
50	827
51	849
52	880
53	778
54	812
55	891
56	838
57	873
58	840
59	831
60	906
61	947
62	958
63	952
64	913
65	1069
66	1128
67	1178
68	1243
69	1275
70	1335
71	1237
72	1163
73	998
74	626
75	400
76	326

Distribution of confounded loci at catalog locus.

# Confounded samples at locus	Count
0	493502

Distribution of missing loci at catalog loci.

For Review Only

# Absent samples at locus	Count
0	493502
Distribution of population loci after applying locus constraints.
Distribution of valid loci matched to catalog locus.
# Valid samples at locus	Count
45	671
46	710
47	735
48	703
49	681
50	721
51	713
52	640
53	677
54	681
55	737
56	711
57	722
58	721
59	768
60	858
61	750
62	765
63	691
64	699
65	667
66	582
67	516
68	433
69	305
70	241
71	171
72	140
73	120
74	115
75	151
76	145
Distribution of confounded loci at catalog locus.
# Confounded samples at locus	Count
0	17940
Distribution of missing loci at catalog loci.
# Absent samples at locus	Count
0	309
1	282
2	352
3	547

For Review Only

4	847
5	1123
6	1390
7	1588
8	1603
9	1763
10	1710
11	1501
12	1278
13	1122
14	858
15	598
16	425
17	289
18	173
19	96
20	51
21	21
22	9
23	4
25	1

Population 1 contained 0 incompatible loci -- more than two alleles present.
Population 2 contained 0 incompatible loci -- more than two alleles present.
Population 3 contained 0 incompatible loci -- more than two alleles present.
Population 4 contained 0 incompatible loci -- more than two alleles present.
Population 5 contained 0 incompatible loci -- more than two alleles present.
Population 6 contained 0 incompatible loci -- more than two alleles present.
Population 7 contained 0 incompatible loci -- more than two alleles present.
Population 8 contained 0 incompatible loci -- more than two alleles present.
Population 9 contained 0 incompatible loci -- more than two alleles present.
Population 10 contained 0 incompatible loci -- more than two alleles present.
Population 11 contained 0 incompatible loci -- more than two alleles present.
Population 12 contained 0 incompatible loci -- more than two alleles present.
Population 13 contained 0 incompatible loci -- more than two alleles present.
Population 14 contained 0 incompatible loci -- more than two alleles present.
Population 15 contained 0 incompatible loci -- more than two alleles present.
Population 16 contained 0 incompatible loci -- more than two alleles present.
Population 17 contained 0 incompatible loci -- more than two alleles present.
Population 18 contained 0 incompatible loci -- more than two alleles present.
Population 19 contained 0 incompatible loci -- more than two alleles present.
Population 20 contained 0 incompatible loci -- more than two alleles present.
Population 21 contained 0 incompatible loci -- more than two alleles present.
Population 22 contained 0 incompatible loci -- more than two alleles present.
Population 25 contained 0 incompatible loci -- more than two alleles present.
Population 26 contained 0 incompatible loci -- more than two alleles present.
Population 30 contained 0 incompatible loci -- more than two alleles present.

For Review Only

Population 31 contained 0 incompatible loci -- more than two alleles present.
Population 32 contained 0 incompatible loci -- more than two alleles present.
Population 33 contained 0 incompatible loci -- more than two alleles present.
Population 34 contained 0 incompatible loci -- more than two alleles present.
Population 35 contained 0 incompatible loci -- more than two alleles present.
Population 36 contained 0 incompatible loci -- more than two alleles present.
Population 37 contained 0 incompatible loci -- more than two alleles present.
Population 38 contained 0 incompatible loci -- more than two alleles present.
Population 39 contained 0 incompatible loci -- more than two alleles present.
Population 40 contained 0 incompatible loci -- more than two alleles present.
Population 41 contained 0 incompatible loci -- more than two alleles present.
Population 45 contained 0 incompatible loci -- more than two alleles present.
Population 46 contained 0 incompatible loci -- more than two alleles present.
Population 47 contained 0 incompatible loci -- more than two alleles present.
Population 48 contained 0 incompatible loci -- more than two alleles present.
Population 49 contained 0 incompatible loci -- more than two alleles present.
Population 50 contained 0 incompatible loci -- more than two alleles present.
Population 51 contained 0 incompatible loci -- more than two alleles present.
Population 52 contained 0 incompatible loci -- more than two alleles present.
Population 53 contained 0 incompatible loci -- more than two alleles present.
Population 54 contained 0 incompatible loci -- more than two alleles present.
Population 55 contained 0 incompatible loci -- more than two alleles present.
Population 56 contained 0 incompatible loci -- more than two alleles present.
Population 57 contained 0 incompatible loci -- more than two alleles present.
Population 58 contained 0 incompatible loci -- more than two alleles present.
Population 59 contained 0 incompatible loci -- more than two alleles present.
Population 60 contained 0 incompatible loci -- more than two alleles present.
Population 61 contained 0 incompatible loci -- more than two alleles present.
Population 62 contained 0 incompatible loci -- more than two alleles present.
Population 63 contained 0 incompatible loci -- more than two alleles present.
Population 64 contained 0 incompatible loci -- more than two alleles present.
Population 65 contained 0 incompatible loci -- more than two alleles present.
Population 66 contained 0 incompatible loci -- more than two alleles present.
Population 68 contained 0 incompatible loci -- more than two alleles present.
Population 69 contained 0 incompatible loci -- more than two alleles present.
Population 70 contained 0 incompatible loci -- more than two alleles present.
Population 71 contained 0 incompatible loci -- more than two alleles present.
Population 72 contained 0 incompatible loci -- more than two alleles present.
Population 73 contained 0 incompatible loci -- more than two alleles present.
Population 74 contained 0 incompatible loci -- more than two alleles present.
Population 75 contained 0 incompatible loci -- more than two alleles present.
Population 76 contained 0 incompatible loci -- more than two alleles present.
Population 77 contained 0 incompatible loci -- more than two alleles present.
Population 86 contained 0 incompatible loci -- more than two alleles present.
Population 87 contained 0 incompatible loci -- more than two alleles present.
Population 88 contained 0 incompatible loci -- more than two alleles present.

For Review Only

Population 89 contained 0 incompatible loci -- more than two alleles present.
Population 90 contained 0 incompatible loci -- more than two alleles present.
Population 91 contained 0 incompatible loci -- more than two alleles present.
Population 92 contained 0 incompatible loci -- more than two alleles present.
Population 93 contained 0 incompatible loci -- more than two alleles present.
Population 1 contained 0 incompatible loci -- more than two alleles present.
Population 2 contained 0 incompatible loci -- more than two alleles present.
Population 3 contained 0 incompatible loci -- more than two alleles present.
Population 4 contained 0 incompatible loci -- more than two alleles present.
Population 5 contained 0 incompatible loci -- more than two alleles present.
Population 6 contained 0 incompatible loci -- more than two alleles present.
Population 7 contained 0 incompatible loci -- more than two alleles present.
Population 8 contained 0 incompatible loci -- more than two alleles present.
Population 9 contained 0 incompatible loci -- more than two alleles present.
Population 10 contained 0 incompatible loci -- more than two alleles present.
Population 11 contained 0 incompatible loci -- more than two alleles present.
Population 12 contained 0 incompatible loci -- more than two alleles present.
Population 13 contained 0 incompatible loci -- more than two alleles present.
Population 14 contained 0 incompatible loci -- more than two alleles present.
Population 15 contained 0 incompatible loci -- more than two alleles present.
Population 16 contained 0 incompatible loci -- more than two alleles present.
Population 17 contained 0 incompatible loci -- more than two alleles present.
Population 18 contained 0 incompatible loci -- more than two alleles present.
Population 19 contained 0 incompatible loci -- more than two alleles present.
Population 20 contained 0 incompatible loci -- more than two alleles present.
Population 21 contained 0 incompatible loci -- more than two alleles present.
Population 22 contained 0 incompatible loci -- more than two alleles present.
Population 25 contained 0 incompatible loci -- more than two alleles present.
Population 26 contained 0 incompatible loci -- more than two alleles present.
Population 30 contained 0 incompatible loci -- more than two alleles present.
Population 31 contained 0 incompatible loci -- more than two alleles present.
Population 32 contained 0 incompatible loci -- more than two alleles present.
Population 33 contained 0 incompatible loci -- more than two alleles present.
Population 34 contained 0 incompatible loci -- more than two alleles present.
Population 35 contained 0 incompatible loci -- more than two alleles present.
Population 36 contained 0 incompatible loci -- more than two alleles present.
Population 37 contained 0 incompatible loci -- more than two alleles present.
Population 38 contained 0 incompatible loci -- more than two alleles present.
Population 39 contained 0 incompatible loci -- more than two alleles present.
Population 40 contained 0 incompatible loci -- more than two alleles present.
Population 41 contained 0 incompatible loci -- more than two alleles present.
Population 45 contained 0 incompatible loci -- more than two alleles present.
Population 46 contained 0 incompatible loci -- more than two alleles present.
Population 47 contained 0 incompatible loci -- more than two alleles present.
Population 48 contained 0 incompatible loci -- more than two alleles present.
Population 49 contained 0 incompatible loci -- more than two alleles present.

For Review Only

Population 50 contained 0 incompatible loci -- more than two alleles present.
Population 51 contained 0 incompatible loci -- more than two alleles present.
Population 52 contained 0 incompatible loci -- more than two alleles present.
Population 53 contained 0 incompatible loci -- more than two alleles present.
Population 54 contained 0 incompatible loci -- more than two alleles present.
Population 55 contained 0 incompatible loci -- more than two alleles present.
Population 56 contained 0 incompatible loci -- more than two alleles present.
Population 57 contained 0 incompatible loci -- more than two alleles present.
Population 58 contained 0 incompatible loci -- more than two alleles present.
Population 59 contained 0 incompatible loci -- more than two alleles present.
Population 60 contained 0 incompatible loci -- more than two alleles present.
Population 61 contained 0 incompatible loci -- more than two alleles present.
Population 62 contained 0 incompatible loci -- more than two alleles present.
Population 63 contained 0 incompatible loci -- more than two alleles present.
Population 64 contained 0 incompatible loci -- more than two alleles present.
Population 65 contained 0 incompatible loci -- more than two alleles present.
Population 66 contained 0 incompatible loci -- more than two alleles present.
Population 68 contained 0 incompatible loci -- more than two alleles present.
Population 69 contained 0 incompatible loci -- more than two alleles present.
Population 70 contained 0 incompatible loci -- more than two alleles present.
Population 71 contained 0 incompatible loci -- more than two alleles present.
Population 72 contained 0 incompatible loci -- more than two alleles present.
Population 73 contained 0 incompatible loci -- more than two alleles present.
Population 74 contained 0 incompatible loci -- more than two alleles present.
Population 75 contained 0 incompatible loci -- more than two alleles present.
Population 76 contained 0 incompatible loci -- more than two alleles present.
Population 77 contained 0 incompatible loci -- more than two alleles present.
Population 86 contained 0 incompatible loci -- more than two alleles present.
Population 87 contained 0 incompatible loci -- more than two alleles present.
Population 88 contained 0 incompatible loci -- more than two alleles present.
Population 89 contained 0 incompatible loci -- more than two alleles present.
Population 90 contained 0 incompatible loci -- more than two alleles present.
Population 91 contained 0 incompatible loci -- more than two alleles present.
Population 92 contained 0 incompatible loci -- more than two alleles present.
Population 93 contained 0 incompatible loci -- more than two alleles present.
Distribution of the number of SNPs per locus.

#n_snps	n_loci
0	1774
1	3836
2	2916
3	2067
4	1465
5	1004
6	696
7	424
8	323

For Review Only

9	231
10	106
11	87
12	61
13	37
14	28
15	26
16	18
17	5
18	6
19	4
20	5
21	2
22	1
23	1
24	1
25	0
26	0
27	1
28	0
29	1

Populations log for dataset 2

Distribution of population loci.
Distribution of valid loci matched to catalog locus.

# Valid samples at locus	Count
1	313602
2	65350
3	18307
4	8690
5	5498
6	4104
7	3142
8	2794
9	2283
10	2055
11	1949
12	1761
13	1652
14	1547
15	1455
16	1439
17	1342

For Review Only

18	1296
19	1283
20	1190
21	1229
22	1175
23	1156
24	1093
25	1088
26	1094
27	1030
28	1003
29	1016
30	960
31	967
32	918
33	950
34	990
35	977
36	933
37	830
38	859
39	910
40	898
41	838
42	848
43	830
44	852
45	869
46	868
47	804
48	817
49	888
50	827
51	849
52	880
53	778
54	812
55	891
56	838
57	873
58	840
59	831
60	906
61	947
62	958
63	952

64 913
65 1069
66 1128
67 1178
68 1243
69 1275
70 1335
71 1237
72 1163
73 998
74 626
75 400
76 326
Distribution of confounded loci at catalog locus.
Confounded samples at locus Count
0 493502
Distribution of missing loci at catalog loci.
Absent samples at locus Count
0 493502
Distribution of population loci after applying locus constraints.
Distribution of valid loci matched to catalog locus.
Valid samples at locus Count
45 671
46 710
47 735
48 703
49 681
50 721
51 713
52 640
53 677
54 681
55 737
56 711
57 722
58 721
59 768
60 858
61 750
62 765
63 691
64 699
65 667
66 582
67 516
68 433

For Review Only

```
69	305
70	241
71	171
72	140
73	120
74	115
75	151
76	145
# Distribution of confounded loci at catalog locus.
# Confounded samples at locus	Count
0	17940
# Distribution of missing loci at catalog loci.
# Absent samples at locus	Count
0	309
1	282
2	352
3	547
4	847
5	1123
6	1390
7	1588
8	1603
9	1763
10	1710
11	1501
12	1278
13	1122
14	858
15	598
16	425
17	289
18	173
19	96
20	51
21	21
22	9
23	4
25	1
Population 1 contained 0 incompatible loci -- more than two alleles present.
Population 2 contained 0 incompatible loci -- more than two alleles present.
Population 3 contained 0 incompatible loci -- more than two alleles present.
Population 4 contained 0 incompatible loci -- more than two alleles present.
Population 5 contained 0 incompatible loci -- more than two alleles present.
Population 6 contained 0 incompatible loci -- more than two alleles present.
Population 7 contained 0 incompatible loci -- more than two alleles present.
Population 8 contained 0 incompatible loci -- more than two alleles present.
```

For Review Only

Population 9 contained 0 incompatible loci -- more than two alleles present.
Population 10 contained 0 incompatible loci -- more than two alleles present.
Population 11 contained 0 incompatible loci -- more than two alleles present.
Population 12 contained 0 incompatible loci -- more than two alleles present.
Population 13 contained 0 incompatible loci -- more than two alleles present.
Population 14 contained 0 incompatible loci -- more than two alleles present.
Population 15 contained 0 incompatible loci -- more than two alleles present.
Population 16 contained 0 incompatible loci -- more than two alleles present.
Population 17 contained 0 incompatible loci -- more than two alleles present.
Population 18 contained 0 incompatible loci -- more than two alleles present.
Population 19 contained 0 incompatible loci -- more than two alleles present.
Population 20 contained 0 incompatible loci -- more than two alleles present.
Population 21 contained 0 incompatible loci -- more than two alleles present.
Population 22 contained 0 incompatible loci -- more than two alleles present.
Population 25 contained 0 incompatible loci -- more than two alleles present.
Population 26 contained 0 incompatible loci -- more than two alleles present.
Population 30 contained 0 incompatible loci -- more than two alleles present.
Population 31 contained 0 incompatible loci -- more than two alleles present.
Population 32 contained 0 incompatible loci -- more than two alleles present.
Population 33 contained 0 incompatible loci -- more than two alleles present.
Population 34 contained 0 incompatible loci -- more than two alleles present.
Population 35 contained 0 incompatible loci -- more than two alleles present.
Population 36 contained 0 incompatible loci -- more than two alleles present.
Population 37 contained 0 incompatible loci -- more than two alleles present.
Population 38 contained 0 incompatible loci -- more than two alleles present.
Population 39 contained 0 incompatible loci -- more than two alleles present.
Population 40 contained 0 incompatible loci -- more than two alleles present.
Population 41 contained 0 incompatible loci -- more than two alleles present.
Population 45 contained 0 incompatible loci -- more than two alleles present.
Population 46 contained 0 incompatible loci -- more than two alleles present.
Population 47 contained 0 incompatible loci -- more than two alleles present.
Population 48 contained 0 incompatible loci -- more than two alleles present.
Population 49 contained 0 incompatible loci -- more than two alleles present.
Population 50 contained 0 incompatible loci -- more than two alleles present.
Population 51 contained 0 incompatible loci -- more than two alleles present.
Population 52 contained 0 incompatible loci -- more than two alleles present.
Population 53 contained 0 incompatible loci -- more than two alleles present.
Population 54 contained 0 incompatible loci -- more than two alleles present.
Population 55 contained 0 incompatible loci -- more than two alleles present.
Population 56 contained 0 incompatible loci -- more than two alleles present.
Population 57 contained 0 incompatible loci -- more than two alleles present.
Population 58 contained 0 incompatible loci -- more than two alleles present.
Population 59 contained 0 incompatible loci -- more than two alleles present.
Population 60 contained 0 incompatible loci -- more than two alleles present.
Population 61 contained 0 incompatible loci -- more than two alleles present.
Population 62 contained 0 incompatible loci -- more than two alleles present.

For Review Only

Population 63 contained 0 incompatible loci -- more than two alleles present.
Population 64 contained 0 incompatible loci -- more than two alleles present.
Population 65 contained 0 incompatible loci -- more than two alleles present.
Population 66 contained 0 incompatible loci -- more than two alleles present.
Population 68 contained 0 incompatible loci -- more than two alleles present.
Population 69 contained 0 incompatible loci -- more than two alleles present.
Population 70 contained 0 incompatible loci -- more than two alleles present.
Population 71 contained 0 incompatible loci -- more than two alleles present.
Population 72 contained 0 incompatible loci -- more than two alleles present.
Population 73 contained 0 incompatible loci -- more than two alleles present.
Population 74 contained 0 incompatible loci -- more than two alleles present.
Population 75 contained 0 incompatible loci -- more than two alleles present.
Population 76 contained 0 incompatible loci -- more than two alleles present.
Population 77 contained 0 incompatible loci -- more than two alleles present.
Population 86 contained 0 incompatible loci -- more than two alleles present.
Population 87 contained 0 incompatible loci -- more than two alleles present.
Population 88 contained 0 incompatible loci -- more than two alleles present.
Population 89 contained 0 incompatible loci -- more than two alleles present.
Population 90 contained 0 incompatible loci -- more than two alleles present.
Population 91 contained 0 incompatible loci -- more than two alleles present.
Population 92 contained 0 incompatible loci -- more than two alleles present.
Population 93 contained 0 incompatible loci -- more than two alleles present.
Population 1 contained 0 incompatible loci -- more than two alleles present.
Population 2 contained 0 incompatible loci -- more than two alleles present.
Population 3 contained 0 incompatible loci -- more than two alleles present.
Population 4 contained 0 incompatible loci -- more than two alleles present.
Population 5 contained 0 incompatible loci -- more than two alleles present.
Population 6 contained 0 incompatible loci -- more than two alleles present.
Population 7 contained 0 incompatible loci -- more than two alleles present.
Population 8 contained 0 incompatible loci -- more than two alleles present.
Population 9 contained 0 incompatible loci -- more than two alleles present.
Population 10 contained 0 incompatible loci -- more than two alleles present.
Population 11 contained 0 incompatible loci -- more than two alleles present.
Population 12 contained 0 incompatible loci -- more than two alleles present.
Population 13 contained 0 incompatible loci -- more than two alleles present.
Population 14 contained 0 incompatible loci -- more than two alleles present.
Population 15 contained 0 incompatible loci -- more than two alleles present.
Population 16 contained 0 incompatible loci -- more than two alleles present.
Population 17 contained 0 incompatible loci -- more than two alleles present.
Population 18 contained 0 incompatible loci -- more than two alleles present.
Population 19 contained 0 incompatible loci -- more than two alleles present.
Population 20 contained 0 incompatible loci -- more than two alleles present.
Population 21 contained 0 incompatible loci -- more than two alleles present.
Population 22 contained 0 incompatible loci -- more than two alleles present.
Population 25 contained 0 incompatible loci -- more than two alleles present.
Population 26 contained 0 incompatible loci -- more than two alleles present.

For Review Only

Population 30 contained 0 incompatible loci -- more than two alleles present.
Population 31 contained 0 incompatible loci -- more than two alleles present.
Population 32 contained 0 incompatible loci -- more than two alleles present.
Population 33 contained 0 incompatible loci -- more than two alleles present.
Population 34 contained 0 incompatible loci -- more than two alleles present.
Population 35 contained 0 incompatible loci -- more than two alleles present.
Population 36 contained 0 incompatible loci -- more than two alleles present.
Population 37 contained 0 incompatible loci -- more than two alleles present.
Population 38 contained 0 incompatible loci -- more than two alleles present.
Population 39 contained 0 incompatible loci -- more than two alleles present.
Population 40 contained 0 incompatible loci -- more than two alleles present.
Population 41 contained 0 incompatible loci -- more than two alleles present.
Population 45 contained 0 incompatible loci -- more than two alleles present.
Population 46 contained 0 incompatible loci -- more than two alleles present.
Population 47 contained 0 incompatible loci -- more than two alleles present.
Population 48 contained 0 incompatible loci -- more than two alleles present.
Population 49 contained 0 incompatible loci -- more than two alleles present.
Population 50 contained 0 incompatible loci -- more than two alleles present.
Population 51 contained 0 incompatible loci -- more than two alleles present.
Population 52 contained 0 incompatible loci -- more than two alleles present.
Population 53 contained 0 incompatible loci -- more than two alleles present.
Population 54 contained 0 incompatible loci -- more than two alleles present.
Population 55 contained 0 incompatible loci -- more than two alleles present.
Population 56 contained 0 incompatible loci -- more than two alleles present.
Population 57 contained 0 incompatible loci -- more than two alleles present.
Population 58 contained 0 incompatible loci -- more than two alleles present.
Population 59 contained 0 incompatible loci -- more than two alleles present.
Population 60 contained 0 incompatible loci -- more than two alleles present.
Population 61 contained 0 incompatible loci -- more than two alleles present.
Population 62 contained 0 incompatible loci -- more than two alleles present.
Population 63 contained 0 incompatible loci -- more than two alleles present.
Population 64 contained 0 incompatible loci -- more than two alleles present.
Population 65 contained 0 incompatible loci -- more than two alleles present.
Population 66 contained 0 incompatible loci -- more than two alleles present.
Population 68 contained 0 incompatible loci -- more than two alleles present.
Population 69 contained 0 incompatible loci -- more than two alleles present.
Population 70 contained 0 incompatible loci -- more than two alleles present.
Population 71 contained 0 incompatible loci -- more than two alleles present.
Population 72 contained 0 incompatible loci -- more than two alleles present.
Population 73 contained 0 incompatible loci -- more than two alleles present.
Population 74 contained 0 incompatible loci -- more than two alleles present.
Population 75 contained 0 incompatible loci -- more than two alleles present.
Population 76 contained 0 incompatible loci -- more than two alleles present.
Population 77 contained 0 incompatible loci -- more than two alleles present.
Population 86 contained 0 incompatible loci -- more than two alleles present.
Population 87 contained 0 incompatible loci -- more than two alleles present.

For Review Only

Population 88 contained 0 incompatible loci -- more than two alleles present.
Population 89 contained 0 incompatible loci -- more than two alleles present.
Population 90 contained 0 incompatible loci -- more than two alleles present.
Population 91 contained 0 incompatible loci -- more than two alleles present.
Population 92 contained 0 incompatible loci -- more than two alleles present.
Population 93 contained 0 incompatible loci -- more than two alleles present.
Distribution of the number of SNPs per locus.
#n_snps n_loci
0 1774
1 13352

Populations log for dataset 3

Note: Individual distributions can be extracted using the `stacks-dist-extract` utility.
e.g. `stacks-dist-extract populations.log.distribs dist_name`

BEGIN batch_progress
C01: analyzed 19835 loci; filtered 230810 loci; 250645 loci seen.
1931548 genomic sites, of which 56414 were covered by multiple loci (2.9%).
C02: analyzed 22724 loci; filtered 259151 loci; 281875 loci seen.
2206248 genomic sites, of which 70104 were covered by multiple loci (3.2%).
C03: analyzed 30062 loci; filtered 351403 loci; 381465 loci seen.
2926008 genomic sites, of which 85985 were covered by multiple loci (2.9%).
C04: analyzed 21664 loci; filtered 249411 loci; 271075 loci seen.
2092935 genomic sites, of which 74845 were covered by multiple loci (3.6%).
C05: analyzed 17250 loci; filtered 191665 loci; 208915 loci seen.
1672712 genomic sites, of which 53943 were covered by multiple loci (3.2%).
C06: analyzed 21088 loci; filtered 240522 loci; 261610 loci seen.
2046130 genomic sites, of which 65484 were covered by multiple loci (3.2%).
C07: analyzed 25390 loci; filtered 288384 loci; 313774 loci seen.
2470551 genomic sites, of which 71939 were covered by multiple loci (2.9%).
C08: analyzed 21791 loci; filtered 247627 loci; 269418 loci seen.
2124977 genomic sites, of which 57141 were covered by multiple loci (2.7%).
C09: analyzed 20756 loci; filtered 240403 loci; 261159 loci seen.
2015736 genomic sites, of which 63168 were covered by multiple loci (3.1%).
END batch_progress

BEGIN samples_per_loc_prefilters
Distribution of valid samples matched to a catalog locus prior to filtering.
n_samples n_loci
1 419184
2 231523
3 196563
4 177894
5 161952
6 145719

For Review Only

7	126487
8	111201
9	98979
10	87384
11	78741
12	72739
13	69163
14	62672
15	56675
16	50546
17	44467
18	38520
19	32877
20	27703
21	23793
22	20021
23	16651
24	14078
25	11455
26	9373
27	7816
28	6471
29	5255
30	4498
31	4002
32	3471
33	3163
34	2762
35	2479
36	2346
37	2106
38	2052
39	1798
40	1692
41	1650
42	1584
43	1532
44	1487
45	1428
46	1462
47	1315
48	1316
49	1268
50	1234
51	1253
52	1210

For Review Only

53	1157
54	1271
55	1138
56	1250
57	1262
58	1305
59	1267
60	1212
61	1265
62	1317
63	1342
64	1374
65	1333
66	1440
67	1484
68	1489
69	1623
70	1801
71	2015
72	2202
73	2785
74	3398
75	5041
76	7155

END samples_per_loc_prefilters

BEGIN missing_samples_per_loc_prefilters
Distribution of missing samples for each catalog locus prior to filtering.

# Absent samples at locus	Count
0	7155
1	5041
2	3398
3	2785
4	2202
5	2015
6	1801
7	1623
8	1489
9	1484
10	1440
11	1333
12	1374
13	1342
14	1317
15	1265
16	1212

For Review Only

17	1267
18	1305
19	1262
20	1250
21	1138
22	1271
23	1157
24	1210
25	1253
26	1234
27	1268
28	1316
29	1315
30	1462
31	1428
32	1487
33	1532
34	1584
35	1650
36	1692
37	1798
38	2052
39	2106
40	2346
41	2479
42	2762
43	3163
44	3471
45	4002
46	4498
47	5255
48	6471
49	7816
50	9373
51	11455
52	14078
53	16651
54	20021
55	23793
56	27703
57	32877
58	38520
59	44467
60	50546
61	56675
62	62672

For Review Only

63	69163
64	72739
65	78741
66	87384
67	98979
68	111201
69	126487
70	145719
71	161952
72	177894
73	196563
74	231523
75	419184

END missing_samples_per_loc_prefilters

BEGIN snps_per_loc_prefilters
Distribution of the number of SNPs per catalog locus prior to filtering.

n_snps	n_loci
0	2036813
1	220048
2	102200
3	54511
4	31646
5	19251
6	12286
7	8067
8	5338
9	3388
10	2338
11	1556
12	998
13	565
14	358
15	239
16	120
17	78
18	49
19	30
20	21
21	12
22	8
23	5
24	3
25	4
26	2
28	1

For Review Only

32 1
END snps_per_loc_prefilters

BEGIN samples_per_loc_postfilters
Distribution of valid samples matched to a catalog locus after filtering.

n_samples	n_loci
2	13122
3	12184
4	78240
5	308
6	4485
7	3006
8	16051
9	325
10	2454
11	1829
12	5359
13	407
14	1909
15	1474
16	2433
17	558
18	1533
19	1231
20	1373
21	600
22	1329
23	1092
24	948
25	703
26	1197
27	927
28	675
29	810
30	1036
31	745
32	516
33	888
34	932
35	647
36	434
37	967
38	921
39	520
40	447
41	1051

For Review Only

42	924
43	359
44	524
45	1159
46	720
47	244
48	661
49	1161
50	589
51	173
52	828
53	1174
54	534
55	114
56	1188
57	1123
58	375
59	65
60	1580
61	1060
62	249
63	33
64	2218
65	957
66	136
67	17
68	3278
69	719
70	72
71	3
72	5036
73	457
74	9
76	7155

END samples_per_loc_postfilters

BEGIN missing_samples_per_loc_postfilters
Distribution of missing samples for each catalog locus after filtering.

# Absent samples at locus	Count
0	7155
2	9
3	457
4	5036
5	3
6	72
7	719

For Review Only

8	3278
9	17
10	136
11	957
12	2218
13	33
14	249
15	1060
16	1580
17	65
18	375
19	1123
20	1188
21	114
22	534
23	1174
24	828
25	173
26	589
27	1161
28	661
29	244
30	720
31	1159
32	524
33	359
34	924
35	1051
36	447
37	520
38	921
39	967
40	434
41	647
42	932
43	888
44	516
45	745
46	1036
47	810
48	675
49	927
50	1197
51	703
52	948
53	1092

For Review Only

54	1329
55	600
56	1373
57	1231
58	1533
59	558
60	2433
61	1474
62	1909
63	407
64	5359
65	1829
66	2454
67	325
68	16051
69	3006
70	4485
71	308
72	78240
73	12184
74	13122

END missing_samples_per_loc_postfilters

BEGIN snps_per_loc_postfilters
Distribution of the number of SNPs per catalog locus (after filtering).

n_snps	n_loci
0	147021
1	21457
2	11928
3	7243
4	4795
5	3005
6	1886
7	1236
8	765
9	498
10	278
11	179
12	111
13	73
14	41
15	21
16	11
17	3
18	3
19	6

For Review Only

END snps_per_loc_postfilters

For Review Only

Title: Supplementary Information for: Feral populations of *Brassica oleracea* along Atlantic coasts in western Europe

Table 1: Information about *Brassica oleracea* plants from the Atlantic coasts in western Europe from double-digest restriction-site associated DNA sequencing, including: the number of reads obtained from sequencing, the number of reads that mapped to the *B. oleracea* genome and the percentage of reads that mapped to the *B. oleracea* genome.

Region	Population	Number of reads	Number of reads mapped	Percentage of reads mapped
East Scotland	Auchmithie	1387753	1207726	87.03
East Scotland	Auchmithie	1567016	1357070	86.60
East Scotland	Auchmithie	1464345	1268561	86.63
East Scotland	Auchmithie	1621739	1403811	86.56
East Scotland	Crail	1595187	1378333	86.41
East Scotland	Crail	1468764	1288648	87.74
East Scotland	Crail	1592711	1400314	87.92
East Scotland	Crail	1427818	1261920	88.38
North-east Scotland	Fortrose	1793049	1492860	83.26
North-east Scotland	Fortrose	745	566	75.97
North-east Scotland	Fortrose	1418162	1169961	82.50
North-east Scotland	Fortrose	1015983	871504	85.78
West Scotland	Kildonan Castle	1920023	1675933	87.29
West Scotland	Kildonan Castle	827008	725225	87.69
West Scotland	Kildonan Castle	863404	753105	87.23
West Scotland	Kildonan Castle	384814	334230	86.85
North-east England	Tynemouth	5361799	4611195	86.00
North-east England	Tynemouth	730526	627536	85.90
North-east England	Tynemouth	1345547	1151687	85.59
North-east England	Tynemouth	2928179	2492162	85.11
North-east England	Staithes	1147	953	83.09
North-east England	Staithes	873	718	82.25
North-east England	Staithes	1443	1247	86.42
North-east England	Staithes	910	752	82.64
North-east England	Whitby	1126643	972813	86.35
North-east England	Whitby	2268574	1943089	85.65
North-east England	Whitby	1119790	977734	87.31

For Review Only

Table 1: Information about sequencing of *Brassica oleracea* plants cont.

Region	Population	Number of reads	Number of reads mapped	Percentage of reads mapped
North-east England	Whitby	1063470	905937	85.19
North Wales	Little Orme	410	332	80.98
North Wales	Little Orme	424	356	83.96
North Wales	Little Orme	470	389	82.77
North Wales	Little Orme	601	479	79.70
South Wales	Tenby	1462901	1245452	85.14
South Wales	Tenby	2222716	1882245	84.68
South Wales	Tenby	1593699	1340950	84.14
South Wales	Tenby	1282352	1083760	84.51
South Wales	Llantwit Major	1329946	1146093	86.18
South Wales	Llantwit Major	1396788	1210897	86.69
South Wales	Llantwit Major	1919399	1650157	85.97
South Wales	Llantwit Major	2591684	2222948	85.77
South-west England	Prussia cove	1619523	1387691	85.69
South-west England	Prussia cove	1772075	1513752	85.42
South-west England	Prussia cove	1306856	1127550	86.28
South-west England	Prussia cove	930252	801321	86.14
South-west England	Fowey	1202859	1043323	86.74
South-west England	Fowey	2800428	2420953	86.45
South-west England	Fowey	1096937	951131	86.71
South-west England	Fowey	655043	568748	86.83
South-west England	West Looe	526143	456982	86.86
South-west England	West Looe	1275394	1099013	86.17
South-west England	St Aldeham's Head	1996066	1733925	86.87
South-west England	St Aldeham's Head	1826145	1597182	87.46
South-west England	St Aldeham's Head	1373774	1199240	87.30
South-west England	St Aldeham's Head	1735302	1506195	86.80
Asturias	Cudillero	298891	263713	88.23
Asturias	Cudillero	1296387	1139395	87.89
Asturias	Cudillero	947934	835735	88.16
Asturias	Cudillero	3575040	3158715	88.35
Asturias	Playa de Xágo	1812681	1573772	86.82
Asturias	Playa de Xágo	1162553	1003317	86.30

For Review Only

Table 1: Information about sequencing of *Brassica oleracea* plants cont.

Region	Population	Number of reads	Number of reads mapped	Percentage of reads mapped
Asturias	Playa de Xágo	578767	495639	85.64
Asturias	Playa de Xágo	1278240	1082892	84.72
Asturias	Cabo de Peñas	1028128	893090	86.87
Asturias	Cabo de Peñas	1268678	1082179	85.30
Asturias	Cabo de Peñas	2897986	2476276	85.45
Asturias	Cabo de Peñas	413511	359603	86.96
Asturias	Playas de Viodo	482032	422063	87.56
Asturias	Playas de Viodo	737102	652759	88.56
Asturias	Playas de Viodo	220363	192283	87.26
Asturias	Playas de Viodo	721625	624490	86.54
Asturias	Tazones	710	601	84.65
Asturias	Tazones	1922	1587	82.57
Asturias	Tazones	1070	918	85.79
Cantabrica	Playa Pedrero	1009564	867686	85.95
Cantabrica	Playa Pedrero	1298664	1129231	86.95
Cantabrica	Playa Pedrero	3058101	2633809	86.13
Cantabrica	Playa Pedrero	1414237	1221669	86.38
Cantabrica	La Franca	604	509	84.27
Cantabrica	La Franca	466	379	81.33
Cantabrica	La Franca	520	433	83.27
Basque	San Juan de Gaxtelugatxe	1444693	1253303	86.75
Basque	San Juan de Gaxtelugatxe	3135997	2723927	86.86
Basque	San Juan de Gaxtelugatxe	1527228	1315084	86.11
Basque	San Juan de Gaxtelugatxe	2534715	2178782	85.96
Basque	Getaría	1419546	1232331	86.81
Basque	Getaría	1146516	994255	86.72
Basque	Getaría	1325400	1133457	85.52
Basque	Getaría	1304818	1117297	85.63
Basque	San Sebastian	1203388	1051422	87.37
Basque	San Sebastian	2227159	1921417	86.27
Basque	San Sebastian	898	612	68.15
Basque	San Sebastian	964	798	82.78

For Review Only

Table 1: Information about sequencing of *Brassica oleracea* plants cont.

Region	Population	Number of reads	Number of reads mapped	Percentage of reads mapped
Basque	San Sebastian	2768379	2426913	87.67

Table 2: Number and percentage of missing SNPs for each population in dataset 1, which contains 42,517 SNPs.

Region	Population	Number of SNPs	Number of missing SNPs	Percentage of missing SNPs
East Scotland	Auchmithie	35284	7233	17.0
East Scotland	Auchmithie	34909	7608	17.9
East Scotland	Auchmithie	35374	7143	16.8
East Scotland	Auchmithie	35936	6581	15.5
East Scotland	Crail	37553	4964	11.7
East Scotland	Crail	37964	4553	10.7
East Scotland	Crail	36184	6333	14.9
East Scotland	Crail	35580	6937	16.3
North-east Scotland	Fortrose	31267	11250	26.5
North-east Scotland	Fortrose	24585	17932	42.2
North-east Scotland	Fortrose	24019	18498	43.5
West Scotland	Kildonan Castle	37569	4948	11.6
West Scotland	Kildonan Castle	25978	16539	38.9
West Scotland	Kildonan Castle	24633	17884	42.1
West Scotland	Kildonan Castle	8667	33850	79.6
North-east England	Tynemouth	39926	2591	6.1
North-east England	Tynemouth	11336	31181	73.3
North-east England	Tynemouth	23338	19179	45.1
North-east England	Tynemouth	35321	7196	16.9
North-east England	Whitby	32574	9943	23.4
North-east England	Whitby	38987	3530	8.3
North-east England	Whitby	33152	9365	22.0
North-east England	Whitby	30207	12310	29.0
South Wales	Tenby	27058	15459	36.4
South Wales	Tenby	30274	12243	28.8
South Wales	Tenby	22793	19724	46.4
South Wales	Tenby	25416	17101	40.2

For Review Only

Table 2: Information about dataset 1 SNPs cont.

Region	Population	Number of SNPs	Number of missing SNPs	Percentage of missing SNPs
South Wales	Llantwit Major	34529	7988	18.8
South Wales	Llantwit Major	35783	6734	15.8
South Wales	Llantwit Major	37567	4950	11.6
South Wales	Llantwit Major	38861	3656	8.6
South-west England	Prussia cove	35111	7406	17.4
South-west England	Prussia cove	33978	8539	20.1
South-west England	Prussia cove	30779	11738	27.6
South-west England	Prussia cove	17982	24535	57.7
South-west England	Fowey	28968	13549	31.9
South-west England	Fowey	39332	3185	7.5
South-west England	Fowey	28774	13743	32.3
South-west England	Fowey	16316	26201	61.6
South-west England	West Looe	25330	17187	40.4
South-west England	West Looe	33283	9234	21.7
South-west England	St Aldeham's Head	38935	3582	8.4
South-west England	St Aldeham's Head	38055	4462	10.5
South-west England	St Aldeham's Head	36817	5700	13.4
South-west England	St Aldeham's Head	36176	6341	14.9
Asturias	Cudillero	11931	30586	71.9
Asturias	Cudillero	35501	7016	16.5
Asturias	Cudillero	3294	9593	22.6
Asturias	Cudillero	39800	2717	6.4
Asturias	Playa de Xágo	37756	4761	11.2
Asturias	Playa de Xágo	33977	8540	20.1
Asturias	Playa de Xágo	19052	23465	55.2
Asturias	Playa de Xágo	32265	10252	24.1
Asturias	Cabo de Peñas	33211	9306	21.9
Asturias	Cabo de Peñas	29122	13395	31.5
Asturias	Cabo de Peñas	38730	3787	8.9
Asturias	Cabo de Peñas	8223	34294	80.7
Asturias	Playas de Viodo	11282	31235	73.5
Asturias	Playas de Viodo	26662	15855	37.3
Asturias	Playas de Viodo	1731	40786	95.9

For Review Only

Table 2: Information about dataset 1 SNPs cont.

Region	Population	Number of SNPs	Number of missing SNPs	Percentage of missing SNPs
Asturias	Playas de Viodo	19420	23097	54.3
Cantabrica	Playa Pedrero	31359	11158	26.2
Cantabrica	Playa Pedrero	35116	7401	17.4
Cantabrica	Playa Pedrero	39848	2669	6.3
Cantabrica	Playa Pedrero	36310	6207	14.6
Basque	San Juan de Gaxtelugatxe	34359	8158	19.2
Basque	San Juan de Gaxtelugatxe	39746	2771	6.5
Basque	San Juan de Gaxtelugatxe	33073	9444	22.2
Basque	San Juan de Gaxtelugatxe	37287	5230	12.3
Basque	Getaría	34975	7542	17.7
Basque	Getaría	28516	14001	32.9
Basque	Getaría	31536	10981	25.8
Basque	Getaría	33376	9141	21.5
Basque	San Sebastian	34737	7780	18.3
Basque	San Sebastian	39028	3489	8.2
Basque	San Sebastian	39569	2948	6.9

For Review Only

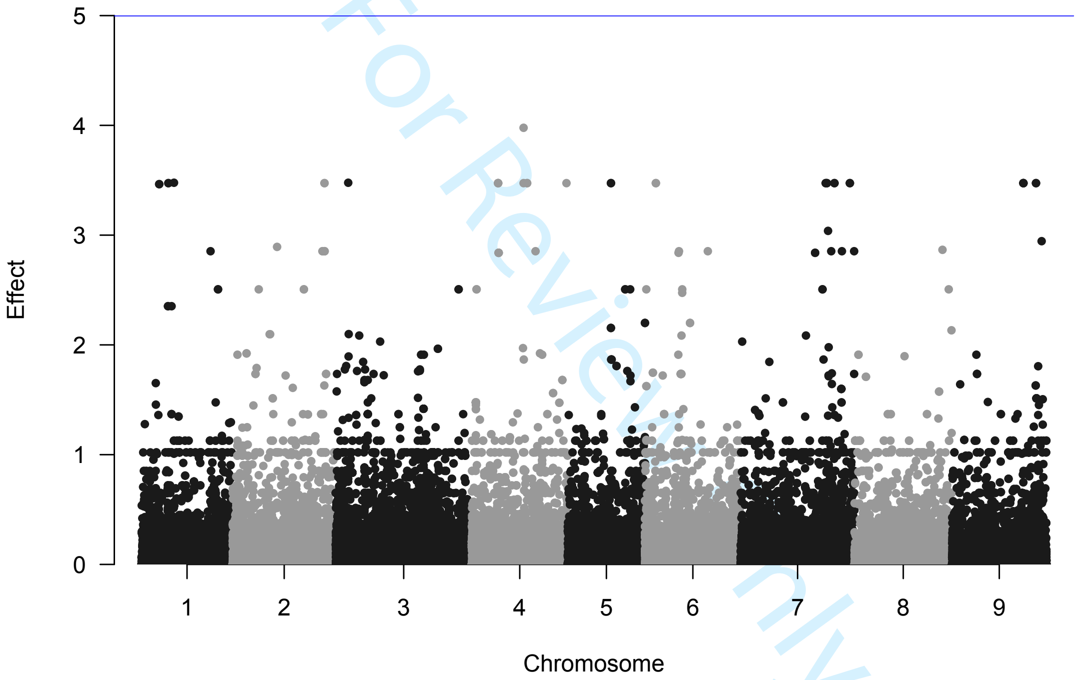

For Review Only

Figure 1: Manhattan plot showing the distribution of SNPs from ddRAD sequencing of *B. oleracea* plants from natural populations in western Europe mapped to the *B. oleracea* genome from Liu *et al.* 2014.

Mittell *et al.* Response to reviewers July 2020

Reviewer Comments to Author:

Reviewer: 1

Comments to the Author
In this ms Mittell et al examine the population structure of Brassica oleracea putatively wild populations from the Atlantic range of the species, with the aim of discerning their wild or feral origin, as well as to identify signatures of local adaptation. For this, they obtained ca. 13,000 ddRAD loci on 76 individuals of 20 populations of UK and Spain. The ms is nicely written and tells a congruent story. The molecular and bioinformatic methods and data analyses are also well described and employed. My only major concern is that the sampling design is not adequate to test the main driving question of the ms, as I explain further below.

MAJOR
The main question of the ms is what is the mostly likely origin (wild or feral) of wild B. oleracea in the Atlantic region. To examine this, the authors perform phylogenetic and population structure analyses with a comprehensive sampling of these potentially wild populations, even incorporating data on the first local records of the plants. This is all very good and informative, and I agree with the way the data was analysed and discussed under the umbrella of the driving question. However, in my opinion results like the weird no-isolation-by-distance could also be explained by an intricate phylogeographic history, and not necessarily by human movement of populations. Therefore, I think it would be much more robust to include cultivar samples (specially kale as mentioned in l. 362) and an outgroup in the analyses. Cultivated samples would be expected to be a subset of the wild variation, and feral populations (despite how "wild" they look phenotypically) would be expected to be a subset of the cultivated variation, as has been shown in other wild-domesticated species complexes.

I know it may be unrealistic for the work group to sample and sequence again, so I dare not ask for that. But given that this is a well studied crop, there is already available data on cultivated forms (e.g. https://www.nature.com/articles/s41438-018-0040-3) that with some bioinformatics wrangling could be made comparable with the present dataset, at least to obtain enough loci to perform a phylogenetic analyses.

If, for whatever reason, incorporating published data from domesticated forms is not possible, I think the authors should at least acknowledge this caveat and re-focus the paper to make the wild-feral point less central. I'm sure this is possible, given that the dataset is very good and relevant to CWR research.
Reply: Pages 6 and 15. Thank you for your comments here. We have downplayed focus on the question of origins by: (1) being more cautious about what we can conclude in the discussion; and (2) altering our objectives to focus more explicitly on investigating genetic variation and population structure within the two focal regions sampled. We acknowledge the lack of cultivars in the discussion (lines 384--387) by saying explicitly that it would be interesting to add to our work by identifying, sequencing and comparing results with local cultivars. There were some cultivars included in the original sequencing. However, when some of the sequencing failed (the reason for not all the natural localities being included), the cultivar samples were unfortunately amongst these. Since our main focus was on comparing the wild populations in the UK and Spain, we had maximised the number of populations and samples that could be included from those. In the future we agree that it would be useful to include many more cultivars to cover this aspect comprehensively and avoid issues with loss of samples during sequencing.

Carrying out another study with the additional analyses suggested would be a good place to start for future work. Using published data is an intriguing idea but combining genotype by sequencing data generated using different restriction enzymes would not be trivial. Instead, we have refocused the aims of the paper to emphasise adding knowledge about diversity in the wild.

DATA ACCESSIBILITY

Please notice that raw data should be deposited at SRA (not Dryad), and that your Dryad repository should contain Stacks genotypes output (along with any other relevant to downstream analyses) and scritps (bash scripts for processing data and R scripts for the mentioned analyses).
Reply: Page 22. Thank you, we have deposited the raw sequencing data and associated meta data on the European Nucleotide Archive under the study accession number: PRJEB38464 (\url{http://www.ebi.ac.uk/ena/data/view/PRJEB38464}).

MINOR

* The introduction, specially the first paragraph, is a bit too focused on the genetic consequences of domestication due to artificial selection, and it misses to explicitly incorporate the effect of genetic drift.
Reply: Page 2. Increased genetic drift is included as an example of what can happen due to artificial selection and domestication bottlenecks in lines 46--48.

* Last paragraph of the introduction, main question addressed: I suggest changing "(2) how much population structure exists between geographically isolated populations" for "(2) what is the population structure among the Atlantic range of the species and how much differentiation exists between isolated populations?"
Reply: Page 6. We have changed our objectives to "The following questions were addressed: (1) how much genetic variation exists among wild populations of *B. olereaea* in the UK and Spain; (2) how are populations structured in the Atlantic region and how much differentiation exists between isolated populations? and (3) are there signals of local adaptation to the environment?" Lines 143--145

* It would be useful to incorporate few references to other wild-domesticated species complexes where the origin of wild/feral populations was disentangled using genomic data.
Reply: Page 4. We have added the following into the introduction: "Compared to domestication, feralization is under-investigated; however modern genomic data are allowing its occurrence to be identified and consequences better understood (see examples in Henriksen 2018)." Line 84--86

* L 338-353. This paragraph feels a bit disconnected from your results, specially the last sentence. Please discuss the microsatellite and nDNA previous results in light of your ddRAD data. Also notice that you could extract cpDNA from your dataset.
Reply: Page 15. We are sorry that we hadn't made the connection to our results clear enough. Particularly since the previous chloroplast data were based on microsatellites, it would be difficult to extract from the ddRAD data, which filter out repeats because of the problems of interpreting when considering only 100 bp loci. Therefore, we have reduced this paragraph and included more specific comparison with our results. Lines 388--399.
"The two populations with the C:04 haplotype were in Tyne & Wear, in the northeast of England; in the current study, this area is represented by the Tynemouth and Whitby populations. In line with the rarity of the chloroplast haplotypes identified in this region in the previous study, these two populations clustered most closely with populations not sampled by Allender et al. (2007);…"

Thanks for the opportunity of review your work. I enjoyed it, and hope these comments help to improve it.

Reviewer: 2

Comments to the Author
Mittell et al. report analyses of feral populations of Brassica oleracea using sub-genomic sequencing (RAD-seq) paired-end reads generated from individuals distributed along the coasts of UK and Spain. They concluded that the feral populations recently expanded from domesticated varieties and there might be a possible connection between genetic diversity and local soil pH.

The following comments should be addressed.
1. The authors presented a background about domestication and resulting bottleneck and reduced genetic diversity; on the other hand, they also indicated that B. oleracea includes a great degree morphological diversity. The study here focused on feral populations, thus it is not clear how the results can be used to address questions about genetic diversity in domesticated crop varieties. It seems that there is a disconnect between the early parts of the introduction and the actual study of this paper.
Reply: We had not intended to imply that our data could be used to understand genetic diversity in domesticated crops. However, we do think that the patterns of genetic diversity and population structuring are intriguing in relation to understanding how much variation exists in “wild” populations. We have revised the abstract, introduction and discussion to make it clearer that our focus was on the wild populations, rather than understanding more about domesticated crops.

2. The author also discussed different hypotheses of the origins of the feral populations in the Atlantic regions in north-western Europe, and that the most plausible hypothesis is that the feral populations were derived from domestic crop varieties. Therefore, the conclusion is largely confirmatory, lacking sufficient originality.
Reply: Page 3. Although there is evidence from other sources, there has been a lack of genetic evidence for either hypothesis. We hope by adding the following to the end of this paragraph on line 73 in the introduction, that this has been made clearer: “However, the genetic status of *B. oleracea* in the Atlantic region is still an open question (*B. oleracea* is classified as a native species in the UK and an alien species in Spain; Euro+Med PlantBase 2020).”

3. The authors stated that dataset 2 contain one SNP per each RAD locus, to obtain unlinked loci. However, they also reported that the number of SNPs is 13158 in dataset 2. It seems very puzzling how over 13000 loci could be unlinked for a species with a genetic map of about 900 cM. Did the authors mean something else when they said “unlinked”?
Reply: Page 7. Thank you for pointing out the potential confusion here. This has been changed to: "SNPs linked within each RAD locus were avoided by only retaining one SNP at random per locus;" line 191

4. If one hypothesis to be tested is that feral populations were derived from domesticated varieties, the study should include a number of cultivated varieties, especially those that have been grown in UK and Spain during the history of cultivation in these regions.
Reply: We agree that carrying out the same sequencing on a range of cultivated varieties from the local regions would enhance our work in the future. As described in the response to reviewer 1, there were some cultivars included in the original sequencing. However, some of the sequencing failed (the reason for not all the natural localities being included), and cultivar samples were unfortunately amongst these. We still consider the implications of our results for testing this

For Review Only

hypothesis but downplay what can be concluded without sequencing of a wide range of cultivars. We have modified the aims and restructured the discussion to focus more clearly on genetic diversity and population structure within the "wild" populations.

5. The analysis here used the reference B. oleracea genome to identify genetic variations among individuals of feral populations. Could there be sequences from these wild individuals that are too divergent from the reference genome to be mapped? This idea is at least in principle supported by the observation that 11.6-31.8% of reads were not mapped to the B. oleracea. Is it possible that some of these reads are highly divergent from the reference genome sequence?
Reply: This is a good point and as more reference genomes become available then it would be worth re-running analyses such as ours. However, since the main focus of our study was to use ddRAD to identify shared SNPs that could be used for population genetics analyses, excluding highly divergent regions might actually be preferable (e.g., to avoid regions under divergent selection).

6. If such sequences exist, could some of the divergent sequences reveal additional population structural information regarding these individuals? For example, could a subset of these individuals exhibit great similarities or differences than the analyses here have shown? If yes, such additional information could alter the conclusion.
Reply: This would be interesting to test but ddRAD sequencing might not be the best approach to use for this type of analysis, due to the difficulty of testing for selection with only 100 bp and filtering to consider only one SNP per RAD locus. The population genetics analyses used assume neutrality and so the conservative approach of excluding highly divergent loci might actually help to reduce the risk of interpreting patterns based on divergent selection in different populations.

7. It is possible that de novo assembly might reveal additional sequence variation than those revealed mapping to the reference genome would have missed.
An all-against-all comparison of de novo assembled sequences might reveal additional population structure information.
Reply: We agree that de novo assembly might increase the number of loci included, which would be important for genome-wide association studies. However, the 13,352 SNPs used here (dataset 2) are suitable for assessing the population structure. Previous work has shown that there is an effect on population structure of increasing the number of SNPs at low numbers (e.g., <100 SNPs in Morin *et al.* 2009 Mol. Ecol. Resources: 9(1) 66-73; and 50 -- 3500 SNPs in Nazareno *et al.* Mol. Ecol Resources: 17(6) 1136-1147), however, the number of SNPs used here is well above the minimum number of SNPs required for the analyses carried out.

8. It would also be informative to know the map positions of the reads from the analyzed individuals, in a supplemental figure or table. For example, are the SNPs clustered in a small number of chromosomal regions, and distributed throughout the genome?
Reply: A supplementary figure has been added (and referred to in the text) to show the distribution of the SNPs from our study mapped to the reference *B. oleracea* genome. In addition, the output from stacks that contains the locations of the SNPs has been included in the supplementary material.

9. The RAD-seq data should be deposited in a public database.
Reply: Page 22. We have deposited the raw sequencing data and associated meta data on the European Nucleotide Archive under the study accession number: PRJEB38464 (\url{http://www.ebi.ac.uk/ena/data/view/PRJEB38464}).

10. Some of the data here might reveal additional genetic differences if the read pairs from the paired-end sequencing were analyzed together. For example, clusters of read pairs with longer than expected gaps in the reference genome would suggestion a deletion in the sequenced individual.

Insertions in the sequenced individual might lead to some read pairs with only one read being mapped (because the other read is in the inserted sequence). Such analyses should be performed to identify genome differences, which can be considered for population structure.
Reply: Page 8. This could be an interesting approach but we would be concerned that with only 100 bp “loci” for RAD data, reliably interpreting insertions and deletions would be quite challenging. A limitation of RAD sequencing is that allelic dropout is impossible to distinguish completely from lack of coverage. Our filtering decisions were aimed at reducing these types of errors, as described in O'Leary et al. (2018). We have added to our methods to clarify the filtering decisions that were also queried by reviewer 3. Lines 195--202.

11. Key statistics of the RAD-seq data should be reported in a supplemental table for each individual: (1) number of raw read pairs; (2) number of read pairs with mapping results with expected gap size; (3) number read mapped to the genome; (4) SNPs before filtering; (5) number of reads with SNPs; (6) number of SNPs after filtering; (7) range of sizes of RAD loci; (8) number of filter SNPs on each chromosomal arms (18 numbers), and possibly others.
Reply: We have included details about the number of reads, the number of reads that mapped to the genome, the number of SNPs and missing SNPs in supplementary tables separately. In addition, we have provided information in the supplementary from the output of running stacks to enable the reader to find any additional information that they may wish to see.

12. Some SNPs might be false positives because they are actually from two or more copies of nearly identical sequences in the genome. Sometimes sequenced individual might have more copies than the reference genome. Therefore, de novo assembly (above) can also help to cover such mistakes in SNP identification.
Reply: The filtering used was designed to reduce inclusion of duplicate loci. The minimum stack depth used was 5 (default is 2), which is in the middle range of depths usually considered for excluding loci (Paris *et al.* 2017 Methods in Ecology and Evolution: 8 1360-1373). This was used to minimise the number of paralogues (a higher value could lead to filtering out informative loci). Using a maximum observed heterozygosity of 0.7 also helps to remove potential paralogues as no more than 70% of the individuals (dataset 1 and 2) or populations (dataset 3) can be heterozygous for each locus.

13. B. oleracea and other related species have shared a genome triplication, and many paralogs are still retained. There are other duplications. These homologous sequences might also differ in copy number among individuals, and be additional challenges to correct identification of SNPs.
For example, if B. oleracea has two highly similar genes (A and B), but one of them is lost in the reference genome or not sequenced (B), and the allele of the second copy (B) in some wild individual could be treated as allelic to A if the true A allele is not detected by the RAD-seq. Mapping of both of the paired reads can reduce such mistakes, and careful analysis of multiple reads of the same locus can also help.
Reply: Page 16. This would be very useful to consider if we were attempting to reconstruct the genome sequences but ddRAD data are not the most appropriate for resolving paralogs, given the filtering to include only a single SNP per locus, a standard approach for population genetic analyses (Pritchard et al. 2000), and the short length of the “loci” between restriction sites (100 bp). Instead, duplicate loci are excluded by having a minimum stack depth of 5, a maximum observed heterozygosity of 0.7 and a minimum minor allele frequency of 0.01. In addition, we only consider those loci that are present in at least 60% of the individuals in datasets 1 & 2, and in 50% of the populations in dataset 3.

We have added a paragraph to the discussion that more explicitly describes the limitations of ddRAD sequencing for enabling comparisons with published genome sequences and other types of

genotype by sequencing data (lines 411--427). This would be particularly problematic for complex Brassciaceae genomes, precisely due to the triplication. However, for population genetic analyses, the filtering applied here was conservative enough that these issues should be reduced. Although the excess heterozygosity observed could be explained by introgression, we also more explicitly acknowledge that some could be due to merging of duplicate loci. However, the consistency in this excess across populations suggests that interpretations about relative diversity and population structure should still be meaningful.

14. Different methods for phylogenetic analysis should be use to demonstrate the strength of the results. Methods that consider conflicts among the loci are especially valuable.
Reply: Since we are considering population genetic variation, we are not expecting bifurcating trees appropriate to a rigorous phylogenetic analysis. Instead, RAXML was used for clustering, to visualise variation within and among populations. We also used Splitstree for visualisation, which explicitly considers conflicts among loci by presenting alternative pathways.

15. In addition, different subsets of the data can be used to generate the phylogeny.
Reply: We hadn't meant to imply that a phylogenetic approach was appropriate for the within-species population-level data presented. We checked the manuscript to make sure that we hadn't referred to a phylogeny. The cluster analysis presented is for visualisation of relative variation within and between populations. The level of admixture apparent in figure 1-iii (STRUCTURE plot) clearly demonstrates why a phylogenetic approach would not be appropriate.

Reviewer: 3

Comments to the Author
The study uses ddRAD genotyping in the domesticated plant species Brassica oleraceae, collected at 24 localities along the coasts of the UK and Spain, to characterize the population structure of wild 'populations' and to explore environment-genotype associations across varying climatic conditions. In general, the manuscript reads well. The data are newly generated for this study. However, there are some parts of the methods and results that need clarification (outlined below) as I could not understand important aspects of the research. Although I think the results are relevant, I don't think the manuscript is appropriate for the broad readership of Molecular Ecology.

Major comments
-I have major reservations about the sample sizes used in each 'population' as well as the designation of localities as populations. In the manuscript, each 'population' has 4 samples, but population-level statistics are very sensitive to sampling error at this small sample size, and I worry that they are unreliable. The authors should demonstrate that their analyses are robust to their sample sizes to make their conclusions more convincing. A couple of possibilities would be to drop the population sample size from 2 to 4 to see if overall patterns hold up, or using simulated data to prove that the methods are reliable with a sample size of 4. Besides, the authors treat localities as populations. A typical biological definition of a population is a group of interbreeding individuals that share time and space (Hedrick PW (2000) Genetics of Populations, 2nd edn. Jones and Bartlett, Sudbury, Massachusetts.), and most definitions involve some type if interbreeding. One of the goals of population genetics is to identify what is a population and how many are present. Many statistical tests were developed to identify populations. We need to be precise with our terminology. It would be better if you used sampling location, or site, in place of population.
Reply: Page 6. We agree with the reviewer that the sample sizes seem small initially. However, a recent study into the minimum sample sizes required for population genomic analyses (Nazareno et al 2017; Molecular Ecology Resources 17(6): 1136--1147) found that very small sample sizes are

useful for various population statistics when large numbers of SNPs are available. For example, they found that with ≥1500 SNPs from two individuals, FST and Ho could be accurately estimated, and that with four individuals He could also be reasonably estimated. Therefore, although larger sample sizes would be more ideal for estimating He, the sample sizes used here (2-4 individuals per locality and >13,000 SNPs) provide enough information to obtain an overview of the genetic structuring and shared ancestry. We have also previously used this approach to investigate population structure in wild Brassicaceae (genus *Arabidopsis*). We have added these additional supporting references to the methods (lines 153--156).

-There is almost no information given about the ddRAD loci and SNPs used for the analyses. For instance, what is the distribution of allele frequencies of the SNPs? Without this information, it is impossible to gauge whether the analyses are appropriate for the data. Furthermore, no summary statistics were provided for these data. Please provide tests for Hardy-Weinberg equilibrium, linkage disequilibrium, and tests for allelic dropout. Allelic dropout is a serious issue for RADseq data. Further, you state the SNPs are unlinked but do not say you tested for this. Please present the tests, such as LD, that told you these are unlinked.
Reply: Page 8. The use of "unlinked" was not descriptive enough. We meant to use it in the context of being unlinked within a locus, by only retaining one SNP within each locus for dataset 2, which is used in the population structure analysis. We have made the filtering steps clearer in our methods section (lines 195--202), which should remove some of the confusion.

We originally checked for HWE with PLINK but failed to acknowledge this. We have re-run the population-level analyses for dataset 3 to include the estimation of HWE within stacks. Table 3 has been updated based on this re-run, but the results did not drastically change and therefore did not alter our conclusions.

Minor comments
-The authors need to clarify how many populations and individuals were used in this study (e.g., 20 or 24 populations? 96 or 144 individuals?).
Reply: Page 6, line 162. The 144 was what was originally attempted to be sequenced, but unfortunately sequencing was not successful for many of these samples/populations. We have changed this to just include those populations where some individuals were successfully sequenced: 96 individuals (4*24). Furthermore, we have added supplementary information about the number of reads from these 96 individuals, including those excluded from downstream analyses.

-More details about the ddRADseq genomic library preparation should be given.
Reply: Pages 6 and 7. Additional information has been included in the methods lines 165--178. Specifically: "Double-digest RADseq libraries were made using a modification of the method in Wu et al. 2016 that allowed NexteraXT indexes (Illumine Corp., USA) to be used for multiplexing samples. In addition, an RYRY spacer was inserted in the adapter 3' of the Illumina sequencing primer annealing site to provide additional complexity at the start of read 1 immediately before the Sac1 sticky end. For each sample 400 ng DNA was fully digested with Sac1 and Mse1 restriction endonucleases and purified using Ampure XP beads. Illumina compatible i5 adapters were designed to ligate to the at the AGCT-3' sticky end left after Sac1 digest, and Illumina compatible i7 adapters were designed to ligate to the 5'-TA overhangs remaining after Mse1 digest. Adapter-ligation excess adapters were removed using Ampure XP beads. DNA fragments were amplified by 12 cycles of indexing PCR, purified, size selected (inserts 330-670 bp) and validated using a Tapeststion D1000 HS Screentape (Agilent Technologies Ltd). Libraries were equimolar pooled and the pool concentration was calculated after qPCR. Libraries were denatured, diluted and sequenced with 125bp paired-end reads on Illumina HiSeq 2500 using SBS High Output reagents v4 (Illumina Corp., USA)."

For Review Only

-The authors also need to provide more details about the parameters used in populations and better explain why they were used to obtain each data set. Also, authors need to justify why they choose so low values for some parameters in populations (e.g., -r and -p values). How these values can affect your results?

Reply: Page 8. The parameters used in populations were chosen to balance the amount of missing data against the number of SNPs retained keeping in mind the need to reduce inclusion of duplicate loci. We have added a paragraph to the methods to explain this more fully, lines 195-202:

"This filtering was designed to reduce the inclusion of duplicate loci and balance the amount of missing data with the number of informative loci (Andrews et al 2016). A minimum stack depth of five is higher than the default of two, but within the recommended range (Paris et al 2017), and helps to remove potential paralogues. Spurious SNPs are avoided by using a minor allele frequency of >0.01 (Marandel et al 2020), and the combination of a maximum observed heterozygosity of 0.7 (70 % of the individuals or populations can be heterozygous for each locus) which are present in either 60 % of individuals (datasets 1 and 2) or 50 % of the populations (dataset 3) retains loci that have been successfully genotyped across individuals, but are not completely heterozygous."

-I didn't understand why authors used cutadapt to demultiplex and to trimm reads since the process_radtags pipeline in Stacks does these procedures.

Reply: We used cutadapt instead of process_radtags for demultiplexing and trimming because the library preparation included an additional spacer in the adapter 3' Illumina primer. This was to allow for additional complexity in the libraries and improve the cluster identification and registration on the HiSeq. It was more straightforward to remove these using cudadapt at the time of analyses.

-I would like to know if authors had some issues with the ddRADseq genomic library, since so few reads (i.e., 16,894,310) were obtained for the entire sampling size (n=76 individuals). It is correct? If yes, the average reads per individual is 222,293 and not 1,534,680.

Reply: Page 10. Thank you for point this out, it was a typo which meant that the total number was an order of magnitude out. This has been corrected (line 249).

For Review Only
